# Substituent engineering of dynamic covalent bonds enables simultaneous enhancement of performance and recyclability

**DOI:** 10.1039/d6sc04529d

**Published:** 2026-06-22

**Authors:** Zhiyong Liu, Qian Chao, Jinyan Zhao, Ying Lin, Ping Yu, Min Chen, Shengyu Shi, Yixin Xiang, Jiangang Gao, Youwei Ma

**Affiliations:** a School of Chemistry & Chemical Engineering, Anhui University Hefei China liuzhiyong@ahpu.edu.cn; b Institute of Materials, École Polytechnique Fédérale de Lausanne (EPFL) CH-1015 Lausanne Switzerland youwei.ma@epfl.ch; c School of Chemical and Environmental Engineering, Anhui Polytechnic University Wuhu China shisy@ahpu.edu.cn; d School of Environmental and Chemical Engineering, Jiangsu Ocean University Lianyungang China; e Center for Water and Ecology, State Key Joint Laboratory of Environment Simulation and Pollution Control, School of Environment, Tsinghua University Beijing China

## Abstract

A central challenge in designing recyclable polymers lies in the trade-off between recyclability and performance. State-of-the-art strategies leveraging supramolecular interactions or dynamic covalent bonds (DCBs), either individually or in orthogonal combination, mostly end up improving one property with the compromise of the other. Here, we present a unified molecular design strategy based on substituent engineering of DCBs to simultaneously enhance service-life performance and end-of-life recyclability. By introducing a β-ketoester substituent into dynamic oxime–urethane (OU) linkages, intermolecular hydrogen bonding is strengthened while bond thermodynamics are biased toward dissociation. We show that the engineered poly(OU)s exhibit improved mechanical and thermomechanical properties (*e.g.*, 38 *vs.* 24 MPa in tensile strength; 360 *vs.* 169 MPa in Young's modulus; 36 *vs.* 20 °C in *T*_g_) compared to the control sample lacking the β-ketoester moieties. Meanwhile, efficient depolymerization is achieved under mild conditions (140 °C), enabling recovery of the constituent components in high yields *via* simple vacuum distillation, particularly with an isocyanate yield of 87%, in contrast to only 14% for the depolymerization of normal poly(OU). The materials are also thermally reprocessable and enable closed-loop recycling of carbon fiber-reinforced composites. This work establishes substituent engineering of DCBs as a general strategy to decouple recyclability and performance, offering a pathway toward high-performance circular polymer materials.

## Abstract 2

Incorporating β-ketoester into dynamic oxime–urethane bond leads to enhanced intermolecular hydrogen bonding and a lowered equilibrium constant for bond dissociation, thus improving both the performance and recyclability of the resulting networks.

## Graphical abstract

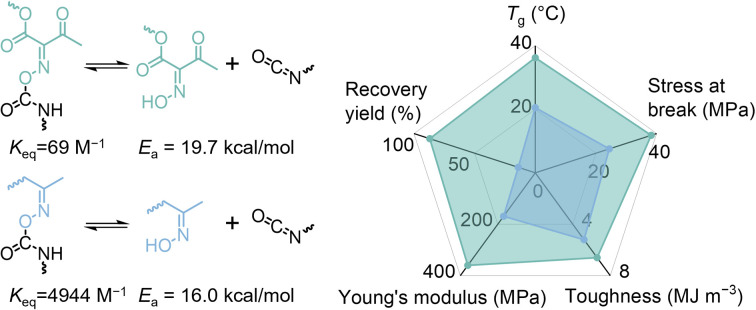

## Introduction

The replacement of petroleum-derived plastics with recyclable polymers represents one of the pillars of the transition toward a circular economy.^1–3^ However, the development of such materials is fundamentally constrained by a long-standing trade-off between recyclability and performance.^4–6^ High performance requires stable, strongly interconnected networks that restrict chain mobility, whereas recyclability relies on bond reversibility and molecular mobility, thus making it challenging to optimize both simultaneously.^7^ A representative example is that thermoplastics are intrinsically recyclable but often suffer from limited mechanical and thermal robustness, while cross-linking or filler reinforcement enhances performance at the expense of reprocessability.

State-of-the-art approaches to overcoming this dilemma consist of supramolecular engineering for thermoplastics^8–13^ or the incorporation of dynamic covalent bonds (DCBs) as reversible cross-links in thermosets.^14–19^ Supramolecular interactions (*e.g.*, hydrogen bonding, metal–ligand coordination, and host–guest interactions) can reinforce thermoplastics through reversible noncovalent bonding, yet to an extent reduce their recyclability due to strengthened intermolecular associations.^20,21^ In contrast, the recently extensively investigated DCBs, including disulfide bonds,^22–24^ cyanurates,^25–27^ tri/di-ketoenamines,^6,28–30^ vinylogous urethanes,^31–35^ and dioxaborolanes,^16,36–38^ to name a few, enable reprocessability and chemical recycling of thermosets *via* bond exchange or dissociation. They sometimes compromise thermal and mechanical properties of the materials owing to the intrinsic lability of these reversible linkages.^39^ Although the combined use of supramolecular interactions with DCBs has emerged as a promising strategy,^40–43^ these elements are typically introduced in an orthogonal manner and also end up trading one property off against the other.

This dilemma is particularly evident in polyurethane and polyurea fields. Incorporation of supramolecular interactions into linear or branched polyurethanes and polyureas can significantly enhance mechanical strength, thermal stability, and chemical resistance;^13,44,45^ however, the resulting materials often require harsher conditions for recycling or reprocessing, such as elevated temperatures, the presence of (more) catalysts, or excess depolymerization agents. In parallel, replacing conventional urethane or urea linkages with isocyanate-derived DCBs such as hindered urea,^46^ pyrazole–urea,^47^ and oxime–urethane linkages^48^ confers intrinsic recyclability and reprocessability to cross-linked networks, but sometimes at the expense of thermal and mechanical performance. Subsequent efforts to incorporate supramolecular interactions into these dynamic covalent networks (with no chemical alteration to DCBs) can improve material performance,^49,50^ yet typically lead to more demanding recycling conditions. Moreover, despite nearly a century of development in isocyanate-based polymer chemistry, direct recycling of isocyanates from their derived polymers remains exceedingly rare due to their high reactivity and propensity for side reactions.^51^

**Scheme 1:**
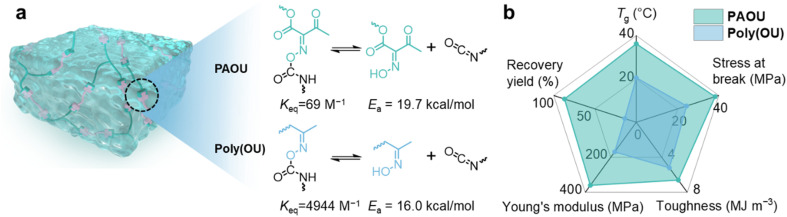
(a) Comparison of the activation energy *E*_a_ and reaction equilibrium constant *K*_eq_ for the reversible reactions of AOU and OU bonds. (b) Radar map comparing the key performance metrics and the recycling efficiency of PAOU and poly(OU) networks.

To challenge the status quo, here we propose a general molecular design strategy based on substituent engineering of DCBs. Unlike the orthogonal use of DCBs and supramolecular bonds, this strategy involves simultaneous modulation of intermolecular interactions and bond thermodynamics at the same reactive site. Specifically, we selected the well-established oxime–urethane (OU) bonds as the model system.^48,52–56^ They are functionalized with a β-ketoester motif, an essential synthon that was introduced by the Du Prez lab to the realm of recyclable polymer synthesis,^31,33,34,57^ and subsequently attracted extensive investigations, including research by some of our authors.^35,58–61^ The functionalization serves a dual and cooperative role (Scheme 1): the additional carbonyl groups enhance intermolecular hydrogen bonding,^58,62^ thereby improving thermal and mechanical properties, while the electron-withdrawing nature of the β-ketoester lowers the thermodynamic stability of the OU linkage, shifting the equilibrium toward dissociation.^63,64^ This dual-function design enables the resulting poly(acetoacetated oxime–urethane)s (PAOUs) to achieve both enhanced service-life performance and efficient end-of-life recyclability. The PAOUs exhibit improved mechanical and thermomechanical properties compared to the poly(OU) lacking the β-ketoester moiety (Scheme 1b). Small-molecule model studies and mechanistic investigations demonstrate that the acetoacetated oxime–urethane (AOU) bonds exhibit a significantly reduced equilibrium constant, favoring dissociation into isocyanates and acetoacetated oximes at equilibrium (Scheme 1a). Consequently, PAOUs can be depolymerized under mild conditions (140 °C), enabling high-yield recovery of monomers *via* simple vacuum distillation. Notably, isocyanates are recovered in up to 87% yield—a rarely reported achievement due to the high reactivity of isocyanates—whereas the poly(OU) control exhibits minimal recovery (<14%).

## Results and discussion

### Synthesis and characterization of PAOUs, and the comparison with a normal poly(OU)

**Fig. 1:**
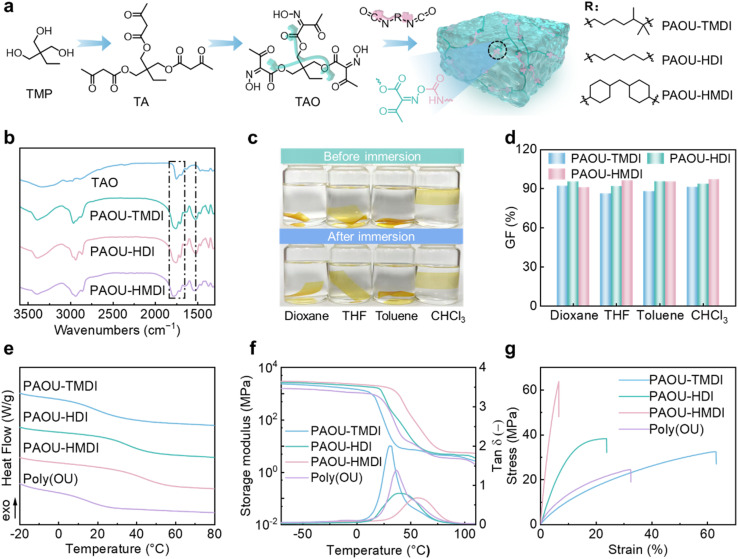
(a) Synthesis of PAOUs through the polymerization of TAO and three types of isocyanates (TMDI, HDI, and HMDI). (b) FTIR spectra of TAO and PAOUs. (c) Photographs of PAOU-HDI films dispersed in various solvents at room temperature before and after 7 days. (d) Gel fraction, (e) DSC traces, (f) DMA traces, and (g) stress–strain curves of PAOUs, and a poly(OU) synthesized through the polymerization of a normal trifunctional oxime and HDI.

The synthesis of PAOUs starts from a two-step modification (including acetoacetylation and oximation reactions; details are provided in the SI) to convert trimethylolpropane into trimethylolpropane acetoacetate oximes (TAO), followed by the cross-linking of TAO with three different di-isocyanates including TMDI, HDI, and HMDI to produce the corresponding PAOU-TMDI, PAOU-HDI, and PAOU-HMDI networks (Fig. 1a). FTIR analysis shows that the emergence of a new peak at 1520 cm^−1^ attributed to the stretching vibration of C–N in the urethane moiety, together with the shift of the ester carbonyl (C=O) stretching vibration from 1747 to 1761 cm^−1^, supports the successful formation of AOU linkages (Fig. 1b). The materials exhibit excellent chemical resistance, as reflected by non-dissolution of the PAOU-HDI films when immersed in various organic solvents including 1,4-dioxane, THF, toluene, and CHCl_3_ for 7 days (Fig. 1c). Moreover, the three PAOU networks display swelling ratios of 40–170% (Fig. S1), and regardless of the solvent type, their gel fractions are comparable, all around 90% (Fig. 1d), indicating the same level of cross-link densities present in them.

Differential Scanning Calorimetry (DSC) analysis reveals that all PAOU networks exhibit a glass transition temperature (*T*_g_) ranging between 22 °C to 44 °C (Fig. 1e). Among them, PAOU-HMDI possesses the highest *T*_g_, which primarily arises from the rigid 4,4-methylenedicyclohexyl skeleton provided by HMDI. Dynamic Mechanical Analysis (DMA) traces show a sharp drop in storage modulus *E*′, along with the emergence of a peak in the tan *δ* plots at temperatures of 31–57 °C (Fig. 1f), which marks their *T*_g_. The *T*_g_ thus measured follows the same trend as those obtained from DSC (Fig. 1e), but differ by *ca.* 10 °C in the specific value. This discrepancy is primarily attributed to the 1 Hz oscillating force employed in the DMA measurement, consistent with previous reports.^65,66^ Following the thermal transition, a rubbery regime appears in all PAOU films, and the *E*′ values of the rubbery plateau converge to approximately 4.5 MPa for all samples (Fig. 1f), reaffirming their comparable cross-link densities.

The stress–strain curves of all three PAOUs display plastic-like tensile behavior, with stress at break values of 32–64 MPa and strain at break values of 7–63% (Fig. 1g). Among them, PAOU-TMDI and PAOU-HMDI outperform their counterparts in terms of extensibility and rigidity, respectively. More specifically, varying the diisocyanate cross-linker from TMDI to HDI and HMDI leads to a progressive increase in stress at break and a decrease in strain at break (Fig. 1g). This is mainly because of the increased *T*_g_ that restricts polymer chain mobility and thereby enhances stiffness at the expense of extensibility (Fig. 1e).

Systematic comparisons of PAOU-HDI and a poly(OU) sample (lacking the β-ketoester substituent; synthesis details are provided in the SI) in thermal, thermomechanical, and mechanical properties were made. As shown in Fig. 1e–g, they demonstrate that PAOU-HDI shows higher stress at break (38 *vs.* 24 MPa), Young's modulus (360 *vs.* 169 MPa), and toughness (6.6 *vs.* 5.3 MJ m^−3^), and higher *T*_g_ determined by both DSC (36 *vs.* 20 °C) and DMA (41 *vs.* 37 °C) than those of the poly(OU) control. The performance enhancement is partially due to the presence of two carbonyl groups in AOU, which serves as hydrogen-bonding acceptors for urethane amine protons.^58,62^ We then employed Density Functional Theory (DFT) calculations (M06-2X/def2-TZVP) to investigate the hydrogen bonding interactions. It shows that the hydrogen bond between two AOUs exhibits a higher calculated intensity of 2.2 kcal mol^−1^ as compared to only 1.8 kcal mol^−1^ present in two normal OUs (Fig. S2). Thus, the increased number and intensity of hydrogen bonds jointly strengthen the polymer networks.

### Reprocessing and chemical recycling of poly(OU)s and their composites

Having confirmed the reinforcing role of the β-ketoester substituent in PAOU networks, we next investigated its impact on chemical recyclability. Existing strategies for chemically recycling polyurethanes and their analogues primarily rely on catalytic activation of urethane exchange or dissociation reactions^67–69^ or on the incorporation of isocyanate-derived DCBs.^46–48,53,58,70^ The recycling processes often proceed in the presence of a large excess of nucleophiles (*e.g.*, amines or alcohols) to selectively break the isocyanate-derived bonds upon thermal treatment. The nucleophilic substitution-induced depolymerization indeed allows the recovery of the constituent nucleophilic components of polyurethanes and their analogues, but converts isocyanate units into (more stable) downstream derivatives (Table S1).^68,69,71–73^ Since isocyanates account for a substantial fraction of the economic and environmental cost of polyurethanes, their direct recovery remains imperative.

**Fig. 2:**
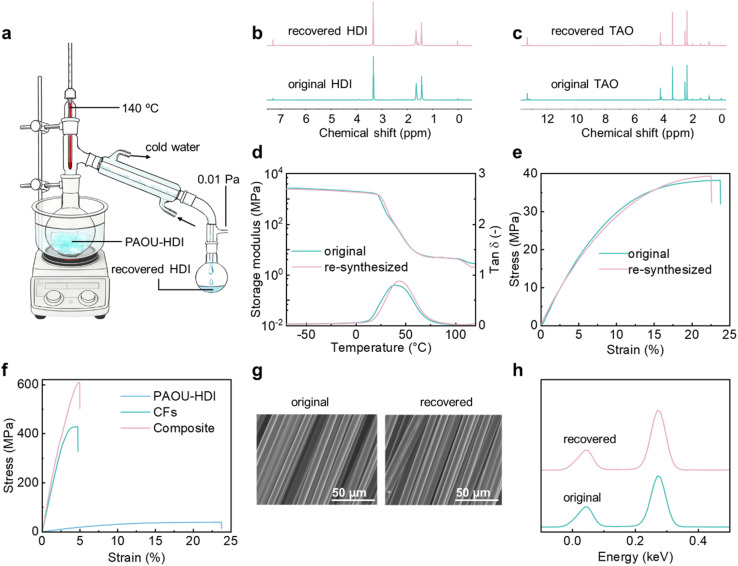
(a) Schematic illustration showing the simultaneous depolymerization of PAOU-HDI to separate both TAO and HDI by vacuum distillation. Comparison between the ^1^H NMR spectra (CDCl_3_, 400 MHz) of the original and recovered (b) HDI and (c) TAO. (d) DMA traces and (e) stress–strain curves of the original and re-synthesized PAOU-HDI. (f) Stress–strain curves of PAOU-HDI, CFs, and their composite. (g) SEM images and (h) EDX spectra of the original and recovered CFs.

The challenge of doing so arises from the high reactivity of isocyanates that can recombine with nucleophiles or undergo side reactions such as hydrolysis, biuret formation, and trimerization during depolymerization.^51^ Here, we drew inspiration from reactive distillation strategies used for recovering reactive cyclic monomers *via* ring-closing depolymerization,^65,74^ and adopted a vacuum distillation set-up to continuously separate the dissociated species. The depolymerization of PAOU-HDI was then carried out at a moderate temperature (140 °C) for 3 h under a reduced pressure of 0.01 Pa (Fig. 2a and S3). Under these conditions, recovery of both TAO and HDI in high yields of 82% and 87%, respectively, was enabled, with high chemical purity as confirmed by ^1^H NMR spectroscopy (Fig. 2b and c). The high quality of the recovered materials was further supported by their repolymerization under the same conditions for the preparation of the parent PAOU-HDI, which refurnished the polymer networks with thermomechanical and mechanical properties comparable to those of the initial ones (Fig. 2d and e). However, the same depolymerization treatment for the normal poly(OU) results in substantially lower recovery yields of oximes (11%) and HDI (14%). This marked difference highlights the significance of the β-ketoester substituent in improving the recycling efficiency of PAOU.

The good performance and closed-loop recyclability of PAOUs potentially position them as attractive polymeric substrates for the fabrication of recyclable thermosetting composites. To confirm this, we endeavored to use PAOUs to prepare engineering materials through compositing with carbon fibers (CFs), which were subsequently subjected to chemical recycling analysis. The composite was fabricated by first impregnating CFs with the reaction mixture of TAO and HDI, followed by post-curing of the prepreg in a drying oven at 40 °C for 48 h. The resulting material exhibits significant improvement in tensile strength to 607 MPa in comparison to 38 MPa for neat PAOU-HDI and 428 MPa for CFs (Fig. 2f). Noteworthily, when we applied the vacuum distillation treatment on the composite, both TAO and HDI were afforded in high yields of 76% and 83%, respectively, and in high quality (Fig. S4 and S5). Effective recovery of the valuable CFs is also achieved; SEM (Fig. 2g) and EDX (Fig. 2h, S6 and S7) analyses on the original and recycled CFs show that no distinguishable difference is noted, suggesting good retention of their morphology and chemical structure after the recycling.

Although chemical recycling is particularly advantageous for reusing CF-reinforced composites due to their non-flowable nature, thermal reprocessing remains a more energy- and resource-efficient approach.^75,76^ Thus, we subsequently explored the reprocessability of PAOU networks. Their flowability, an essential parameter for assessing polymer reprocessability and determining feasible reprocessing conditions, was initially examined by stress relaxation experiments.^31,57^ The results demonstrate that all three samples are capable of relaxing their stress in the temperature tested between 90 and 120 °C, with the stress dissipated at a faster rate at higher temperature (Fig. S8a–c). The increased stress relaxation rate primarily originates from the accelerated exchange reactions between AOUs at higher temperatures (Fig. S9a), which enable the polymer networks to rearrange themselves more easily. Arrhenius analysis of the relaxation times yields *E*_a_ for stress relaxation that is comparable across all three PAOU networks, ranging from 36.8 to 38.5 kcal mol^−1^ (Fig. S8d). This is a result of the same dynamic AOU bonds that serve as their network cross-links.

The stress-relaxation behavior guided us to reprocess the polymers by compression-molding at 100 °C under a pressure of 10 MPa for 0.5 h. The treatment refurnished homogeneous films even after being repeated three times, with the demonstration of the reprocessing of PAOU-HDI shown in Fig. S9(b). FTIR analysis, DSC and tensile testing measurements were conducted on the reprocessed PAOUs, with the results compared with those of the original materials (Fig. S9c–k). Gratifyingly, no discernible differences were observed before and after the reprocessing, confirming the excellent recovery of the chemical structures and thermal and mechanical properties, which demonstrates the good reprocessability of the materials.

### Investigation of the mechanism of the enhanced recyclability of PAOU

**Fig. 3:**
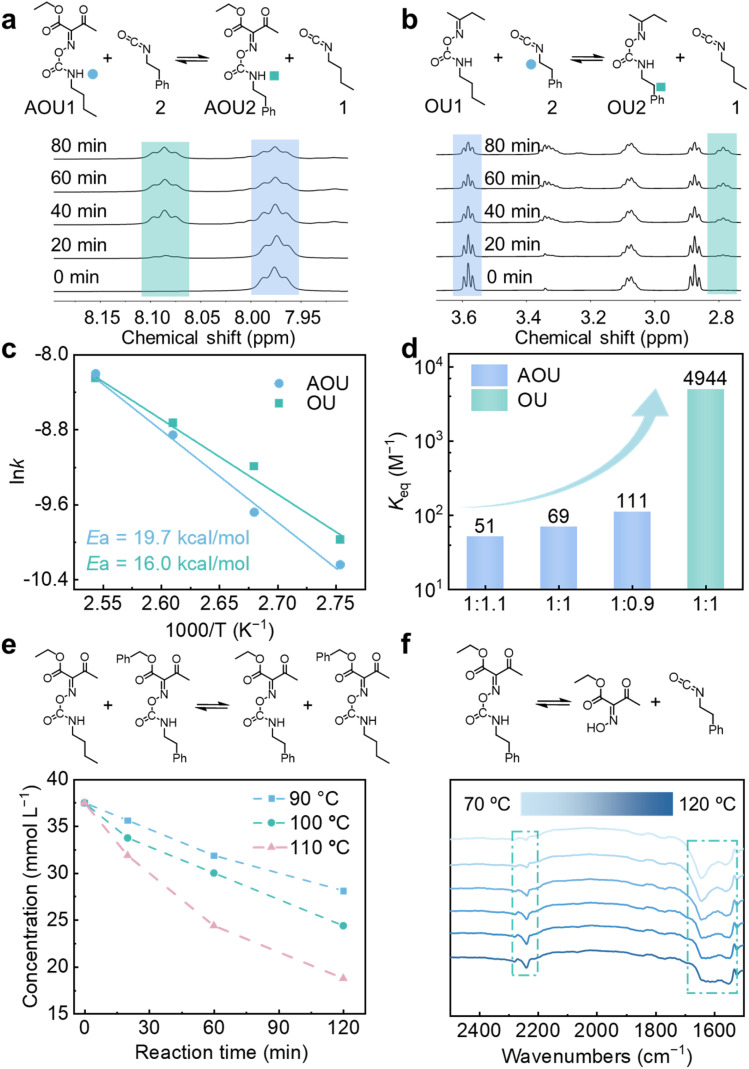
Exchange reaction between phenethyl isocyanate (2) and an equivalent amount of (a) AOU1 or (b) OU1 compound to form butyl isocyanate (1) and the corresponding AOU2 or OU2, and the relevant ^1^H NMR spectra recorded during these exchange reactions. (c) Arrhenius plots showing the logarithmic rate constants, *k*, for the conversion of AOU1 and OU1 into the corresponding AOU2 and OU2 as a function of inverse temperature. (d) *K*_eq_ for the reactions of AO and different equivalents of 1 or the reaction of BO and an equivalent amount of 1. (e) Metathesis reaction between two AOU compounds, and the conversion variation for this reaction. (f) Thermal dissociation of an AOU compound into an isocyanate and an AO, and the temperature-variable FTIR spectra of the AOU, measured from 70 °C to 120 °C, with an increment of 10 °C.

The underlying mechanism for the improved recycling efficiency present in PAOU was first analyzed by small-molecule model studies. The experiments involved reacting either a normal OU1 or an AOU1 with an equimolar amount of phenethyl isocyanate (2) (Fig. 3a and b). The reactions were conducted at 90–120 °C, and monitored by ^1^H NMR spectroscopy (Fig. 3a, b, S10 and S11). Under all conditions tested, both AOU1 and OU1 underwent efficient exchange to form the corresponding phenethyl-substituted analogues including AOU2 and OU2, accompanied by the formation of butyl isocyanate (1) (Fig. 3a and b). Kinetic analysis of the ^1^H NMR signals determined the reaction rate constants *k* (Fig. S10f and S11f). At 90 °C, the *k* values for the AOU1- and OU1-involved exchange reactions are 7.6 × 10^−5^ s^−1^ and 9.0 × 10^−5^ s^−1^, respectively, which increase to 5.5 × 10^−4^ s^−1^ and 5.2 × 10^−4^ s^−1^ at 120 °C, demonstrating the temperature dependence. Arrhenius analysis shows that the activation energies, *E*_a_, for the reactions of 2 with AOU1 and OU1 are 19.7 or 16.0 kcal mol^−1^, respectively (Fig. 3c). The slightly higher *E*_a_ value present in the 2-AOU1 reaction suggests the role of the β-ketoester moiety in improving the kinetic stability of AOU bonds.

The impact of β-ketoester on thermodynamic equilibrium of the reactions was next investigated by reacting 1 with either an acetoacetated oxime (AO) or a normal oxime, namely 2-butanone oxime (BO), at room temperature for 48 h (Fig. S12 and S13). After the reaction, BO was converted to OU nearly quantitatively, whereas the conversion of AO to AOU reached *ca.* 82%, independent of the initial stoichiometry (Fig. S12 and Tables S2–S3). Calculation of the reaction equilibrium constants, *K*_eq_, shows that varying the amount of 1 relative to AO from 0.9 to 1.1 equiv. leads to a gradual decrease in *K*_eq_ from 111 to 51 M^−1^ (Fig. 3d), probably due to competing side reactions involving isocyanates. Under stoichiometric conditions, the *K*_eq_ for AOU formation is nearly two orders of magnitude lower than that for the OU-forming reaction (69 *vs.* 4944 M^−1^). This reduced *K*_eq_ indicates that incorporation of the β-ketoester substituent shifts the equilibrium toward the dissociated state, thereby favoring the formation of free AOs and isocyanates.

We next explored the mechanism underlying the reversibility of AOU bonds. Given their structural similarity to normal OU bonds and Knoevenagel adducts, both of which are known to undergo thermally induced metathesis,^48,77–81^ we anticipated that AOUs would exhibit similar associative exchange behavior (Fig. 3e). To validate this, two AOU compounds were mixed in an equimolar ratio and heated at 90–110 °C. Reaction progress was monitored by High-Performance Liquid Chromatography (HPLC), which confirmed successful exchange between the two species, affording two additional AOU compounds (Fig. S15). The reaction rate increases with temperature, indicating thermally accelerated exchange kinetics (Fig. 3e). In parallel, the dissociative capability of AOU was assessed by temperature-variable FTIR spectroscopy. Upon heating from 70 °C to 120 °C, the characteristic C=N stretching band at 1644 cm^−1^ progressively decreases and shifts to 1630 cm^−1^, while the isocyanate absorption at 2240 cm^−1^ becomes increasingly pronounced (Fig. 3f). These spectral changes provide clear evidence for thermal dissociation of AOU bonds into isocyanate and AO groups (Fig. 3f). Taken together, these results demonstrate that AOU linkages can undergo reversible reactions through both associative and dissociative mechanisms (Fig. 3e and f).

**Fig. 4:**
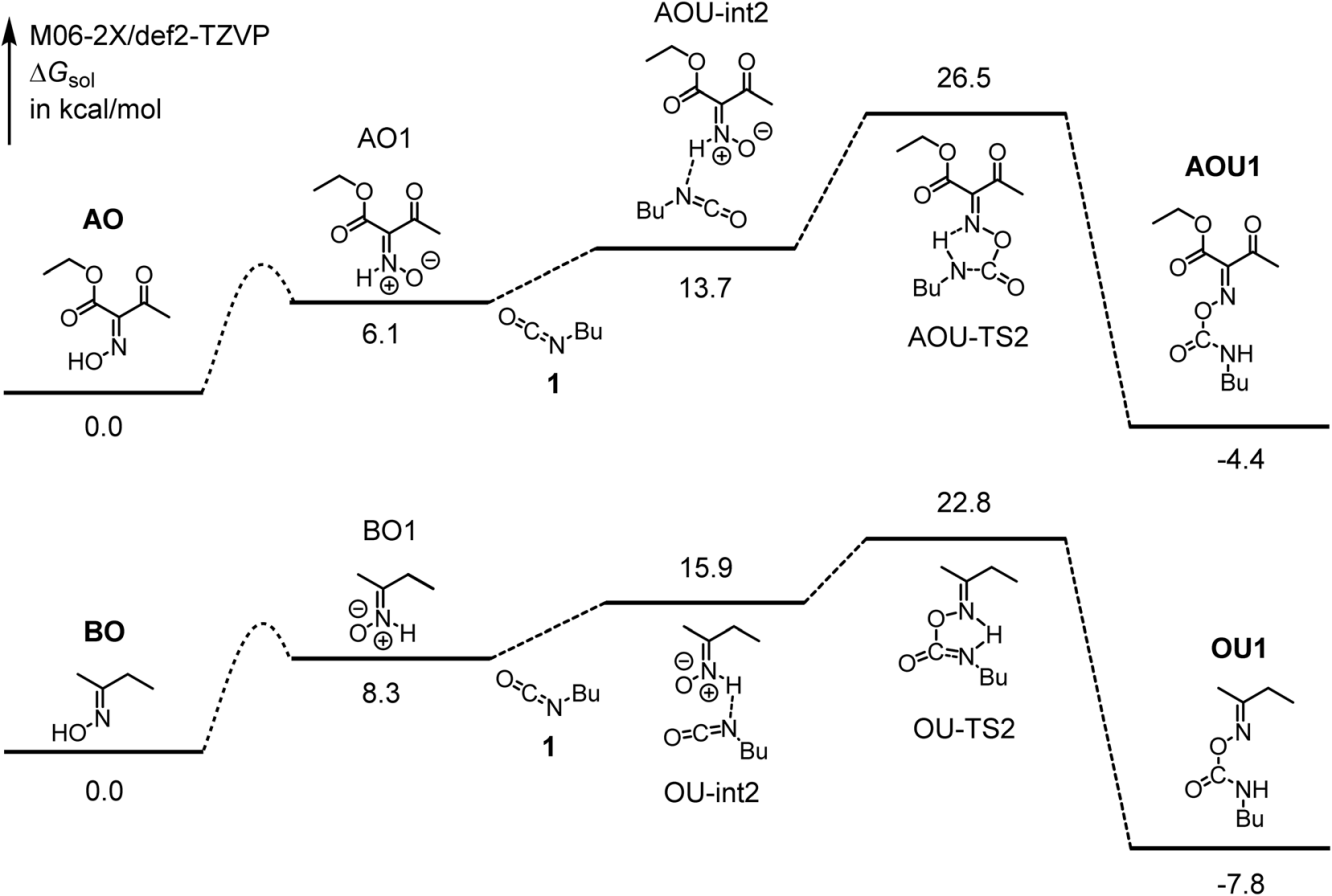
Gibbs free energies (Δ*G*^0^) of the stationary points for the reaction of isocyanate 1 with AO (top) or BO (bottom) to form AOU1 or OU1, respectively.

To further gain mechanistic insight, we then carried out DFT calculations (M06-2X/def2-TZVP) on the reactions of isocyanate 1 with AO, and also with BO as a control (Fig. 4). Prior to nucleophilic addition to 1, both AO and BO undergo isomerization to their corresponding nitrone tautomers (AO1 and BO1). For AO, this transformation proceeds *via* a unimolecular pathway facilitated by conjugation between the β-ketoester and oxime functionalities, which promotes intramolecular proton transfer (Fig. S18). In contrast, such a pathway is energetically unfavorable for BO due to the absence of the β-ketoester substituent, as evidenced by the high calculated Gibbs free energy (Δ*G*^0^) of 51.5 kcal mol^−1^ for the corresponding transition state BO-TS1 (Fig. S19). Inspired by previous studies,^48,82^ a bimolecular pathway was adopted, involving an initial association of two BO molecules to form a six-membered cyclic complex through intermolecular hydrogen bonding, followed by proton transfer within the complex and subsequent dissociation into two BO1 species (Fig. S20). This bimolecular isomerization route is chemically sound, with calculated Δ*G*^0^ not exceeding 16.6 kcal mol^−1^ along the reaction coordinate.

The nitrone intermediates AO1 and BO1 subsequently undergo nucleophilic addition to the isocyanate functionality and afford the corresponding transition states AOU-TS2 and OU-TS2, with calculated Δ*G*^0^ values of 26.5 and 22.8 kcal mol^−1^, respectively (Fig. 4). These values correspond to the *E*_a_ for AOU and OU formation, indicating slightly lower kinetics for accessing AOU. This trend is consistent with experimental observations, where the reaction rate constant for affording OU is 6.3 × 10^−5^ s^−1^, triple that (2.1 × 10^−5^ s^−1^) for AOU formation (Fig. S21). The calculated *E*_a_ values for the reverse reactions are comparable for AOU and OU (30.9 *vs.* 30.6 kcal mol^−1^), suggesting similar kinetic accessibility for bond cleavage. However, AOU exhibits a Δ*G*^0^ of −4.4 kcal mol^−1^, which is less negative than −7.8 kcal mol^−1^ of OU, substantiating its reduced thermodynamic stability and a greater propensity for dissociation.

Altogether, the combined experimental and computational results demonstrate that the β-ketoester substituent primarily modulates the thermodynamic landscape of the reversible reaction, favoring bond dissociation without significantly altering the kinetic barrier for cleavage. This shift in equilibrium increases the concentration of free AOs and isocyanates under reaction conditions. When coupled with vacuum-assisted distillation, this thermodynamic bias facilitates efficient separation and recovery of the dissociated species, thereby accounting for the enhanced recycling efficiency observed in PAOU networks.

## Conclusion

In summary, this work demonstrates that substituent engineering of dynamic covalent bonds enables simultaneous control over intermolecular interactions and bond thermodynamics in recyclable polymers. Incorporation of a β-ketoester substituent into oxime–urethane linkages strengthens hydrogen-bonding interactions that enhance network rigidity, resulting in improved mechanical and thermomechanical properties. At the same time, the substituent significantly shifts the thermodynamic equilibrium toward bond dissociation, as evidenced by a nearly two orders of magnitude reduction in equilibrium constant (69 *vs.* 4944 M^−1^). This thermodynamic bias enables efficient depolymerization at 140 °C, leading to high-yield recovery of acetoacetated oximes (82%) and isocyanates (87%) *via* vacuum distillation—an unprecedented achievement in polyurethane/polyurea chemistry. The dynamic networks further exhibit rapid stress relaxation, excellent reprocessability, and enable closed-loop recycling of carbon fiber-reinforced composites with preserved material integrity. Collectively, these findings establish substituent engineering as an effective strategy to decouple mechanical performance from recyclability by integrating supramolecular interactions and thermodynamic control within a single dynamic covalent bond.

## Author contributions

Q. C. contributed methodology and investigation; J. Z. contributed investigation, software, and methodology; Y. L. and P. Y. contributed formal analysis; M. C., Y. X., and J. G. contributed investigation, testing, and analysis; S. S.: contributed small-molecule synthesis and analysis; Y. M. and Z. L.: contributed funding acquisition, conceptualization, methodology supervision, writing – original draft, and reviewing and editing.

## Conflicts of interest

There are no conflicts to declare.

## Supplementary Material

SC-017-D6SC04529D-s001

## Data Availability

The data supporting this article have been included as part of the supplementary information (SI). Supplementary information: materials, experimental procedures, characterization, theoretical calculations, and Fig. S1–S45. See DOI: https://doi.org/10.1039/d6sc04529d.
