# Supplementary material for: Substituent engineering of dynamic covalent bonds enables simultaneous enhancement of performance and recyclability
Source: Chem Sci. 2026 Jun 22;17(31):15108–16. doi: 10.1039/d6sc04529d (PMC13313018; doi:10.1039/d6sc04529d)
Supplement: Supplementary file 1 [file SC-017-D6SC04529D-s001.pdf]

Supplementary Information (SI) for Chemical Science.
This journal is © The Royal Society of Chemistry 2026

Support Information

for

Substituent Engineering of Dynamic Covalent Bonds Enables Simultaneous Enhancement of Performance and Recyclability

Zhiyong Liu,[1,3]* Qian Chao,[3] Jinyan Zhao,[3] Ying Lin,[3] Ping Yu,[4] Min Chen,[5] Shengyu Shi,[3]* Yixin Xiang,[3] Jiangang Gao,[3] and Youwei Ma[2]*

1 School of Chemistry & Chemical Engineering, Anhui University, Hefei, China;

2 Institute of Materials, École Polytechnique Fédérale de Lausanne (EPFL), CH-1015 Lausanne, Switzerland;

3 School of Chemical and Environmental Engineering, Anhui Polytechnic University, Wuhu, China;

4 School of Environmental and Chemical Engineering, Jiangsu Ocean University, Lianyungang, China;

5 Center for Water and Ecology, State Key Joint Laboratory of Environment Simulation and Pollution Control, School of Environment, Tsinghua University, Beijing, China.

* Correspondance Z. Liu: liuzhiyong@ahpu.edu.cn; S. Shi: shisy@ahpu.edu.cn; Y. Ma: youwei.ma@epfl.ch

Contents

Materials and Instrumentation 3
- Materials 3
- Instrumentation 3

Preparation and properties of polymer networks 4
- Synthesis of Trimethylolpropane acetoacetates (TA) 4
- Synthesis of Trimethylolpropane acetoacetates oxime (TAO) 4
- Synthesis of trimethylolpropane oxime (TO) 5
- Preparation of cross-linked polymer networks (PAOU) 5
- Preparation of cross-linked normal poly(oxime-urethane)s 5

Preparation and recycling of composites 6

Synthesis of small molecule compounds 14
- Synthesis of ethyl (Z)-2-(hydroxyimino)-3-oxobutanoate (AO) 14
- Synthesis of ethyl (Z)-2-(((butylcarbamoyl)oxy)imino)-3-oxobutanoate (AOU1) 14
- Synthesis of ethyl (Z)-3-oxo-2-(((phenethylcarbamoyl)oxy)imino)butanoate (AOU2) 15
- Synthesis of benzyl 3-oxobutanoate 15
- Synthesis of benzyl (Z)-2-(hydroxyimino)-3-oxobutanoate (AO2) 15
- Synthesis of benzyl (Z)-2-(((butylcarbamoyl)oxy)imino)-3-oxobutanoate (AOU3) 16
- Synthesis of benzyl (Z)-3-oxo-2-(((phenethylcarbamoyl)oxy)imino)butanoate (AOU4) 16
- Synthesis of (E)-butan-2-one O-butylcarbamoyl oxime (OU1) 17
- Synthesis of (E)-butan-2-one O-phenethylcarbamoyl oxime (OU2) 17

Variable-temperature NMR experiments 18

Computational details 24

References 28

Appendix of NMR spectra 30

Appendix of the Cartesian coordinates (Å) in THF 42

Materials and Instrumentation

Materials

Trimethylolpropane, ethyl acetoacetate, benzyl alcohol, *tert*-butyl acetoacetate, hydroxylamine hydrochloride, 2-butanone oxime (**BO**), *n*-butyl isocyanate (**1**), phenethyl isocyanate (**2**), hexamethylene diisocyanate (**HDI**), trimethyl hexamethylene diisocyanate (**TMDI**), 4,4-methylenedicyclohexyl diisocyanate (**HMDI)** were purchased from Adamas-beta (Shanghai, China). Acetic acid and sodium nitrite were purchased for Sinopharm Chemical Reagent Co., Ltd (Beijing, China). The solvents and materials were used as received.

Instrumentation

Nuclear magnetic resonance (NMR) spectroscopy was carried out at 297.2 K on a Bruker Avance NEO 500M spectrometer at frequencies of 500 MHz for ^{1}H nuclei and 126 MHz for ^{13}C nuclei. Spectra were calibrated to the residual solvent peak of $CDCl_3$ (7.26 ppm ^{1}H NMR; 77.16 ppm ^{13}C NMR) and DMSO-d_6 (2.5 ppm ^{1}H NMR; 128.06 ppm ^{13}C NMR).

Differential scanning calorimetry (DSC) measurements were performed on DSC 25 (TA Instruments, USA) under nitrogen atmosphere at a heating/cooling rate of 20 °C min^{-1} in the temperature range of −20–100 °C.

Dynamic mechanical analyses (DMA) were performed on the TA Instruments Model Q850 DMA in tension mode. The temperature ranged from −80 to 110 °C with a heating rate of 3 °C min^{-1}, a frequency of 1 Hz, and an amplitude of 0.2 % strain. Stress relaxation analyses were carried out on the Q850 DMA machine in a tension mode as well. A rectangular sample was first kept isothermally at a specific temperature for 3 min before a constant 5% strain was applied to the sample, and the stress was recorded at the same time.

Tensile testing measurements were carried out at 5 mm min^{-1} on AI-7000-MU1 universal testing machine (Gotech testing machines, Dong Guan, China). Uniaxial tensile tests were carried out using dumbbell-shaped specimens (length: 50 mm; central width: 4 mm) that were cut from compression molded films according to GB/T 528. At least five specimens were tested for each sample.

Solubility experiments were conducted as follows. The weighed samples (m_0) were immersed in different solvents, including 1,4-dioxane, toluene, tetrahydrofuran (THF), chloroform ($CDCl_3$) at room temperature for 7 days. The solvent was changed every day. The solvent was carefully removed by a syringe, and the surface was wiped with a tissue after swelling, after which the samples were weighed (m_1). The swelling

samples were dried under vacuum at 80 °C for 48 h. Then the samples were weighed again (m_2). The gel fraction (GF) was defined as $m_2/ m_0 \times 100\%$. The swelling ratio (SR) was defined as $(m_1 - m_2)/m_2 \times 100\%$.

High-performance liquid chromatography (HPLC) was performed on an LC 4000 system (Jasco, Japan) under the following conditions: a Supersil ODS2-C18 column (5 μm particle size, 4.6 mm × 250 mm) was used; the column temperature was maintained at 25 °C; the eluent phase consisted of water and acetonitrile in a volume ratio of 4:6; the flow rate was set at 1 mL/min; and the detection wavelength was 254 nm.

Temperature-variable **Fourier Transform Infrared Spectroscopy (FTIR)** was carried out on a Nicolet iS20 FTIR spectromerter (ThermoFisher, US) with a heating cell from 70 to 120 °C at a heating rate of 5 °C min^{-1}.

Preparation and properties of polymer networks

Synthesis of Trimethylolpropane acetoacetates (TA)

The synthesis of **TA** adopted the procedures shown as follows. Trimethylolpropane (10.06 g, 0.075 mol) and *tert*-butyl acetoacetate (53.40 g, 0.338 mol) were mixed in a flask equipped with a condenser and a Dean-Stark apparatus. The mixture was heated at 140 °C for 3 h and cooled down to room temperature. The resulting reaction solution was poured into a large amount of petroleum ether for precipitation. The precipitate was washed three times, and then subjected to vacuum drying to obtain the final product **TA** (26.67 g, yield: 92%). ^{1}H NMR (500 MHz, DMSO-d_6) δ 4.01 (s, 6H), 3.63 (s, 6H), 2.18 (s, 9H), 1.40 (q, J = 7.5 Hz, 2H), 0.83 (t, J = 7.6 Hz, 3H). ^{13}C NMR (126 MHz, DMSO-d_6) δ 201.87, 167.44, 64.76, 49.93, 40.97, 30.45, 22.69, 7.54.

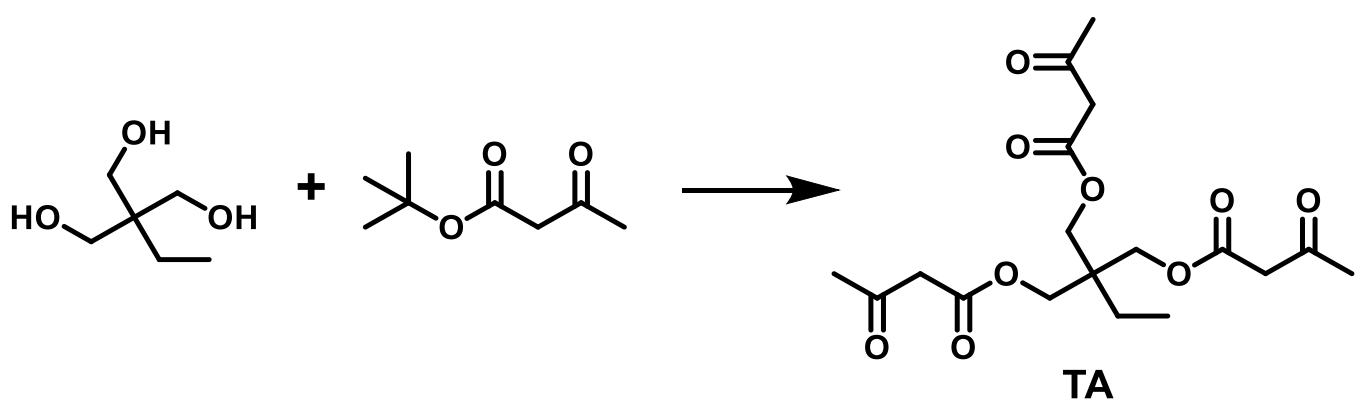

Synthesis of Trimethylolpropane acetoacetates oxime (TAO)

TA (10.05 g, 0.026 mol) and acetic acid (9.36 g, 0.156 mol) were first mixed in a flask at –5 °C, then a 30 wt% aqueous solution of sodium nitrite (10.76 g, 0.156 mol) was added dropwise. After stirred overnight at –5 °C, the mixture was extracted with ethyl acetate (3×200 mL). The combined organic phase was washed three times with saturated sodium bicarbonate solution, dried with anhydrous sodium sulfate and filtered. Removal of solvent using rotary evaporation and vacuum drying afforded product **TAO** (10.95 g, yield: 89%). ^{1}H NMR (500 MHz, DMSO-d_6) δ 13.50 – 13.22 (m, 3H), 4.17 (d, J = 26.4 Hz, 6H), 2.36 (s, 9H), 1.52 – 1.12

(m, 2H), 0.91 – 0.72 (m, 3H). ^{13}C NMR (126 MHz, DMSO-d_6) δ 194.06, 161.90, 150.72, 63.70, 41.84, 25.57, 22.31, 6.72.

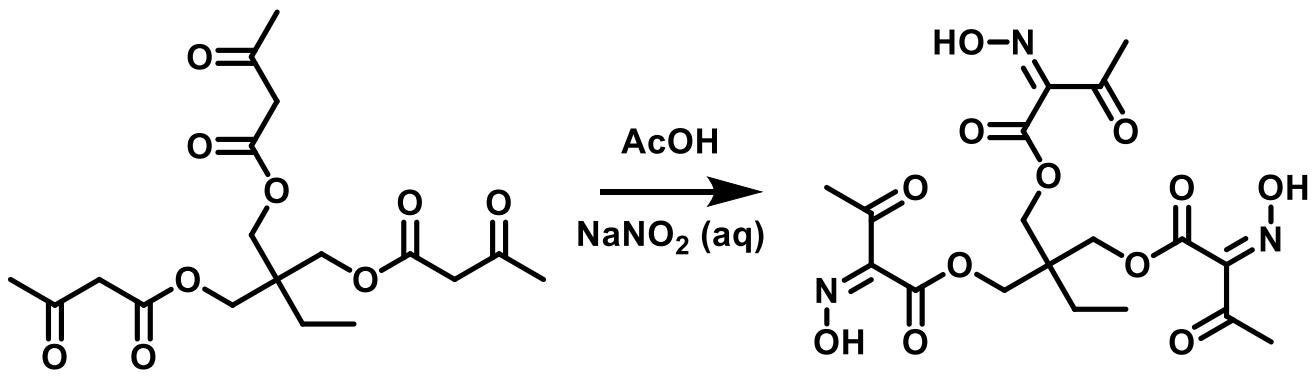

TA TAO

Synthesis of trimethylolpropane oxime (TO)

TA (10.05 g, 0.026 mol) was dissolved in pyridine (19.98 g, 0.253 mol). Following the addition of hydroxylamine hydrochloride (6.53 g, 0.094 mol), the reaction mixture was stirred at room temperature for 24 h. The pyridine was subsequently removed using rotary evaporation, and the mixture was extracted with ethyl acetate (3×200 mL). The combined organic phase was washed three times with saturated sodium bicarbonate solution, dried with saturated sodium chloride and filtered. Removal of solvent using rotary evaporation and vacuum drying afforded the product **TO** (10.08 g, yield: 90%). ^{1}H NMR (500 MHz, DMSO-d_6) δ 10.65 (dd, J = 26.9, 8.9 Hz, 3H), 4.09 – 3.85 (m, 6H), 3.34 – 3.15 (m, 6H), 1.81 (dd, J = 31.7, 1.9 Hz, 9H), 1.45 – 1.32 (m, 2H), 0.86 – 0.76 (m, 3H). ^{13}C NMR (126 MHz, DMSO-d_6) δ 169.99, 168.92, 150.24, 149.01, 64.20, 41.03, 34.43, 20.69, 14.14, 7.64.

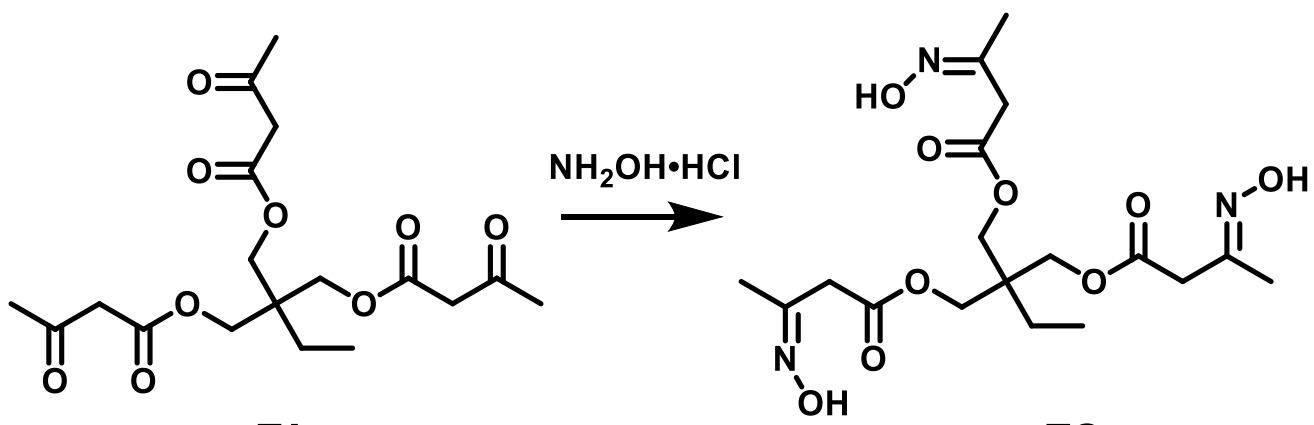

TA TO

Preparation of cross-linked polymer networks (PAOU)

TAO (3.00 g, 0.006 mol) and 0.018 mol diisocyanate were dissolved in 40 mL THF, then the mixture was casted into a polytetrafluoroethylene mold (8 cm × 8 cm ×1 cm, $l \times w \times h$). The mold was sent to a drying oven and gradually heated to 40 °C. A solid film was obtained after 48 h. Three films were prepared and denoted as **PAOU-HDI**, **PAOU-TMDI** and **PAOU-HMDI** respectively. The film names were denoted based on the type of diisocyanates used in the preparation. For example, **PAOU-HDI** film represents that HDI is used as the diisocyanate.

Preparation of cross-linked normal poly(oxime-urethane)s

For comparison, **TO** was also used to prepare a cross-linked network using similar procedures. In brief, **TO**

(3.00 g, 0.007 mol) and HDI (3.53 g, 0.021 mol) were dissolved in 40 mL THF, then the mixture was casted into a polytetrafluoroethylene mold (8 cm × 8 cm ×1 cm, *l* × *w* × *h*). The mold was sent to a drying oven and gradually heated to 40 °C. A solid film was obtained after 48 h.

Thermal reprocessing experiments were conducted on a press vulcanizer (SNA, Wuxi, China). Take **PAOU-HDI** as an example, the film was first cut into small pieces, and then the pieces were piled up in a steel mould with a dimension of 5 cm × 5 cm × 0.1 cm (*l* × *w* × *h*). The mould was comperssed under 10 MPa at 100 °C for 0.5 h. The reprocessed film was tested to evaluate the recycling efficency.

Closed-loop recycling of the raw material from **PAOU-HDI** via vacuum distillation is briefly described as follows. **PAOU-HDI** film (3.16 g) was added in a reaction flask, which was heated at 140 °C under a vacuum of 0.01 Pa for 3 h. The distilled liquid was collected in a receiving flask attached to the end of condenser. The weight of the distillate in the receiving flask was 0.95 g (yield: 87%), which was identified as HDI (one of the raw materials) via ^{1}H NMR spectroscopy. Ethyl acetate (10 mL) was added to the reaction flask, followed by filtration. The filtrate was then concentrated by rotary evaporation, and finally purified by column chromatography to obtain 1.69 g (yield: 82%) of the product, which was also confirmed to be **TAO** (another raw material) through ^{1}H NMR spectroscopy. The re-synthesized **PAOU-HDI** could be prepared using the two recovered materials. Using the normal poly(oxime-urethane)s (3.20 g) as the control sample, the same recovery method was adopted to retrieve the starting materials, yielding 0.16 g (yield: 14%) of HDI and 0.22 g (yield: 11%) of **TO** after treatment.

Preparation and recycling of composites

TAO (3.00 g, 0.006 mol) and HDI (3.02 g, 0.018 mol) were dissolved in 40 mL THF. A carbon fiber cloth (7 cm × 7 cm, *l* × *w*) was placed inside a polytetrafluoroethylene mold (8 cm × 8 cm ×1 cm, *l* × *w* × *h*), and the mixture was casted into the mold, completely impregnating and submerging the carbon fiber cloth. The mold was sent to a drying oven and gradually heated to 40 °C. A solid composite film was obtained after 48 h.

The closed-loop recycling of the composite is similar to that of **PAOU-HDI**, with the difference that, after heating, ethyl acetate is added to the reaction flask to wash the carbon fiber cloth. The resulting filtrate is then concentrated and purified by column chromatography to recover the starting material **TAO**. The carbon fiber cloth, after multiple washes, is dried to remove any residual solvent.

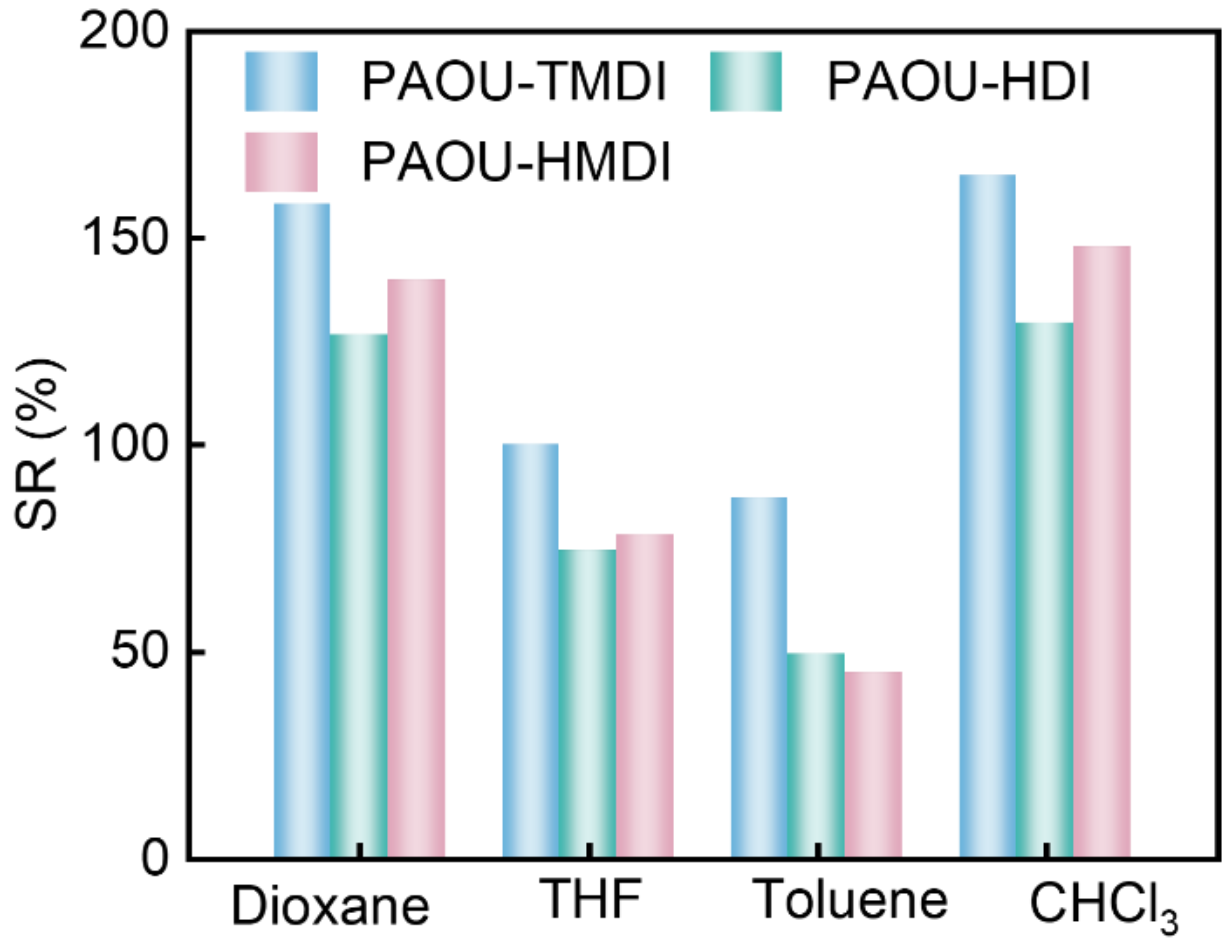

Figure S1. The swelling ratio (SR) of **PAOU-TMDI**, **PAOU-HDI** and **PAOU-HMDI** after being immersed in different solvents at room temperature for 7 days.

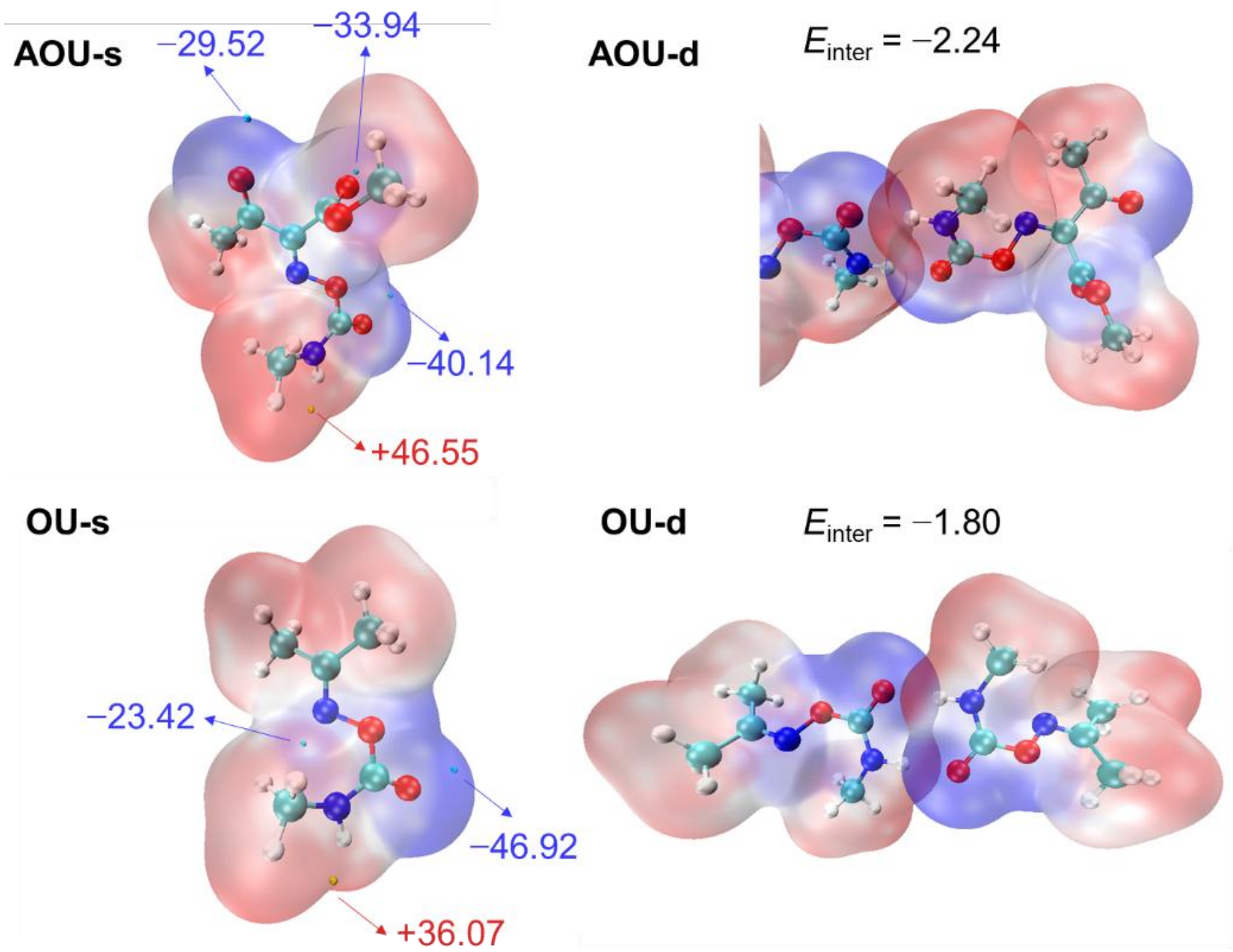

Figure S2. Electrostatic potential on the molecular surface of single-molecule **AOU** (AOU-s) and **OU** (OU-s) (Left), and the intermolecular interaction of the dimer AOU-d and OU-d (right). The unit of electrostatic potential is kcal/mol, and intermolecular interaction energies (E_{inter}) of AOU-d and OU-d are −2.24 and −1.8 kcal/mol, respectively.

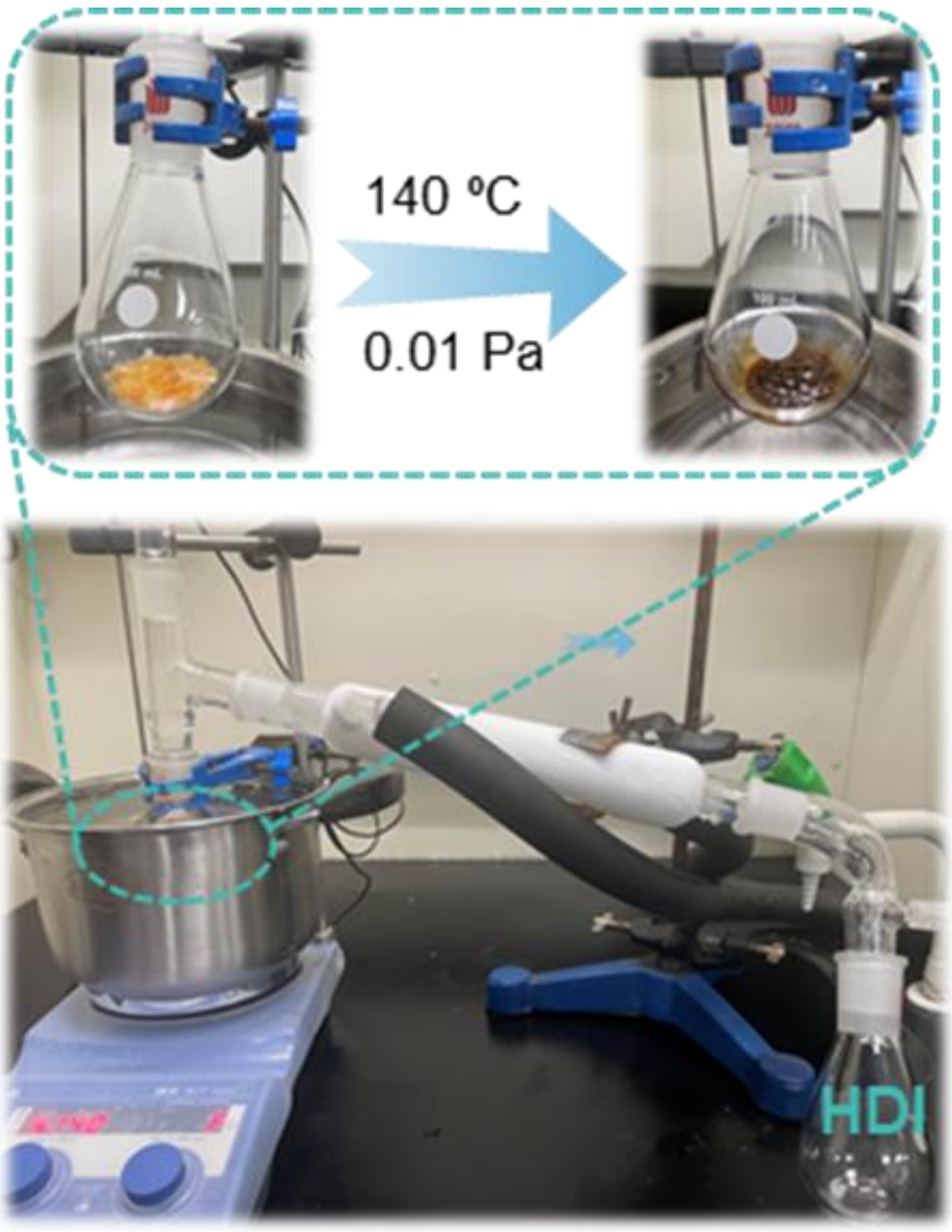

Figure S3. Photographs showing the vacuum distillation set-up for the separation of HDI and **TAO** from **PAOU-HDI**.

Table S1. Summary of representative state-of-the-art systems on the thermal reprocessing and chemical recycling of polyurethane/polyurea and their analogues.

Name	Structure	Catalyst	Reprocessing	Chemical recycling	Isocyanate recovery	Reference
Hindered urea	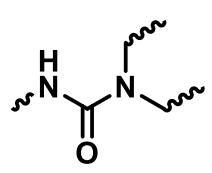	no	100 ℃, 300 kPa, 20 min	3 equiv. of 2-(tert-butylamino)ethanol, 60 ℃, 12 h	no	Cheng et al. [1]
		no	80 ℃, 3 MPa, 60 min	3 equiv. of *N,N'*-di-tert-butyl-p-xylylenediamine, 60 ℃, 4 h	no	Cheng et al.[2]
			130 ℃, 4 MPa, 5 min	3 equiv. of 2-(*t*-butylamino)ethyl methacrylate, 70 ℃, 4 h	no	Oh et al.[3]
Phenol–urethane	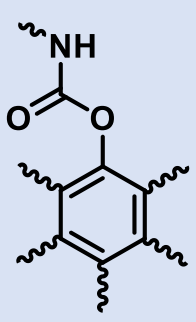	*p*-TsOH	130 °C, 25 MPa, 35 min	/	no	Zhang et al.[4]
		DABCO	90–140 °C, 5 MPa, 5 min	/	no	Xu et al. [5]
		DBTDL	150–230 °C , 1 MPa, 3	Excess 1,4 butanediol,	no	Caillol and

			h	180 °C, 5h		Bakkali-Hassani et al. [6]
Acylsemicarbazide	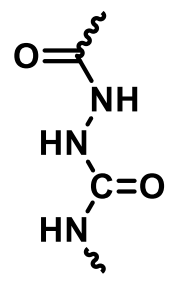	no	130 °C, 20 MPa, 1 h	DMSO, 110 °C, 1.5 h	no	Xia and Wang et al. [7]
		no	140 °C, 15 MPa, 1 h	DMF, 120 °C, 3 h	no	Xia and Zuilhof et al. [8]
		DBTDL	100 °C, 30 min	DMF 120 °C, 3 h	no	Li and Jiang et al. [9]
Pyrazole–urea	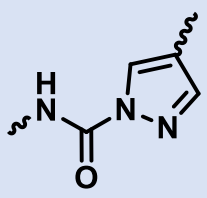	no	130 °C, 10 MPa, 30 min	1 equiv. of bifunctional pyrazole, 110 °C, 1 h	no	Xu, Zhao, and Zhang et al. [10]
		no	130 °C, 20 min	2 M HCl/EtOH, 70 °C, 36 h	no	Gao and Zhao et al. [11]
Oxime-urethane	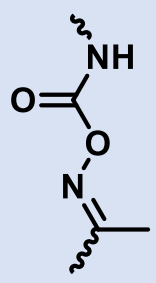	no	120 °C, 10 MPa, 0.5 h	acetophenone oxime, 90 ℃, 9 h	no	Xu and Zhao et al.[12]
		no	120–140 °C, 5 MPa, 10 min	/	no	You et al. [13]
		no	110 °C, 10 MPa, 30 min	DMF, 100 °C, 5 h	no	Xia et al. [14]
Enamide derivatives	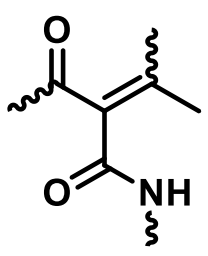	no	120 °C, 4 tons, 30 min	10-fold by weight of acetylacetone, 140 °C, 15 h	no	Shi, Berrocal, and Weder et al. [15]
		no	160 °C, 30 MPa, 30 min	/	no	Shi et al. [16]
		no	120 °C, 10 MPa, 40 min	/	no	Shi and Ma et al. [17]
Normal urethane	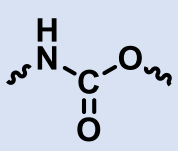	no	/	20-fold by weight of acetoxime, 130 °C, 20 min	no	Xie and Zhu et al.[18]
		TBD	/	DMF, 100 °C, 60 min	no	Xie et al. [19]
		no	/	Itaconic acid, 180 °C, 180 min	no	Xie and Fang et al. [20]
		triethylamine	/	β-chlorocatecholborane, 50–150 °C	< 23%	Epps III and Korley et al. [21]
**AOU**	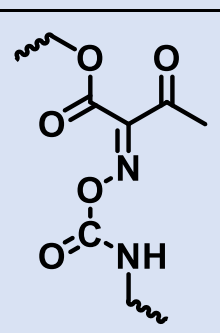	**no**	**100 °C, 10 MPa, 30 min**	**Vacuum distillation, 140 °C, 0.01 Pa**	**87%**	**This work**

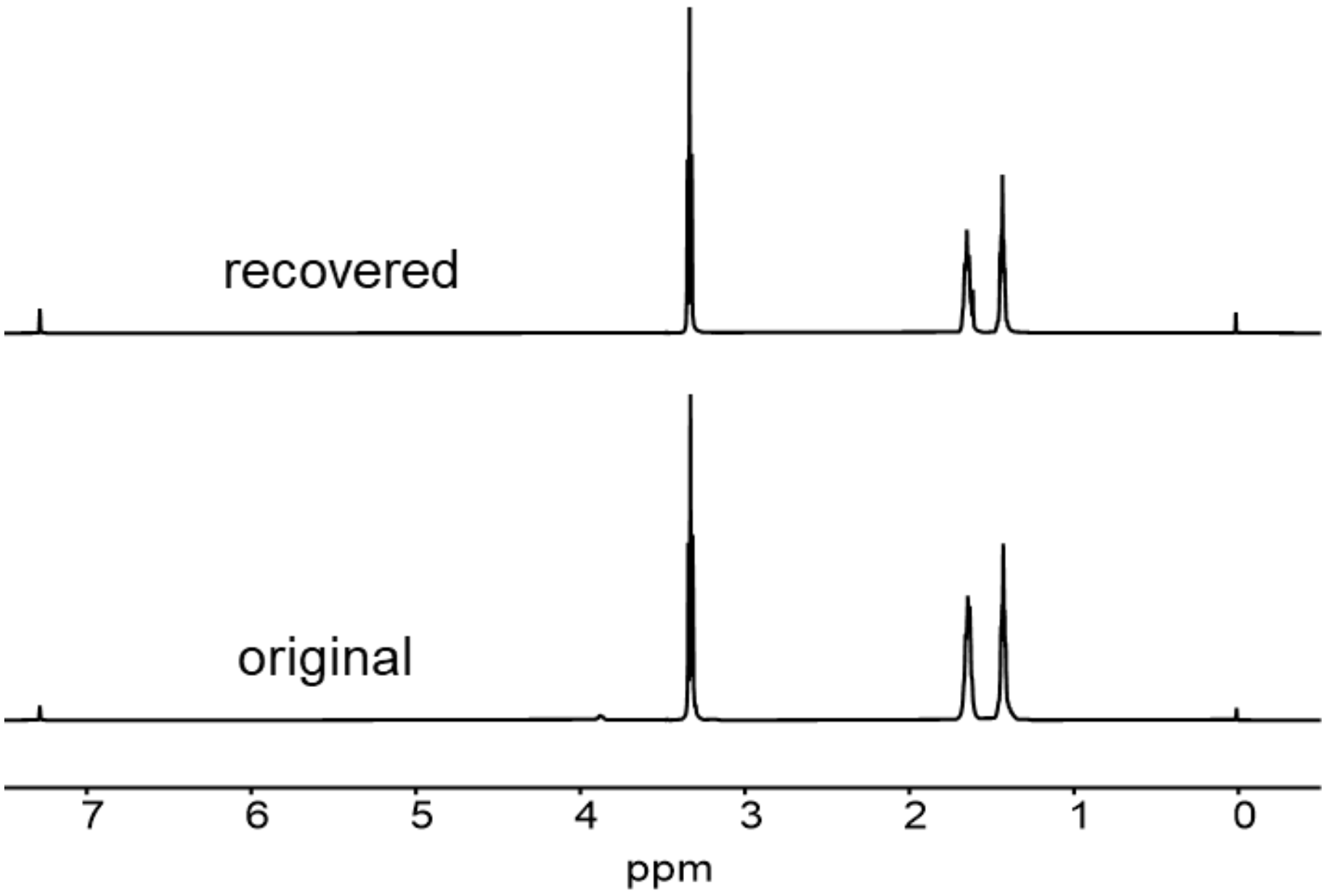

Figure S4. Comparison between the ^{1}H NMR spectra ($CDCl_3$, 400 MHz) of the original and recovered HDI from composite.

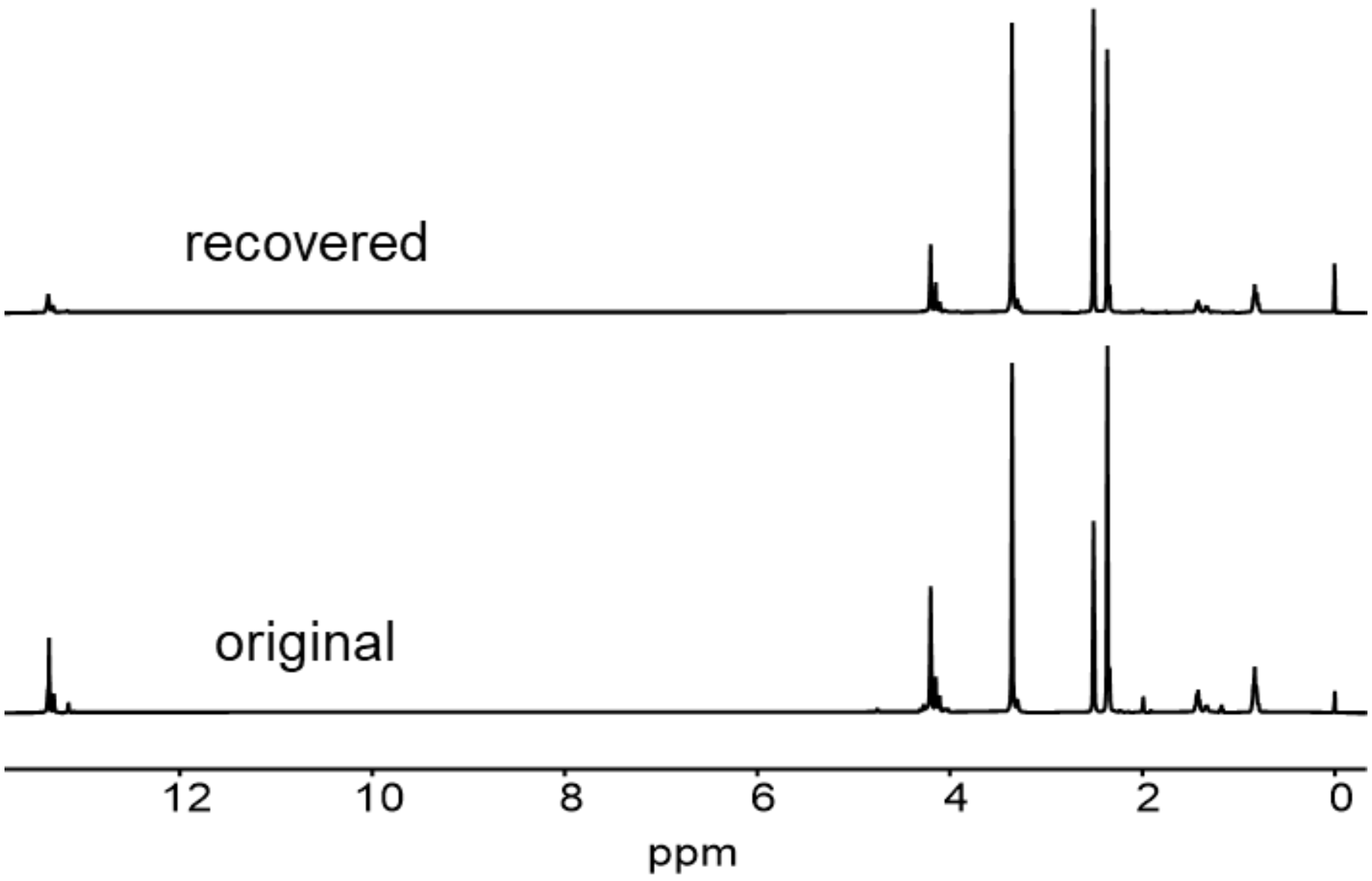

Figure S5. Comparison between the ^{1}H NMR spectra ($CDCl_3$, 400 MHz) of the original and recovered **TAO** from composite.

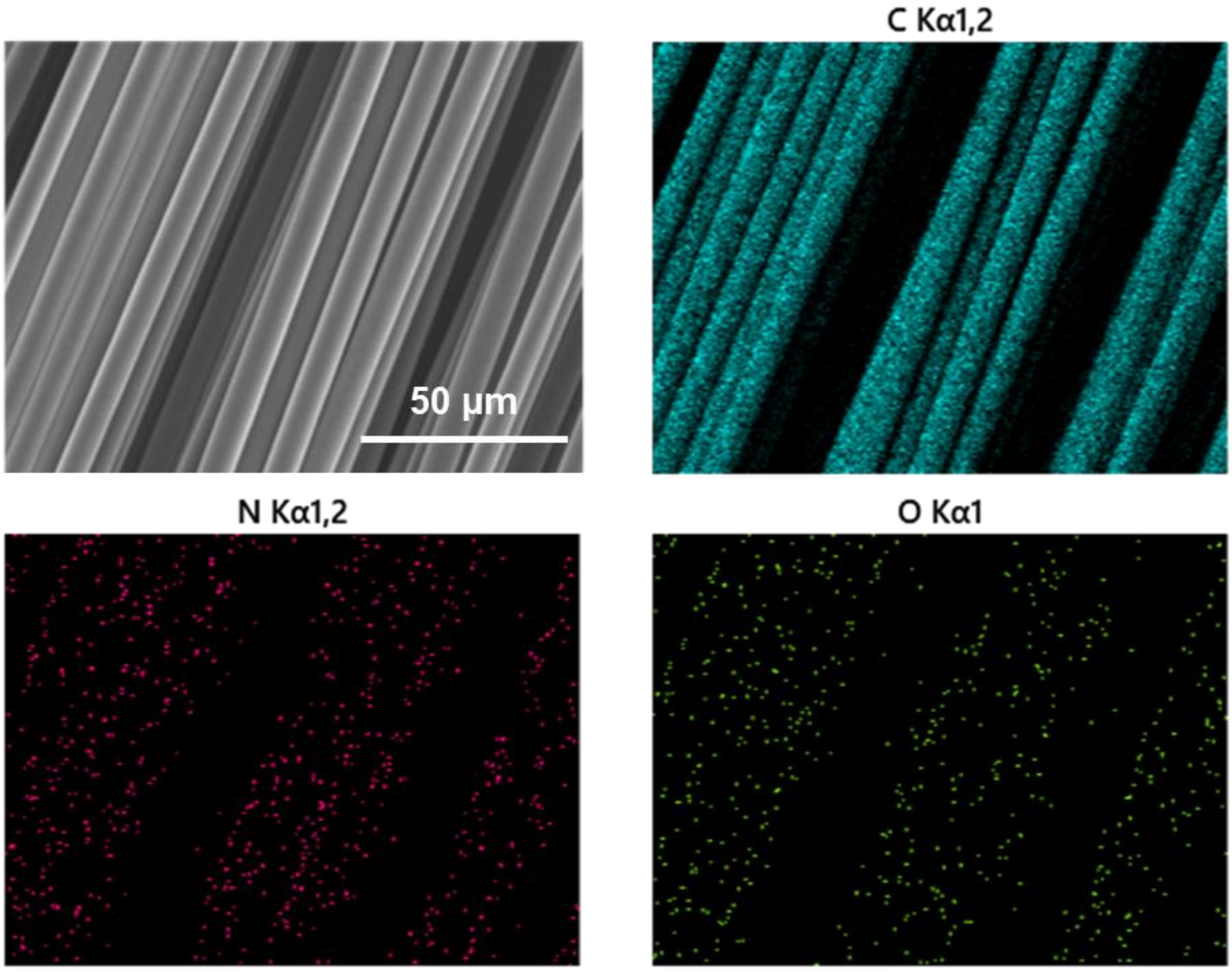

Figure S6. SEM and EDX images of the original CFs.

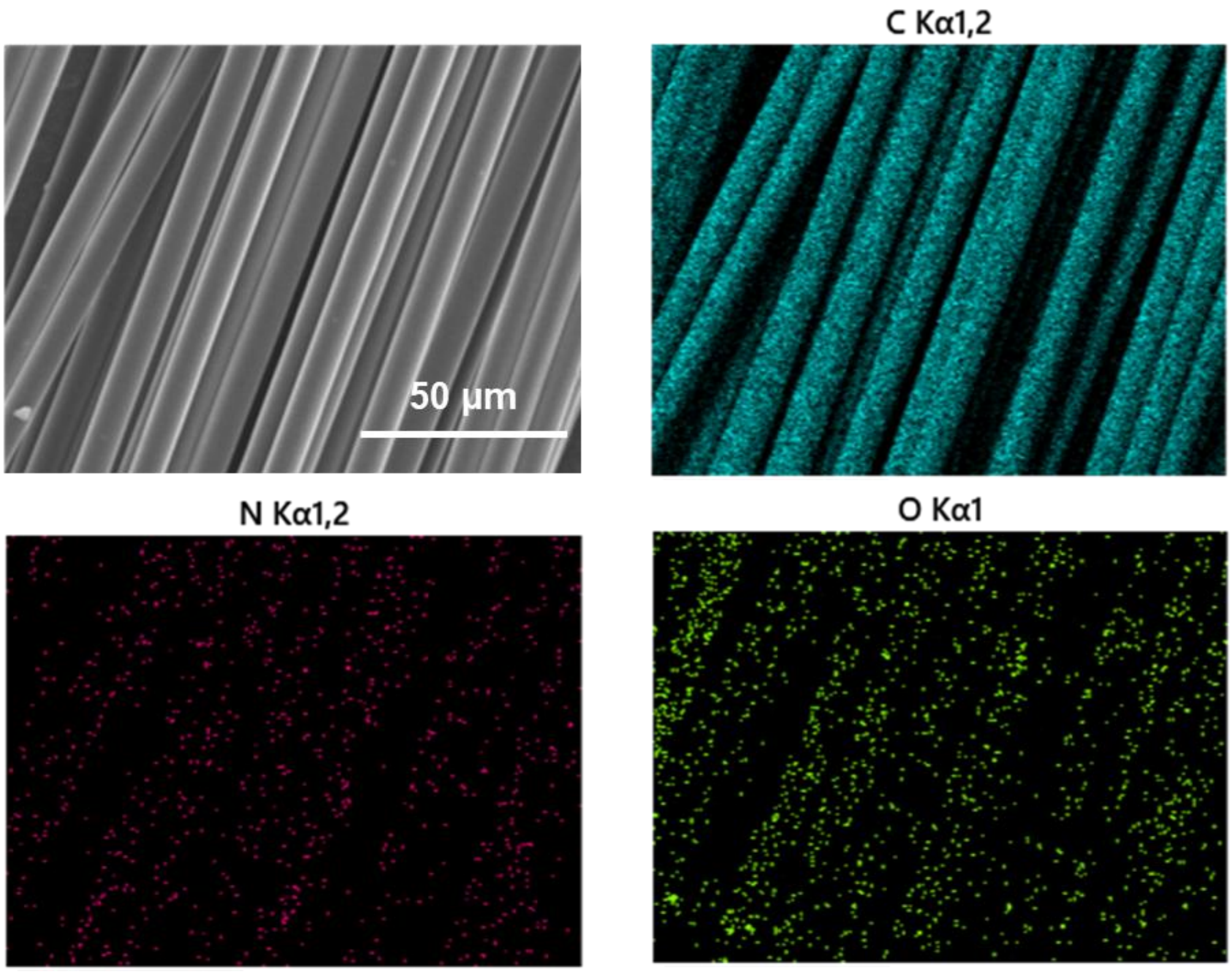

Figure S7. SEM and EDX images of the recovered CFs.

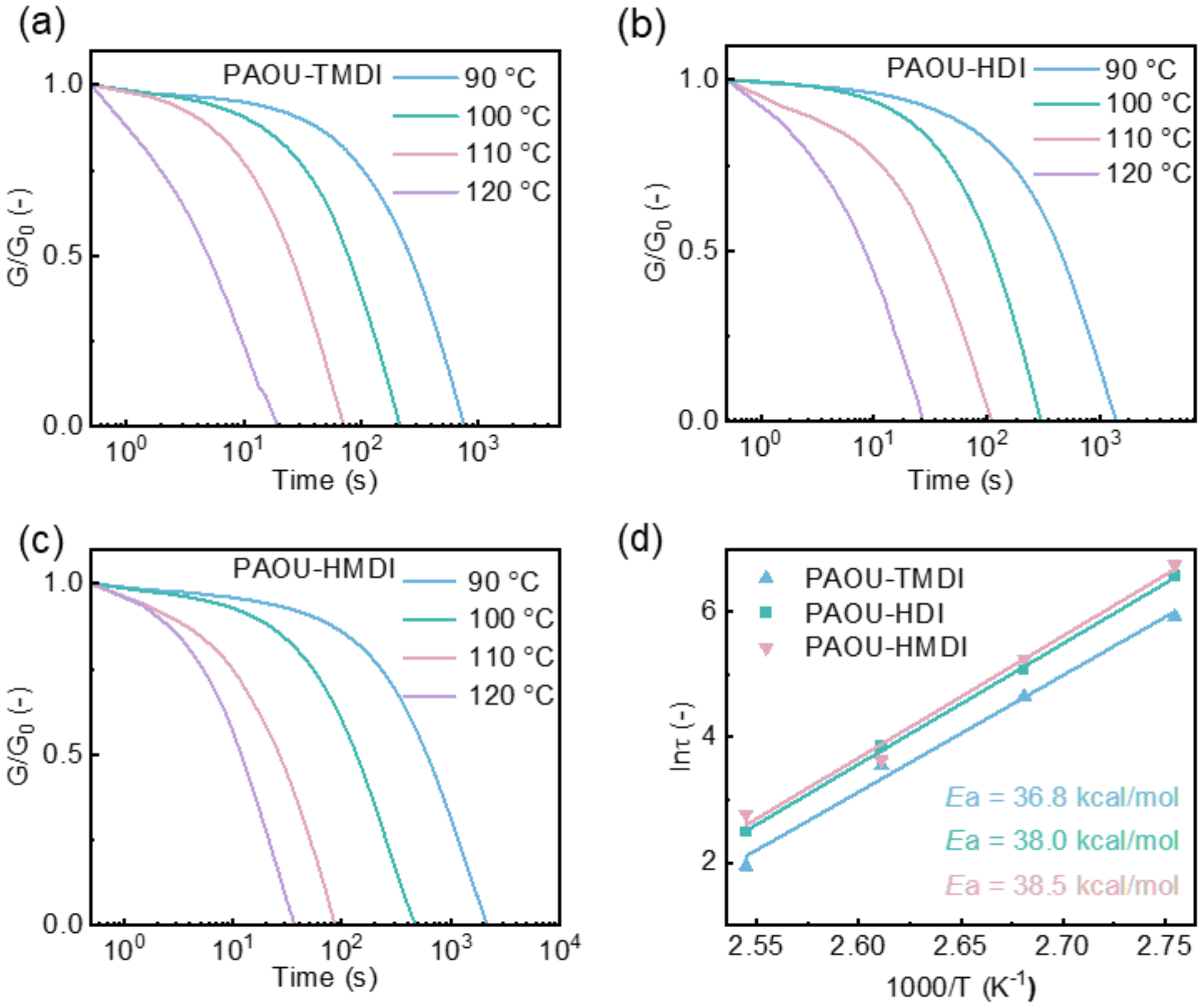

Figure S8. Stress relaxation curves of (a) **PAOU-TMDI**, (d) **PAOU-HDI**, and (c) **PAOU-HMDI**. (d) Arrhenius plots for the stress relaxation of these **PAOU**s.

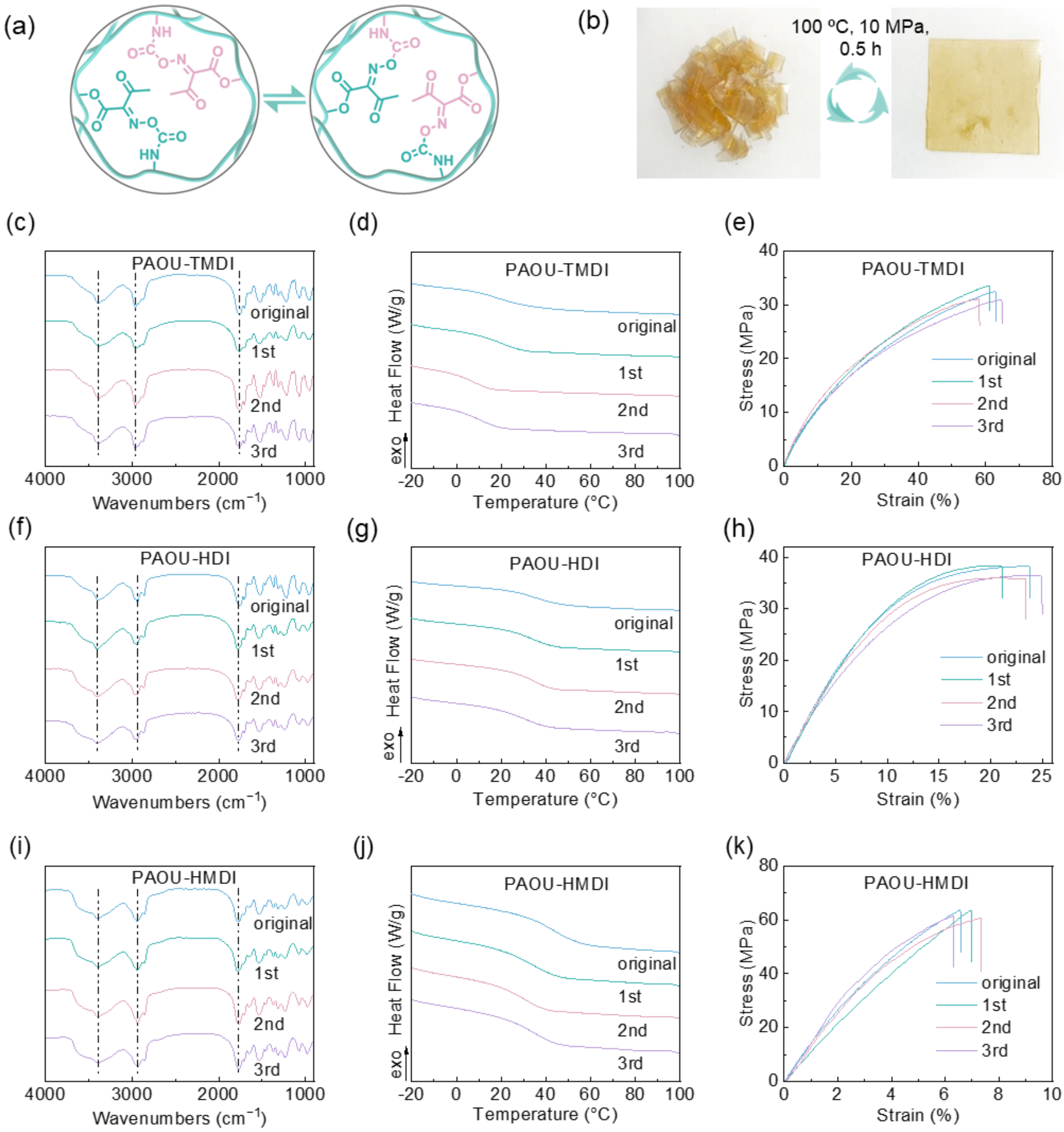

Figure S9. (a) Exchange reaction between two **AOU** linkages in the **PAOU** networks. (b) Photographs showing the reprocessing of **PAOU-HDI** from small pieces of material (left) into a new film (right) by compression molding. (c, f, i) FTIR spectra, (d, g, j) DSC traces and (e, h, k) stress–strain curves of the original and thermally reprocessed (c–e) **PAOU-TMDI**, (f–h) **PAOU-HDI**, and (i–k) **PAOU-HMDI**.

Synthesis of small molecule compounds

Synthesis of ethyl (Z)-2-(hydroxyimino)-3-oxobutanoate (AO)

Ethyl acetoacetate (5.00 g, 0.038 mol) and acetic acid (9.88 g, 0.076 mol) were added into a flask equipped with a magnetic stir bar. The mixture was stirred at –5 °C. Subsequently, a 30 wt% aqueous solution of sodium nitrite (5.25 g, 0.076 mol) was added dropwise into the mixture. The reaction proceeded at –5 °C overnight. Then the mixture was extracted with ethyl acetate (3×100 mL). The combined organic phase was washed three times with saturated sodium bicarbonate solution, dried with anhydrous sodium sulfate and filtered. Removal of solvent using rotary evaporation and vacuum drying afforded product **AO** (5.09 g, yield: 84%). ^{1}H NMR (500 MHz, DMSO-d_6) δ 13.27 (d, J = 1.4 Hz, 1H), 4.30 – 4.19 (m, 2H), 2.35 (d, J = 1.3 Hz, 2H), 1.23 (td, J = 7.1, 1.2 Hz, 3H). ^{13}C NMR (126 MHz, DMSO-d_6) δ 194.24, 162.29, 151.18, 62.02, 25.56, 14.39.

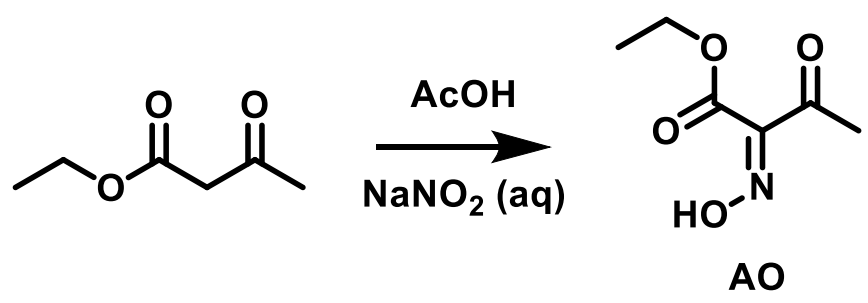

Synthesis of ethyl (Z)-2-(((butylcarbamoyl)oxy)imino)-3-oxobutanoate (AOU1)

n-Butyl isocyanate **1** (2.28 g, 0.023 mol) was dissolved in anhydrous ether (20 mL) in a flask equipped with a magnetic stir bar. A solution of **AO** (3.02 g, 0.019 mol) in anhydrous ether (30 mL) was then added dropwise. The reaction progress was monitored by thin-layer chromatography (TLC). Upon completion, the reaction mixture was concentrated to approximately 10 mL and poured into petroleum ether (100 mL), precipitating a crude product. The crude product was washed repeatedly with petroleum ether and further purified by column chromatography using petroleum ether/ethyl acetate (3:1, v/v) as the eluent to afford product **AOU1** (3.90 g, yield: 80%). ^{1}H NMR (500 MHz, DMSO-d_6) δ 7.98 (t, J = 5.9 Hz, 1H), 4.43 – 4.21 (m, 2H), 3.11 (q, J = 6.7 Hz, 2H), 2.48 (d, J = 1.2 Hz, 3H), 1.47 (p, J = 7.2 Hz, 2H), 1.39 – 1.20 (m, 5H), 0.96 – 0.81 (m, 3H). ^{13}C NMR (126 MHz, DMSO-d_6) δ 193.45, 160.34, 152.60, 152.11, 62.80, 40.84, 31.53, 25.96, 19.78, 14.25, 13.93.

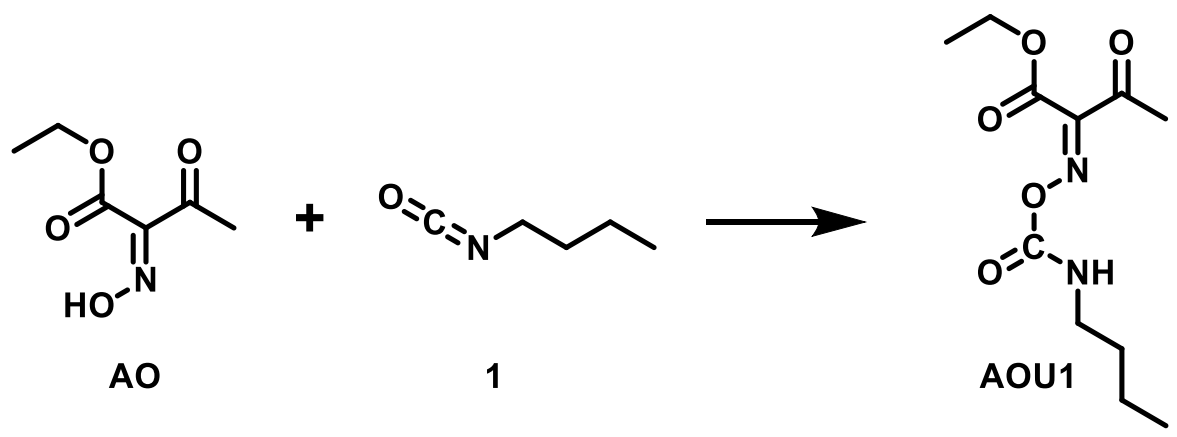

Synthesis of ethyl (Z)-3-oxo-2-(((phenethylcarbamoyl)oxy)imino)butanoate (AOU2)

Phenethyl isocyanate **2** (3.38 g, 0.023 mol) was dissolved in anhydrous ether (30 mL) in a flask equipped with a magnetic stir bar. A solution of **AO** (3.02 g, 0.019 mol) in anhydrous ether (30 mL) was then added dropwise. The reaction progress was monitored by TLC. Upon completion, the reaction mixture was concentrated to approximately 10 mL and poured into petroleum ether (100 mL), precipitating a crude product. The crude product was washed repeatedly with petroleum ether and further purified by column chromatography using petroleum ether/ethyl acetate (4:1, v/v) as the eluent to afford **AOU2** (4.83 g, yield: 83%). ^{1}H NMR (500 MHz, DMSO-d_6) δ 8.10 (t, J = 5.7 Hz, 1H), 7.37 – 7.15 (m, 5H), 4.35 (q, J = 7.2 Hz, 2H), 3.43 – 3.31 (m, 2H), 2.82 (t, J = 7.4 Hz, 2H), 2.48 (s, 3H), 1.26 (dt, J = 22.0, 7.1 Hz, 3H). ^{13}C NMR (126 MHz, DMSO-d_6) δ 193.44, 160.33, 152.61, 152.20, 129.11, 128.82, 126.69, 62.87, 42.69, 35.40, 25.99, 14.29.

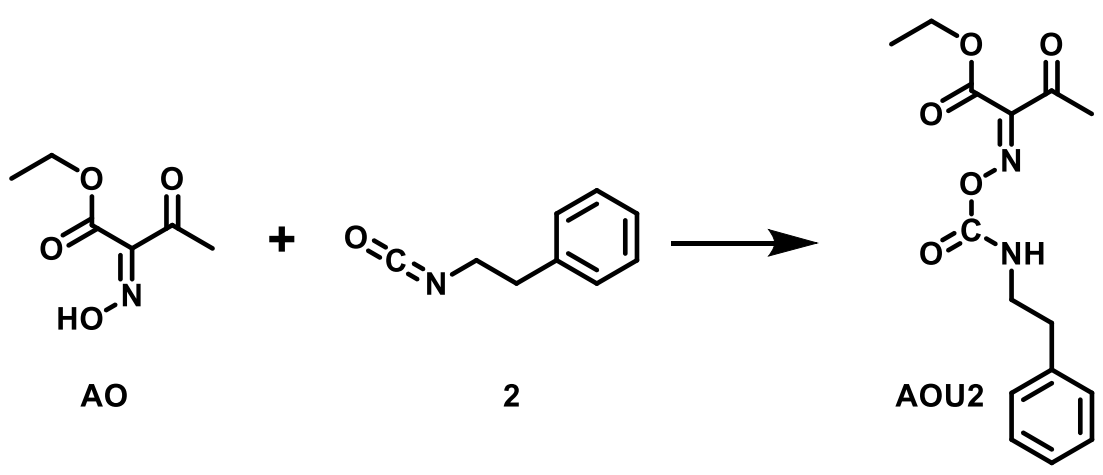

Synthesis of benzyl 3-oxobutanoate

According to our previous works,[16] a brief synthesis procedure was depicted as follows. Benzyl alcohol (5.00 g, 0.046 mol) and *tert*-butyl acetoacetate (10.97 g, 0.069 mol) were mixed in a flask equipped with a condenser and a Dean-Stark apparatus. The mixture was heated at 140 °C for 3 h and cooled down to room temperature. The resulting reaction solution was poured into a large amount of petroleum ether for precipitation. The precipitate was washed three times, and then subjected to vacuum drying to obtain the final product benzyl 3-oxobutanoate (7.82 g, yield: 88%). ^{1}H NMR (500 MHz, DMSO-d_6) δ 7.43 – 7.31 (m, 5H), 5.14 (s, 2H), 3.69 (s, 2H), 2.18 (s, 3H). ^{13}C NMR (126 MHz, DMSO-d_6) δ 201.31, 166.80, 135.60, 128.89, 128.56, 128.49, 66.47, 49.98, 30.52.

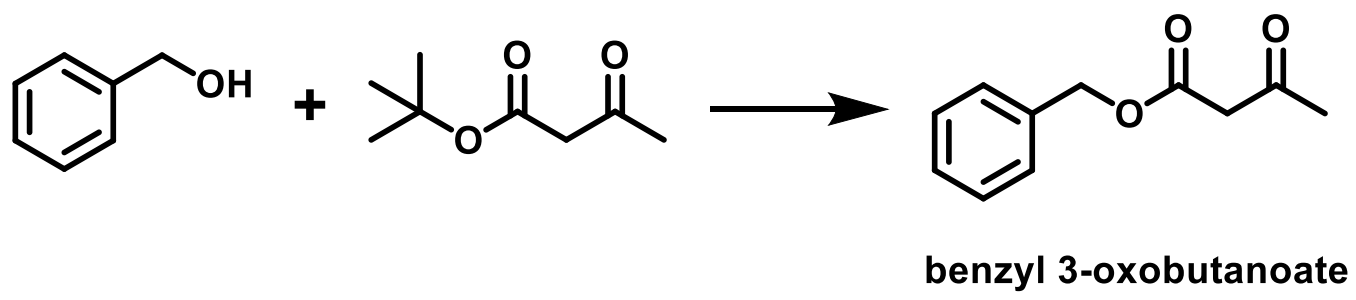

Synthesis of benzyl (Z)-2-(hydroxyimino)-3-oxobutanoate (AO2)

Benzyl 3-oxobutanoate (5.00 g, 0.026 mol) and acetic acid (3.12 g, 0.052 mol) were added into a flask equipped with a magnetic stir bar and stirred at –5 °C. Subsequently, a 30 wt% aqueous solution of sodium

nitrite (3.59 g, 0.052 mol) was added dropwise into the reaction flask. The reaction was allowed to proceed at –5 °C overnight. Then the mixture was extracted with ethyl acetate (3×100 mL). The combined organic phase was washed three times with saturated sodium bicarbonate solution, dried with anhydrous sodium sulfate and filtered. Removal of solvent using rotary evaporation and vacuum drying afforded product **AO2** (4.53 g, yield: 79%). ^{1}H NMR (500 MHz, DMSO-d_6) δ 13.38 (s, 1H), 7.45 – 7.31 (m, 5H), 5.31 (s, 2H), 2.37 (s, 3H). ^{13}C NMR (126 MHz, DMSO-d_6) δ 194.23, 162.25, 150.92, 135.62, 128.91, 128.73, 128.46, 67.08, 25.58.

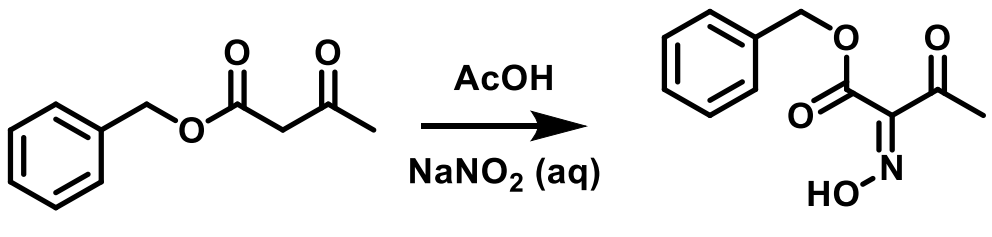

Synthesis of benzyl (Z)-2-(((butylcarbamoyl)oxy)imino)-3-oxobutanoate (AOU3)

n-Butyl isocyanate **1** (1.68 g, 0.017 mol) was were dissolved in toluene (20 mL) in a flask equipped with a magnetic stir bar. A solution of **AO2** (3.09 g, 0.014 mol) in toluene (30 mL) was then added dropwise. The reaction progress was monitored by TLC. Upon completion, the reaction mixture was concentrated to approximately 10 mL and poured into petroleum ether (100 mL), precipitating a yellow viscous crude product. The crude material was washed repeatedly with petroleum ether and further purified by column chromatography using petroleum ether/ethyl acetate (4:1, v/v) as the eluent to afford **AOU3** (3.81 g, yield: 85%). ^{1}H NMR (500 MHz, DMSO-d_6) δ 8.03 (t, J = 5.8 Hz, 1H), 7.46 – 7.31 (m, 5H), 5.38 (s, 2H), 3.12 (td, J = 7.0, 5.7 Hz, 2H), 2.49 (s, 3H), 1.53 – 1.40 (m, 2H), 1.35 – 1.20 (m, 2H), 0.89 (t, J = 7.4 Hz, 3H). ^{13}C NMR (126 MHz, DMSO-d_6) δ 193.48, 160.32, 152.59, 151.98, 135.07, 128.98, 128.69, 67.98, 31.54, 26.12, 19.79, 14.05.

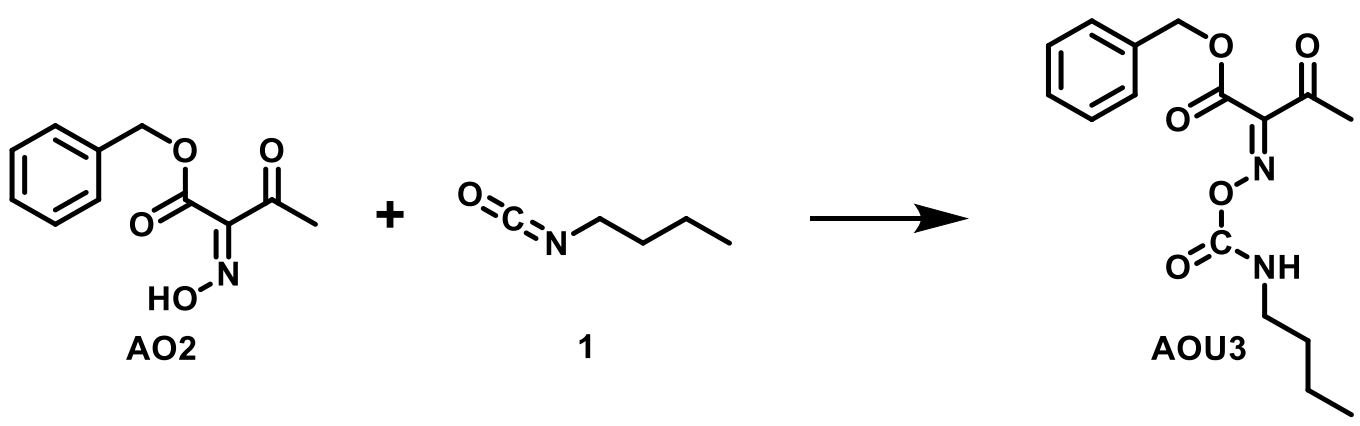

Synthesis of benzyl (Z)-3-oxo-2-(((phenethylcarbamoyl)oxy)imino)butanoate (AOU4)

Phenethyl isocyanate **2** (2.50 g, 0.017 mol) was dissolved in toluene (30 mL) in a flask equipped with a magnetic stir bar. A solution of **AO2** (3.09 g, 0.014 mol) in toluene (30 mL) was then added dropwise. The reaction progress was monitored by TLC. Upon completion, the reaction mixture was concentrated to approximately 10 mL and poured into petroleum ether (100 mL), precipitating a yellow viscous crude

product. The crude material was washed repeatedly with petroleum ether and further purified by column chromatography using petroleum ether/ethyl acetate (5:1, v/v) as the eluent to afford **AOU4** (4.23 g, yield: 82%). ^{1}H NMR (500 MHz, DMSO-d_6) δ 8.12 (t, J = 5.7 Hz, 1H), 7.48 – 7.17 (m, 10H), 5.38 (s, 2H), 3.34 (s, 2H), 2.82 (t, J = 7.4 Hz, 2H), 2.48 (s, 3H). ^{13}C NMR (126 MHz, DMSO-d_6) δ 193.44, 160.27, 152.56, 151.99, 139.30, 135.03, 129.14, 129.01, 128.92, 128.88, 128.69, 126.74, 68.03, 42.69, 35.40, 26.14.

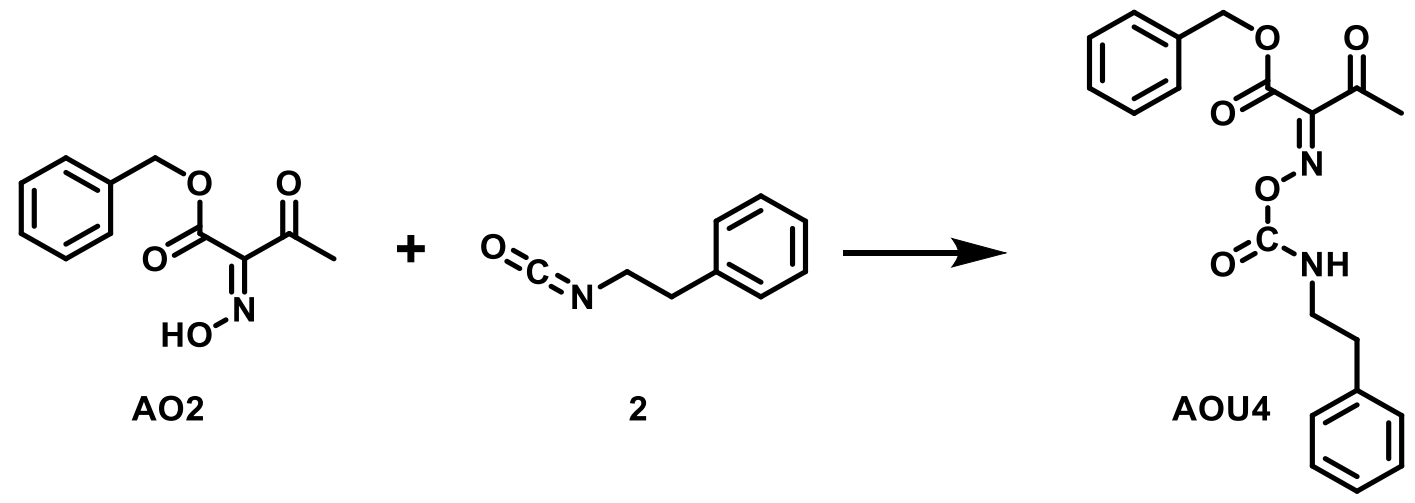

Synthesis of (E)-butan-2-one O-butylcarbamoyl oxime (OU1)

n-Butyl isocyanate **1** (2.28 g, 0.023 mol) was dissolved in anhydrous ether (20 mL) in a flask equipped with a magnetic stir bar. A solution of **BO** (1.66 g, 0.019 mol) in anhydrous ether (15 mL) was then added dropwise. The reaction progress was monitored by TLC. Upon completion, the reaction mixture was concentrated to approximately 10 mL and poured into petroleum ether (100 mL), precipitating a crude product. The crude product was washed repeatedly with petroleum ether and further purified by column chromatography using petroleum ether/ethyl acetate (6:1, v/v) as the eluent to afford product **OU1** (3.11 g, yield: 88%).^{1}H NMR (500 MHz, DMSO-d_6) δ 7.23 (dt, J = 19.5, 5.8 Hz, 1H), 3.06 (qd, J = 6.3, 3.5 Hz, 2H), 2.31 (dq, J = 41.0, 7.6 Hz, 2H), 1.92 (d, J = 10.7 Hz, 3H), 1.43 (pd, J = 8.1, 7.4, 2.2 Hz, 2H), 1.33 – 1.21 (m, 2H), 1.05 (dt, J = 18.6, 7.6 Hz, 3H), 0.88 (td, J = 7.4, 1.2 Hz, 3H). ^{13}C NMR (126 MHz, DMSO-d_6) δ 164.39, 155.36, 40.44, 31.93, 28.93, 19.86, 14.96, 14.04, 10.82.

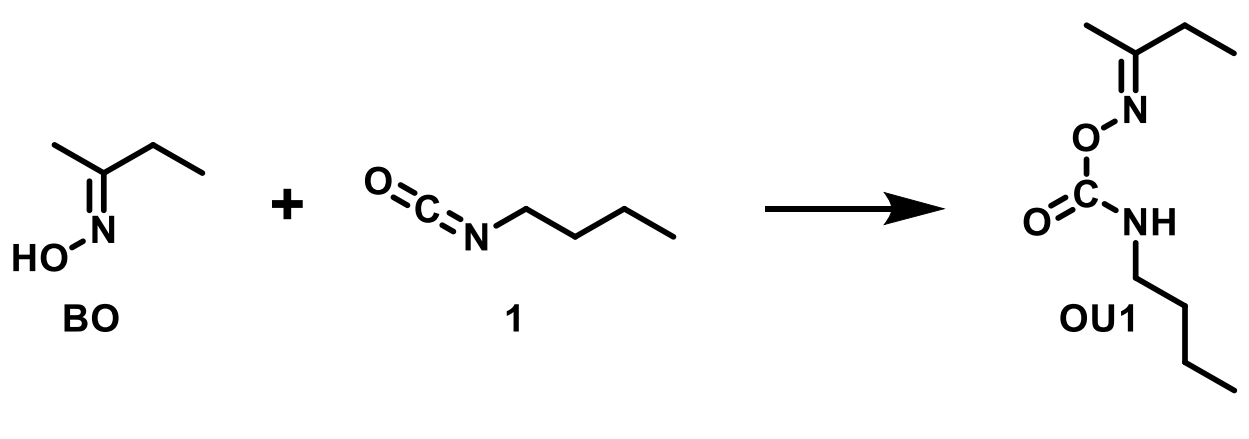

Synthesis of (E)-butan-2-one O-phenethylcarbamoyl oxime (OU2)

Phenethyl isocyanate **2** (2.50 g, 0.017 mol) was dissolved in anhydrous ether (30 mL) in a flask equipped with a magnetic stir bar. A solution of **BO** (1.22 g, 0.014 mol) in anhydrous ether (10 mL) was then added dropwise. The reaction progress was monitored by TLC. Upon completion, the reaction mixture was concentrated to approximately 10 mL and poured into petroleum ether (100 mL), precipitating a crude

product. The crude product was washed repeatedly with petroleum ether and further purified by column chromatography using petroleum ether/ethyl acetate (3:1, v/v) as the eluent to afford product **OU2** (2.82 g, yield: 86%). ^{1}H NMR (500 MHz, DMSO-d_6) δ 7.32 – 7.13 (m, 6H), 3.32 (dt, J = 8.1, 6.2 Hz, 2H), 2.78 (t, J = 7.6 Hz, 2H), 2.31 (dq, J = 41.2, 7.6 Hz, 2H), 1.92 (d, J = 10.3 Hz, 3H), 1.04 (dt, J = 14.7, 7.6 Hz, 3H). ^{13}C NMR (126 MHz, DMSO-d_6) δ 164.66, 155.36, 139.63, 129.07, 128.81, 126.58, 42.41, 35.84, 28.98, 15.11, 10.76.

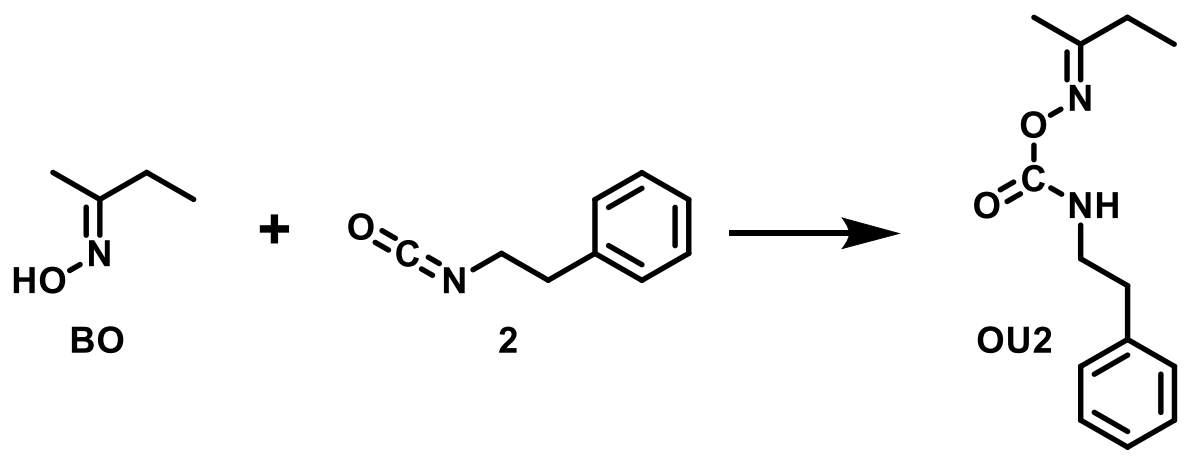

Variable-temperature NMR experiments

AOU1 (0.026 g, 0.01 mmol) and phenethyl isocyanate **2** (0.015 g, 0.01 mmol) were added into 0.6 mL anhydrous DMSO-d_6 in a NMR tube. ^{1}H NMR spectrum was collected immediately after simple mixing, and the mixture was then maintained at a certain temperature (90 °C, 100 °C, 110 °C, 120 °C), with ^{1}H NMR spectra collected at different times. ^{1}H NMR spectra of the reaction between **OU1** and phenethyl isocyanate **2** were collected using a similar method.

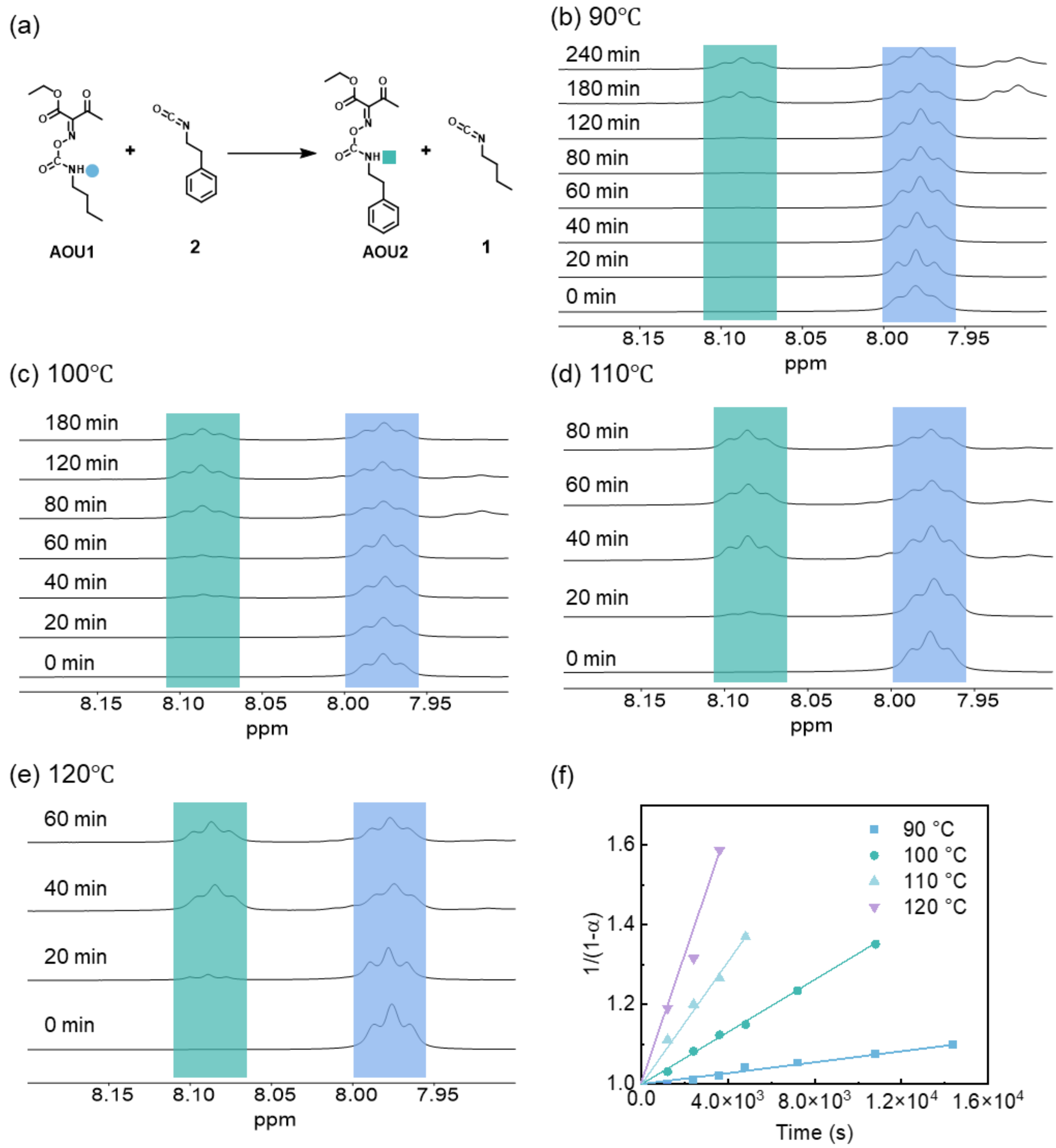

Figure S10. (a) The exchange reaction of **AOU1** with **2**, and the corresponding ^{1}H NMR spectra recorded during the exchange reaction at (b) 90 °C, (c) 100 °C, (d) 110 °C, (e) 120 °C. (f) Fitted curves of the conversion rate (α) of **AOU1** in the exchange reaction *versus* time at different temperatures.

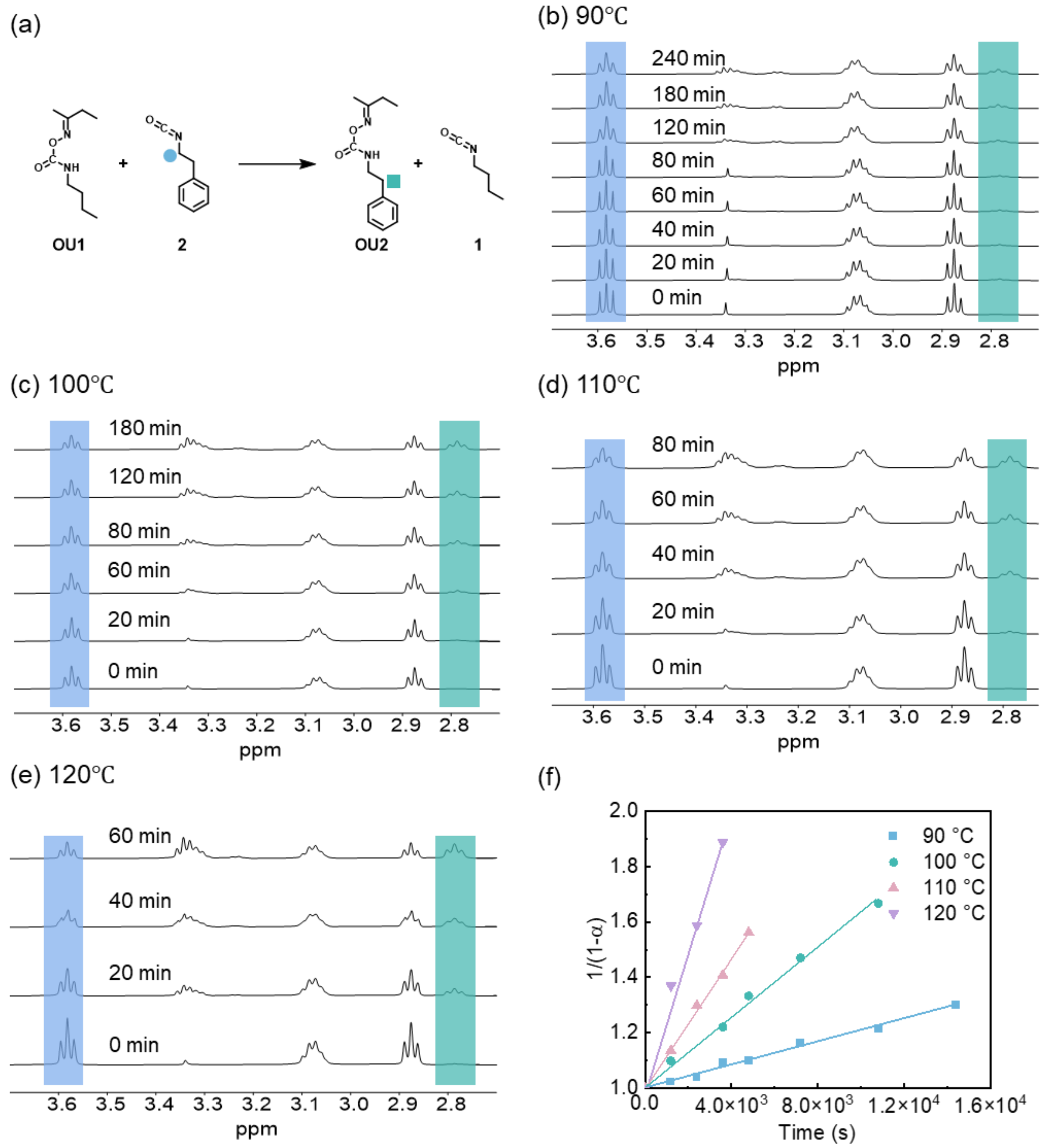

Figure S11. (a) The exchange reaction of **OU1** with **2**, and the corresponding ^{1}H NMR spectra recorded during the exchange reaction at (b) 90 °C, (c) 100 °C, (d) 110 °C, (e) 120 °C. (f) Fitted curves of the conversion rate (α) of **OU1** in the exchange reaction *versus* time at different temperatures.

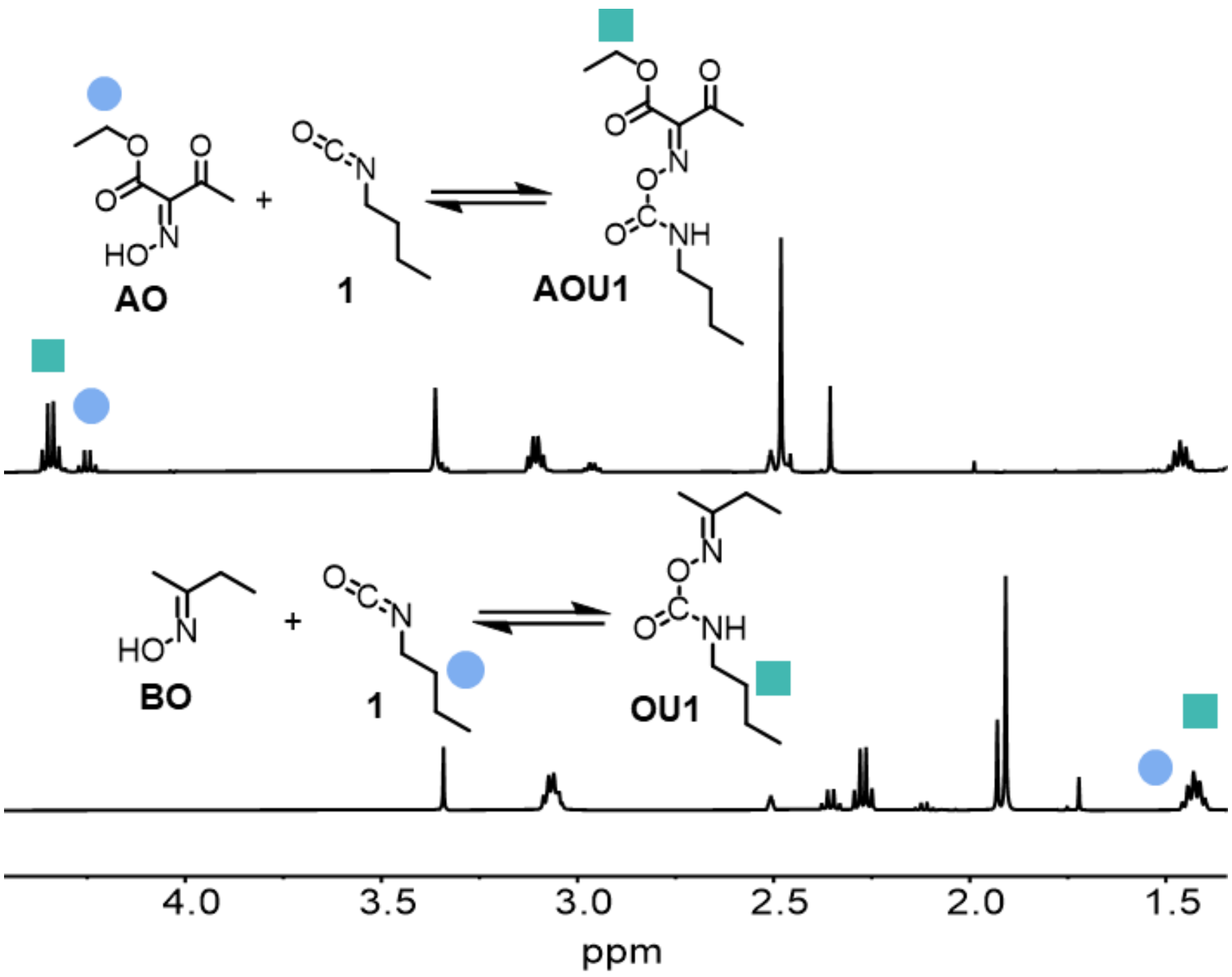

Figure S12. ^{1}H NMR spectra of the mixture of **1** and **AO** or **BO**, measured after the reaction at 25 °C for 48 h.

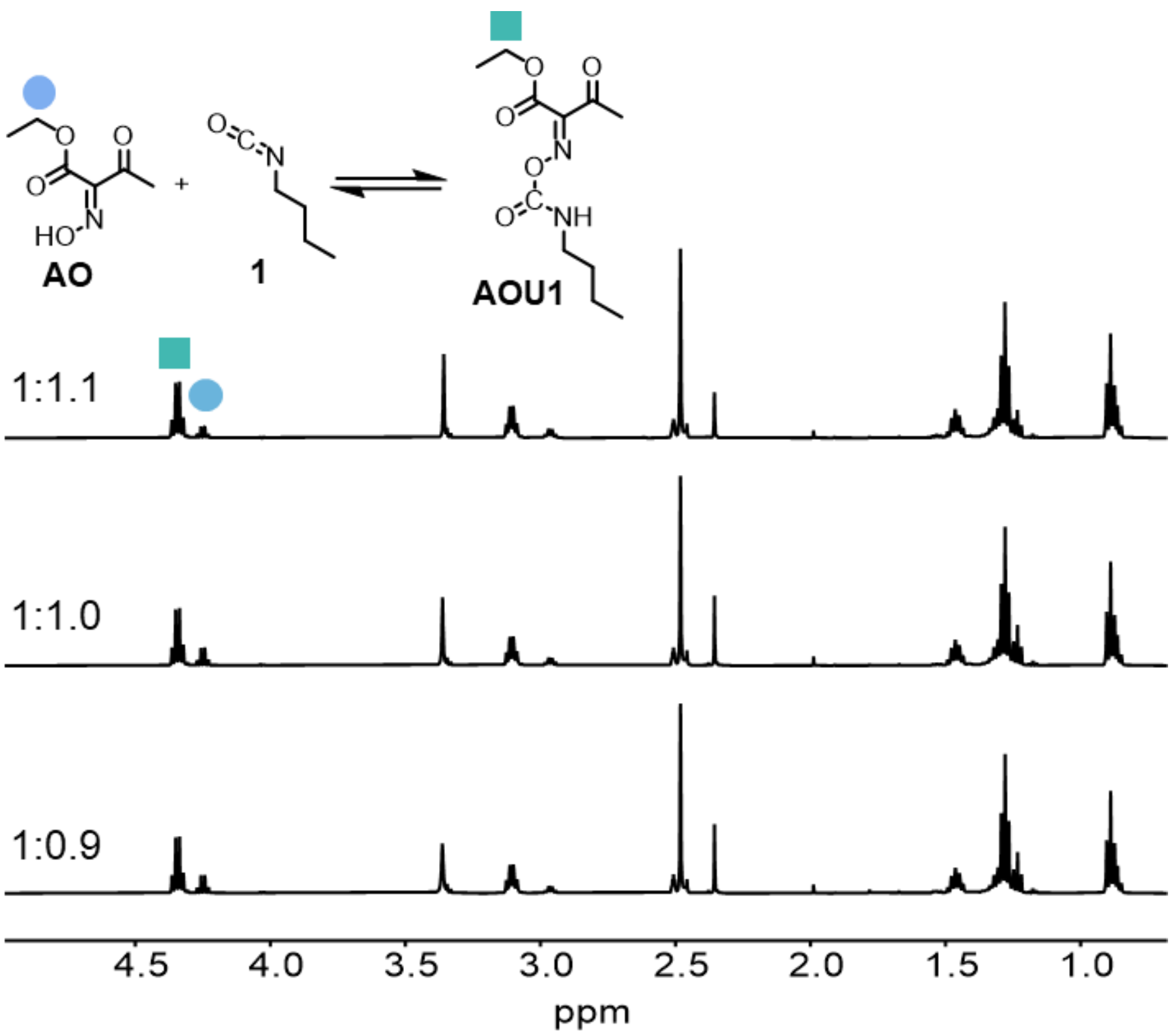

Figure S13. ^{1}H NMR spectra of **AO** and different equivalents of **2** after reacting at room temperature for 48 h. The reaction was carried out in NMR tubes using DMSO-d_6 as the solvent, and monitoring the variation in ^{1}H NMR spectra determines that the reaction reaches equilibrium at 48 h.

Table S2. The concentrations of $\mathbf{[AO]_{eq}}$, $\mathbf{[1]_{eq}}$ and $\mathbf{[AOU1]_{eq}}$ at equilibrium, as well as the equilibrium constant K_{eq}, were calculated from the integration of ^{1}H NMR spectra shown in Figure S13. $\mathbf{[AO]_0}$ and $\mathbf{[1]_0}$ are the initial concentrations.

	$\mathbf{[AO]_0}$ (M)	$\mathbf{[1]_0}$ (M)	$\mathbf{[AO]_{eq}}$ (M)	$\mathbf{[1]_{eq}}$ (M)	$\mathbf{[AOU1]_{eq}}$ (M)	K_{eq} (M^{-1})
1:1.1	0.371	0.404	0.063	0.096	0.308	50.93
1:1	0.366	0.368	0.065	0.067	0.301	69.12
1:0.9	0.367	0.337	0.069	0.039	0.298	110.74

Table S3. The concentrations of $\mathbf{[BO]_{eq}}$, $\mathbf{[1]_{eq}}$ and $[\mathbf{OU1}]_{eq}$ at equilibrium, as well as the equilibrium constant K_{eq}, were calculated from the integration of ^{1}H NMR spectra shown in Figure S12. $\mathbf{[BO]_0}$ and $\mathbf{[1]_0}$ are the initial concentrations.

$\mathbf{[BO]_0}$ (M)	$\mathbf{[1]_0}$ (M)	$\mathbf{[BO]_{eq}}$ (M)	$\mathbf{[1]_{eq}}$ (M)	$[\mathbf{OU1}]_{eq}$ (M)	K_{eq} (M^{-1})
0.364	0.365	0.008	0.009	0.356	4944.44

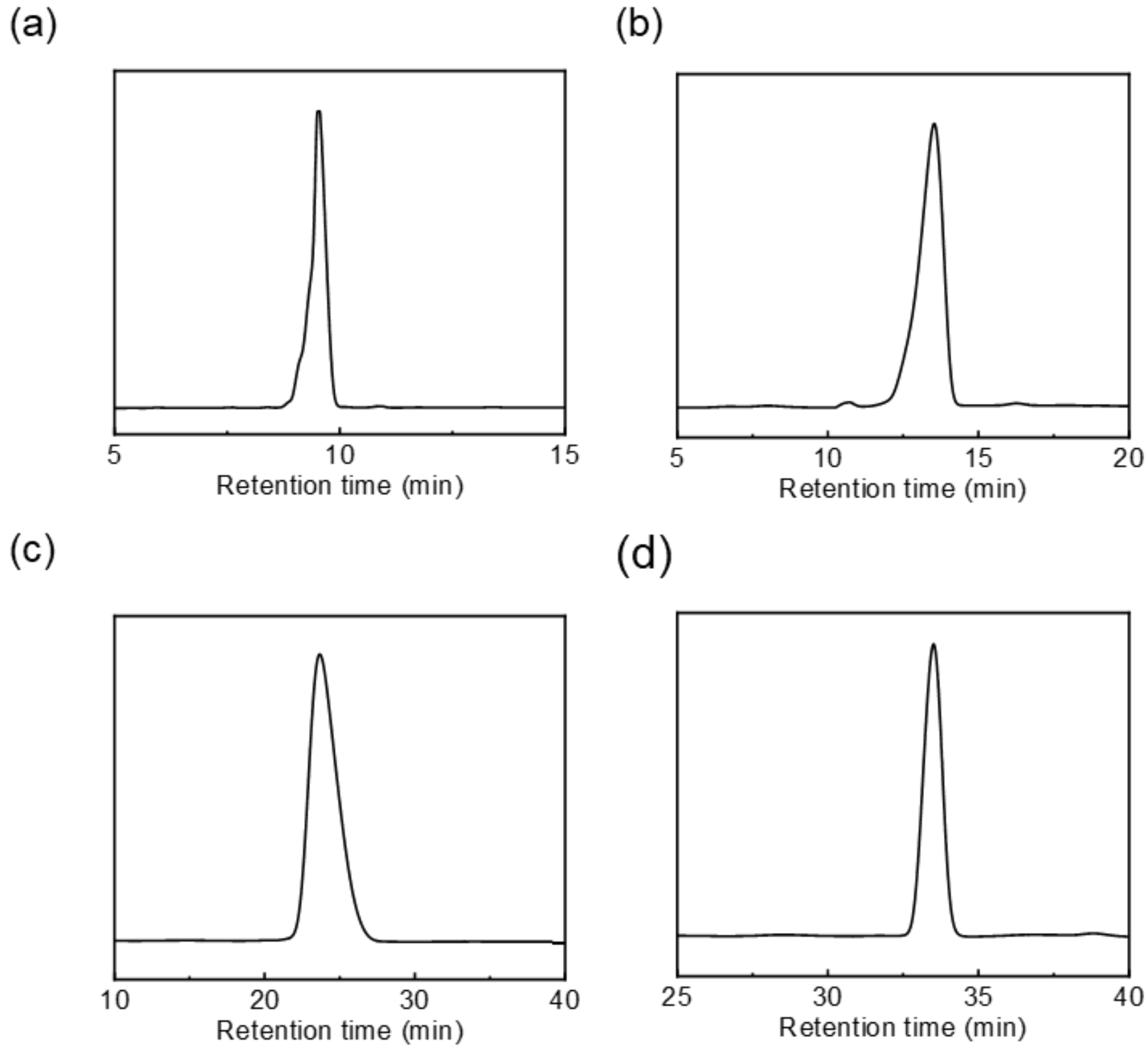

Figure S14. HPLC chromatograms of **AOU1** (a), **AOU2** (b), **AOU3** (c), and **AOU4** (d).

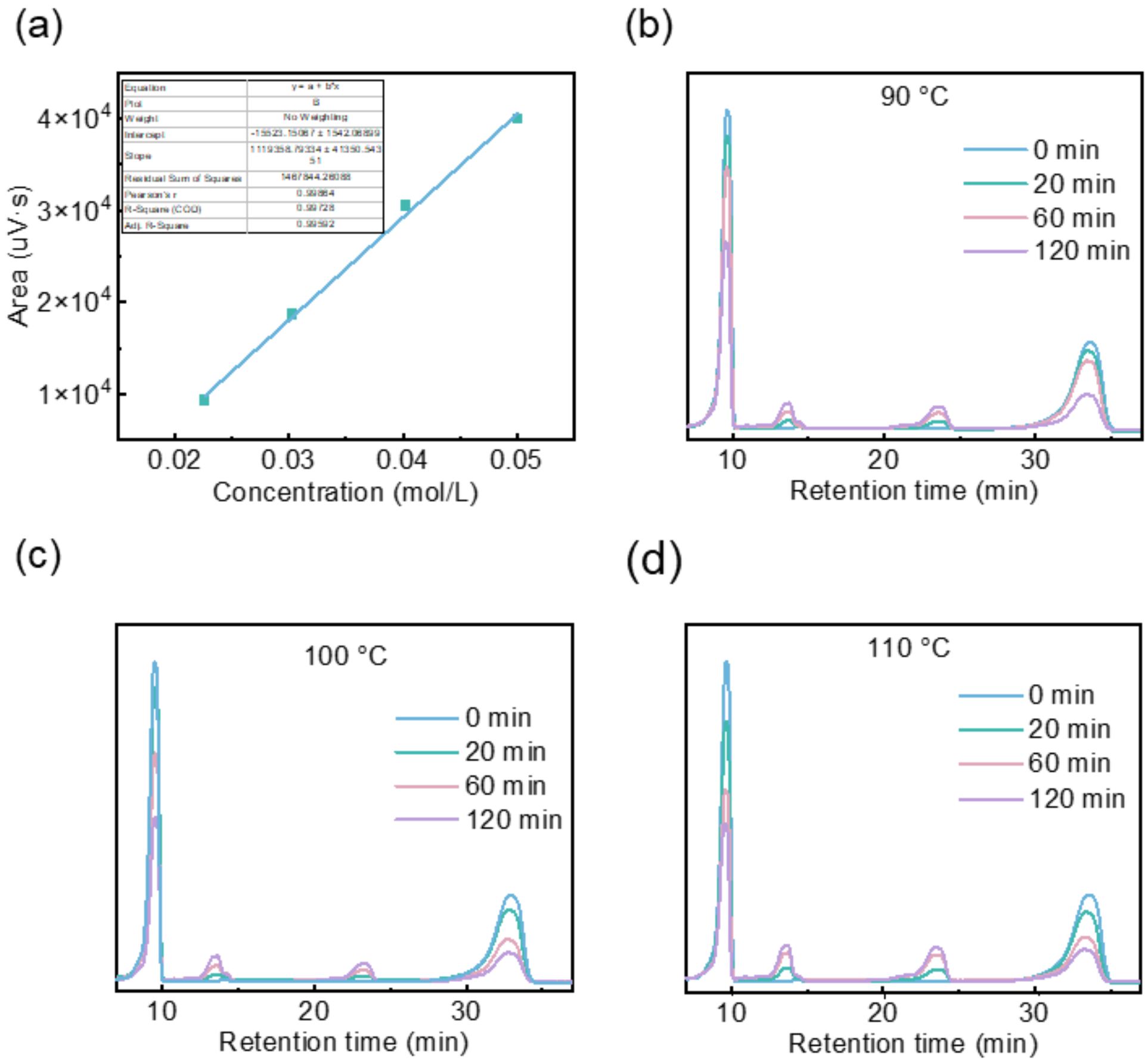

Figure S15. (a) HPLC standard curve of **AOU1**. HPLC chromatograms of the mixture of **AOU1** and **AOU4** after the reaction at (b) 90 °C, (c) 100 °C, (d) 110 °C for different times.

Computational details

All calculations were performed with the Gaussian 16 program package. Density functional theory (DFT) adopting the M06-2X functional and def2-TZVP basis set were applied to optimize all stationary points, with solvation effects in tetrahydrofuran (THF) described by the SMD (Solvation Model based on Density) model. The vibrational frequencies were computed at the same level of theory to check whether each optimized structure is an energy minimum or a transition state and to evaluate its zero-point energy (*ZPE*) and the thermal corrections to Gibbs free energy at 298 K (all ΔG_{sol} were based on these calculations). Intrinsic reaction coordinate (IRC) calculations were used to confirm that the transition states connect the corresponding reactants and products in the solvent calculations. In this article, all discussed energies are Gibbs free energies in THF (ΔG_{sol}) at 298 K unless specified.

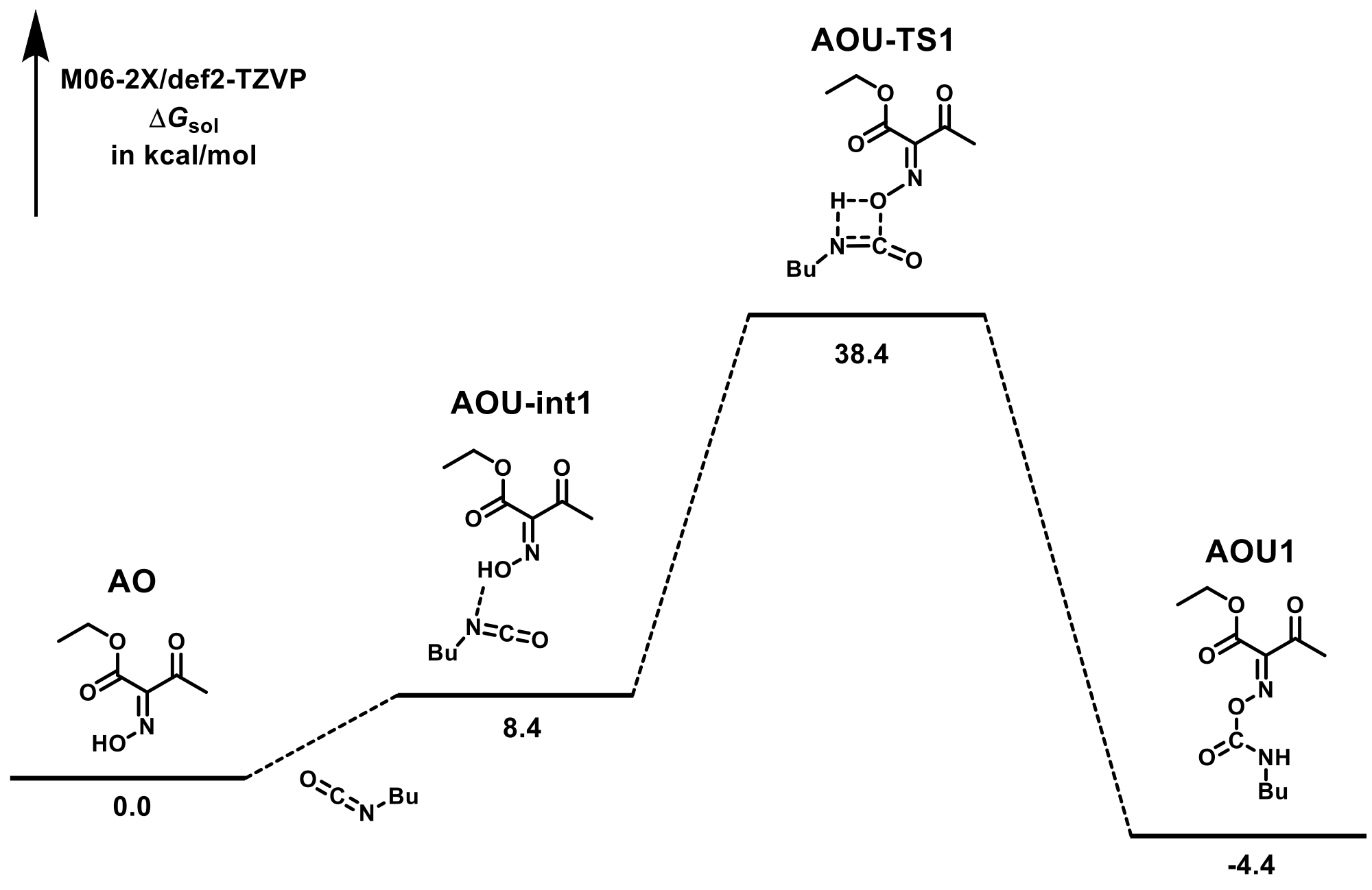

Figure S16. DFT-calculated relative energies for the addition of AO to *n*-butyl isocyanate through concerted four-membered ring mechanism. Calculations showed that such process is not favored with an activation free energy of as high as 38.4 kcal/mol.

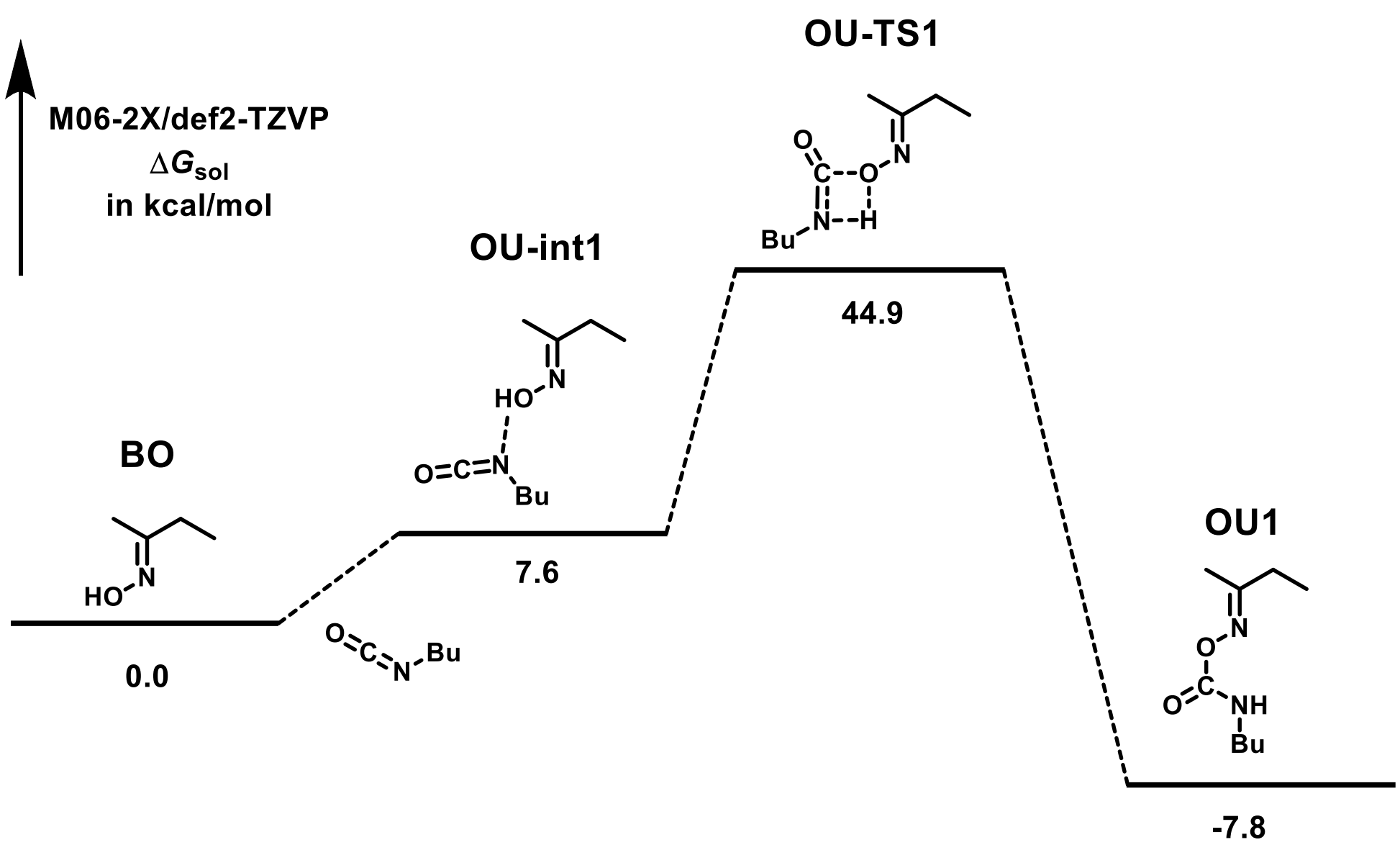

Figure S17. DFT-calculated relative energies for the addition of **BO** to *n*-butyl isocyanate through concerted four-membered ring mechanism. This pathway is disfavored due to the high activation free energy of 44.9 kcal/mol.

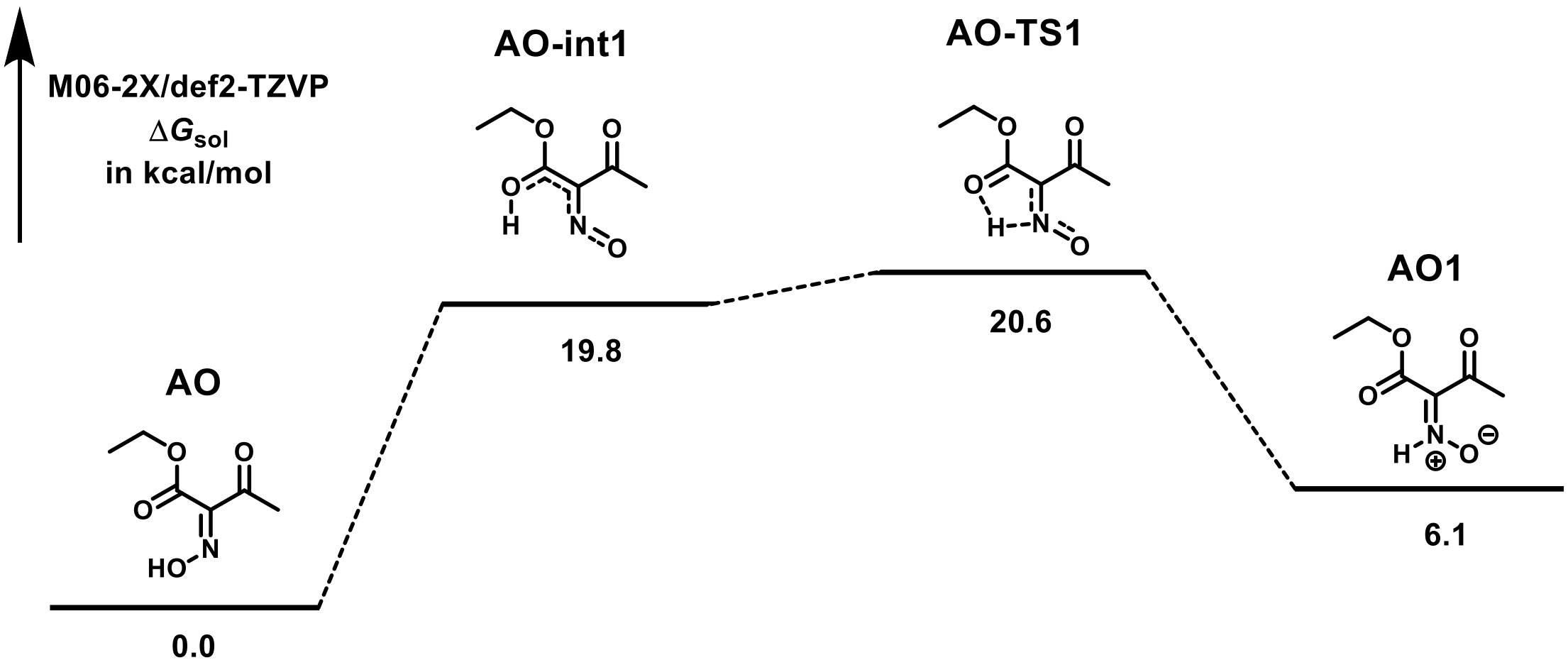

Figure S18. DFT-calculated relative energies for intramolecular tautomerism mechanism of **AO** to **AO1**.

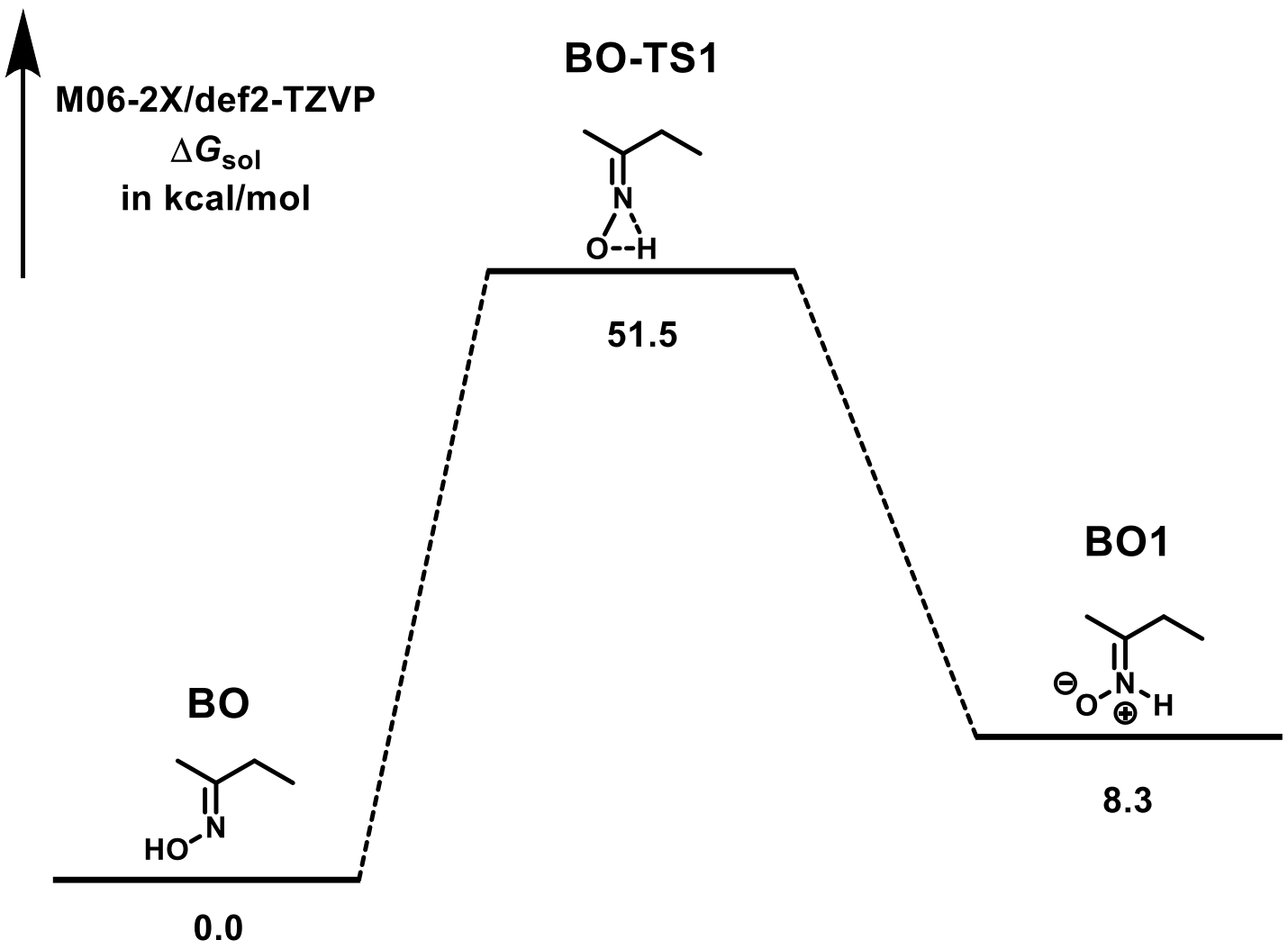

Figure S19. DFT-calculated relative energies for intramolecular tautomerism mechanism of **BO** to **BO1**. The intramolecular shift is difficult with an activation free energy as high as 51.5 kcal/mol, suggesting that this is not the pathway of isomerization.

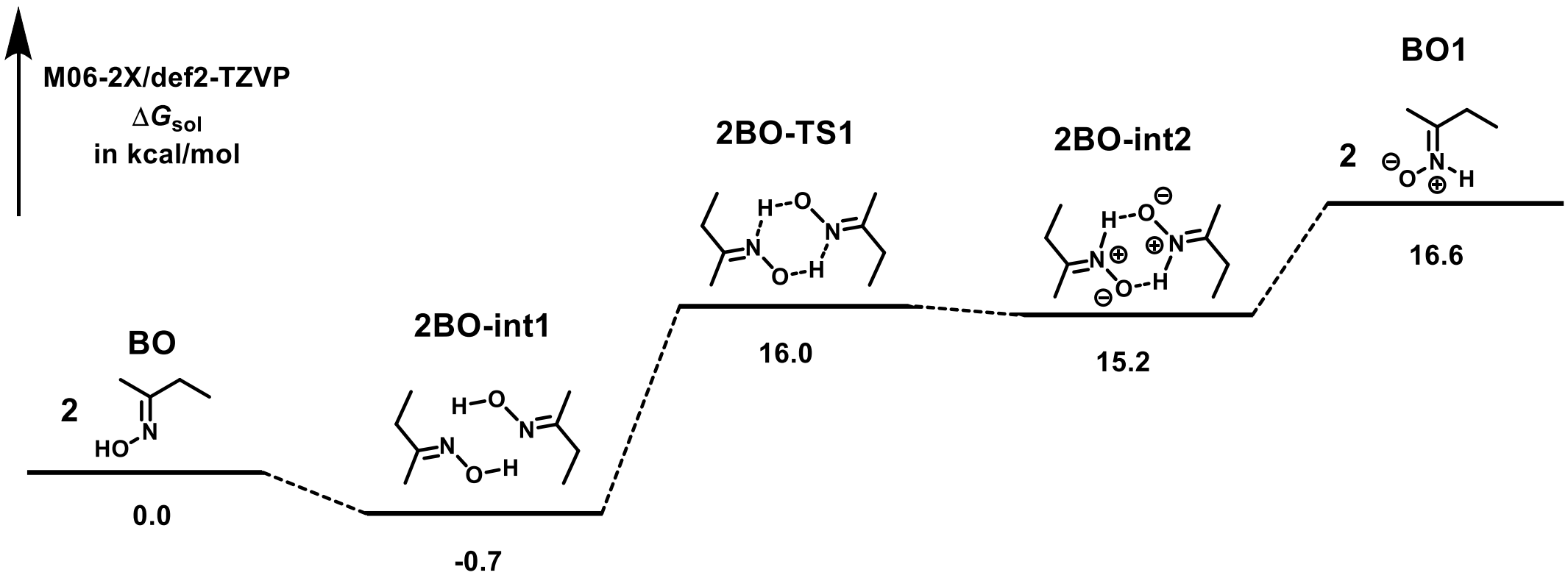

Figure S20. DFT-calculated relative energies for bimolecular tautomerism mechanism of **BO** to **BO1**.

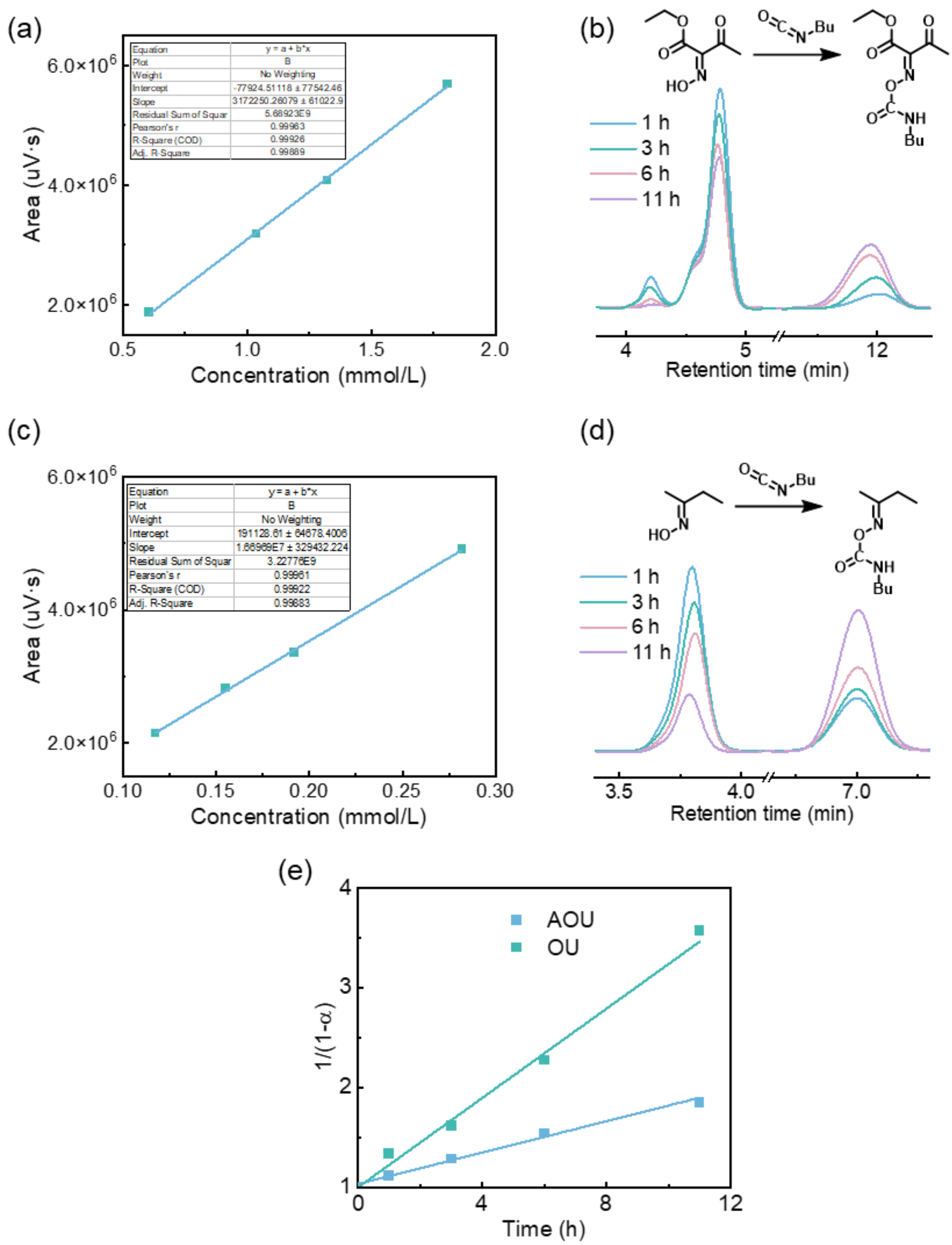

Figure S21. HPLC standard curve of (a) **AO** and (c) **BO**. HPLC chromatograms of the mixture of (b) **AO** or (d) **BO** and *n*-butyl isocyanate after the reaction at 25 ℃ for different times. (e) Fitted curves of the conversion rate (α) of **AO** or **BO** in the **AOU** or **OU** synthesis reaction *versus* time.

References

[1] Y. Zhang, H. Ying, K. R. Hart, Y. Wu, A. J. Hsu, A. M. Coppola, T. A. Kim, K. Yang, N. R. Sottos, S. R. White, J. Cheng, "Malleable and Recyclable Poly(urea-urethane) Thermosets bearing Hindered Urea Bonds" *Adv. Mater.* **2016**, *28*, 7646–7651.

[2] S. Wang, Y. Yang, H. Ying, X. Jing, B. Wang, Y. Zhang, J. Cheng, "Recyclable, Self-Healable, and Highly Malleable Poly(urethane-urea)s with Improved Thermal and Mechanical Performances" *ACS Appl. Mater. Interfaces* **2020**, *12*, 35403–35414.

[3] T. Patel, J. Park, M. P. Kim, Z. Ye, H. Ko, H. W. Jung, J. K. Oh, "Dynamic poly(hindered urea) hybrid network materials crosslinked with reactive methacrylate polymer" *Polym. Chem.* **2023**, *14*, 5115–5124.

[4] B. Zhao, F. Song, L. Tan, R. Yang, Z. Pan, M. Zhang, Y. Zhou, "Production, thermal recycling, and application of cardanol-based polyurethane foam with phenol-carbamate bonds" *Chem. Eng. J.* **2024**, *494*, 152941.

[5] J. Shi, T. Zheng, Y. Zhang, B. Guo, J. Xu, "Reprocessable Cross-Linked Polyurethane with Dynamic and Tunable Phenol–Carbamate Network" *ACS Sustain. Chem. Eng.* **2020**, *8*, 1207–1218.

[6] L. Chevret, T. Flores, M. Coronas, J. Slootman, C. Ravet, M. Petit, V. Ladmiral, C. Bakkali-Hassani, S. Caillol, "Easily Recyclable Phenol-Carbamate Covalent Adaptable Networks from Biobased Isocyanate and Cashew Nutshell Liquid Novolac" *Macromolecules* **2025**, *58*, 7467–7480.

[7] S. Wang, D. Fu, X. Wang, W. Pu, A. Martone, X. Lu, M. Lavorgna, Z. Wang, E. Amendola, H. Xia, "High performance dynamic covalent crosslinked polyacylsemicarbazide composites with self-healing and recycling capabilities" *J. Mater. Chem. A* **2021**, *9*, 4055–4065.

[8] D. Fu, W. Pu, J. Escorihuela, X. Wang, Z. Wang, S. Chen, S. Sun, S. Wang, H. Zuilhof, H. Xia, "Acylsemicarbazide Moieties with Dynamic Reversibility and Multiple Hydrogen Bonding for Transparent, High Modulus, and Malleable Polymers" *Macromolecules* **2020**, *53*, 7914–7924.

[9] Z. Dong, Y. Wang, J. Chen, Z. Ma, J. Ding, T. Liu, N. Jiang, Y. Li, "Acylsemicarbazide-Based Supramolecular Liquid Crystal Elastomers: from Molecular Design to Melt-Processing Actuators" *Adv. Funct. Mater.* **2025**, e24643.

[10] W.-X. Liu, Z. Yang, Z. Qiao, L. Zhang, N. Zhao, S. Luo, J. Xu, "Dynamic multiphase semi-crystalline polymers based on thermally reversible pyrazole-urea bonds" *Nat. Commun.* **2019**, *10*, 4753.

[11] L. Huang, J. Xia, Z. Jin, X. Hu, X. Chen, N. Zhao, C. Gao, W. Liu, "Entropy-driven toughening and closed-loop recycling of polymers via divergent metal-pyrazole interactions" *Nat. Commun.* **2025**, *16*, 10673.

[12] W. X. Liu, C. Zhang, H. Zhang, N. Zhao, Z. X. Yu, J. Xu, "Oxime-Based and Catalyst-Free Dynamic Covalent Polyurethanes" *J. Am. Chem. Soc.* **2017**, *139*, 8678–8684.

[13] L. Zhang, Z. Liu, L. Sun, L. Xiao, Q. Guan, Z. You, "Simple Solvent-Free Strategy for Synthesizing Covalent Adaptable Networks from Commodity Vinyl Monomers" *Macromolecules* **2021**, *54*, 4081–4088.

[14] D. Fu, W. Pu, Z. Wang, X. Lu, S. Sun, C. Yu, H. Xia, "A facile dynamic crosslinked healable poly(oxime-urethane) elastomer with high elastic recovery and recyclability" *J. Mater. Chem. A* **2018**, *6*, 18154–18164.

[15] Y. Ma, X. Jiang, J. Yin, C. Weder, J. A. Berrocal, Z. Shi, "Chemical Upcycling of Conventional Polyureas into Dynamic Covalent Poly(aminoketoenamide)s" *Angew. Chem. Int. Ed.* **2023**, *62*, e202212870.

[16] Z. Liu, C. Yu, C. Zhang, Z. Shi, J. Yin, "Revisiting Acetoacetyl Chemistry to Build Malleable Cross-Linked Polymer Networks via Transamidation" *ACS Macro Lett.* **2019**, *8*, 233–238.

[17] Y. Ma, J. A. Berrocal, X. Jiang, Z. Shi, "Fast (Re)processing of Urea-Containing Polymers Enabled by Dynamic Aminoketoenamide Bonds" *ACS Sustain. Chem. Eng.* **2023**, *11*, 7917–7923.

[18] Z. Liu, L. Liu, Y. Li, K. Yang, S. Li, W. Li, Y. Zhu, T. Xie, "Atom economic closed-loop recycling of thermoset polyurethane foams" *Nat. Commun.* **2025**, *16*, 5117.

[19] Z. Liu, Z. Fang, N. Zheng, K. Yang, Z. Sun, S. Li, W. Li, J. Wu, T. Xie, "Chemical upcycling of commodity thermoset polyurethane foams towards high-performance 3D photo-printing resins" *Nat. Chem.* **2023**, *15*, 1773–1779.

[20] X. Zhang, X. Pu, L. Xia, W. Peng, Y. Ji, J. Xu, J. Wu, L. Jiang, Q. Zhao, Z. Fang, T. Xie, "Solvent- and catalyst-free bio-conversion of waste polyurethane foams into high-performance 3D printing resin" *Natl. Sci. Rev.* **2025**, *12*, nwaf501.

[21] R. M. O'Dea, M. Nandi, G. Kroll, J. R. Arnold, L. T. J. Korley, T. H. Epps, "Toward Circular Recycling of Polyurethanes: Depolymerization and Recovery of Isocyanates" *JACS Au* **2024**, *4*, 1471–1479.

Appendix of NMR spectra

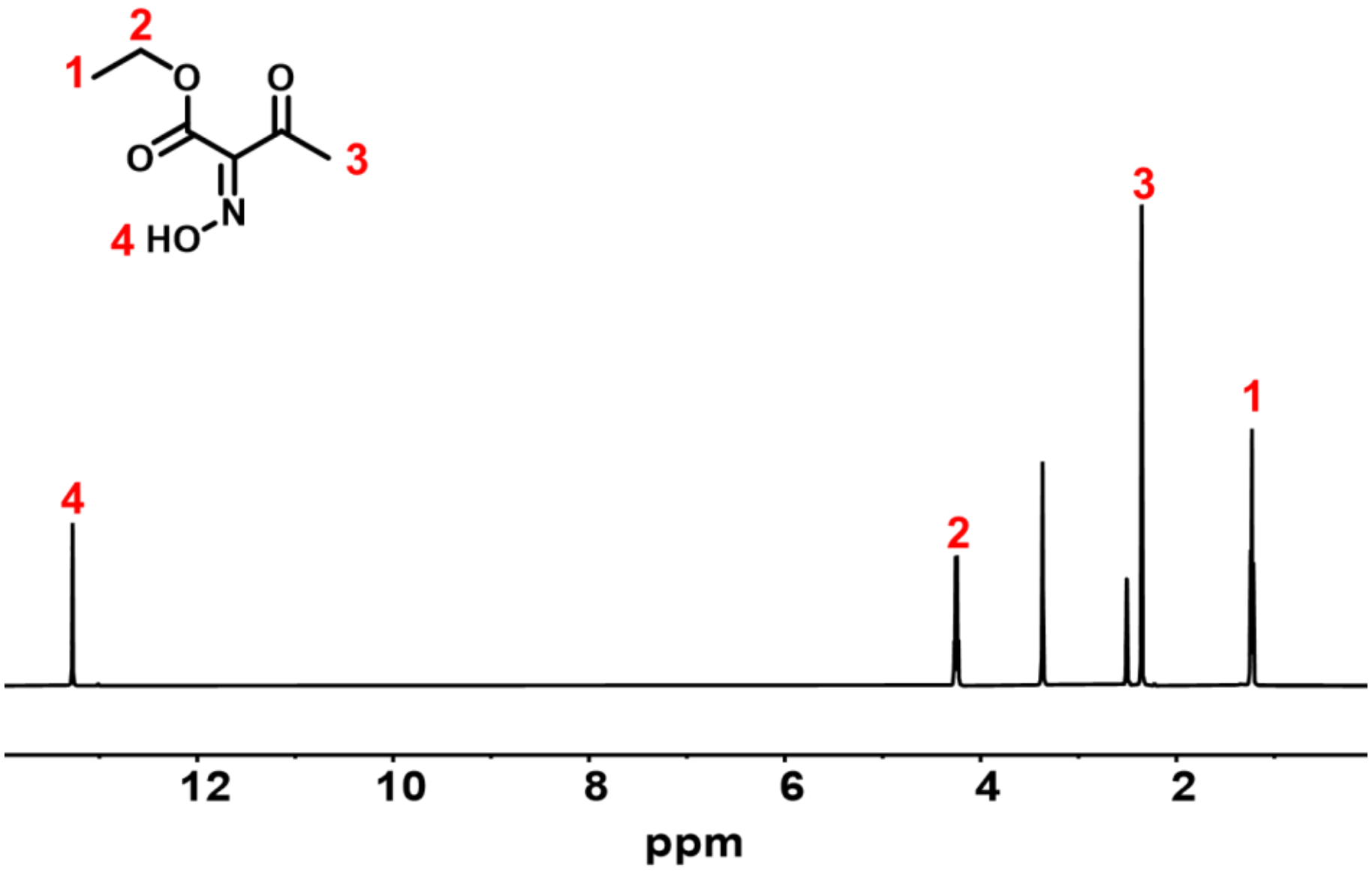

Figure S22. ^{1}H NMR (500 MHz, DMSO-d_6) spectrum of ethyl (Z)-2-(hydroxyimino)-3-oxobutanoate (AO).

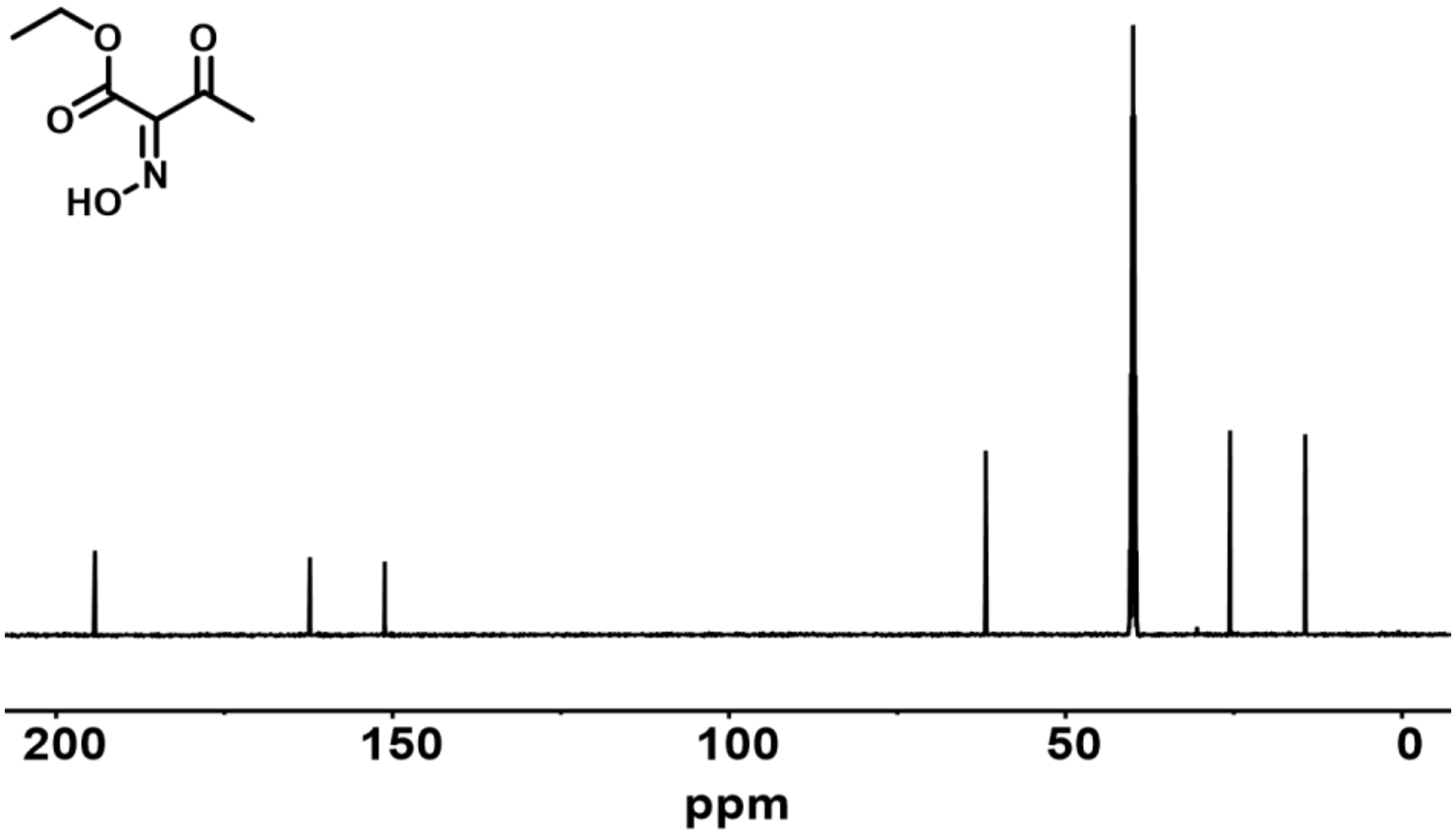

Figure S23. ^{13}C NMR (500 MHz, DMSO-d_6) spectrum of AO.

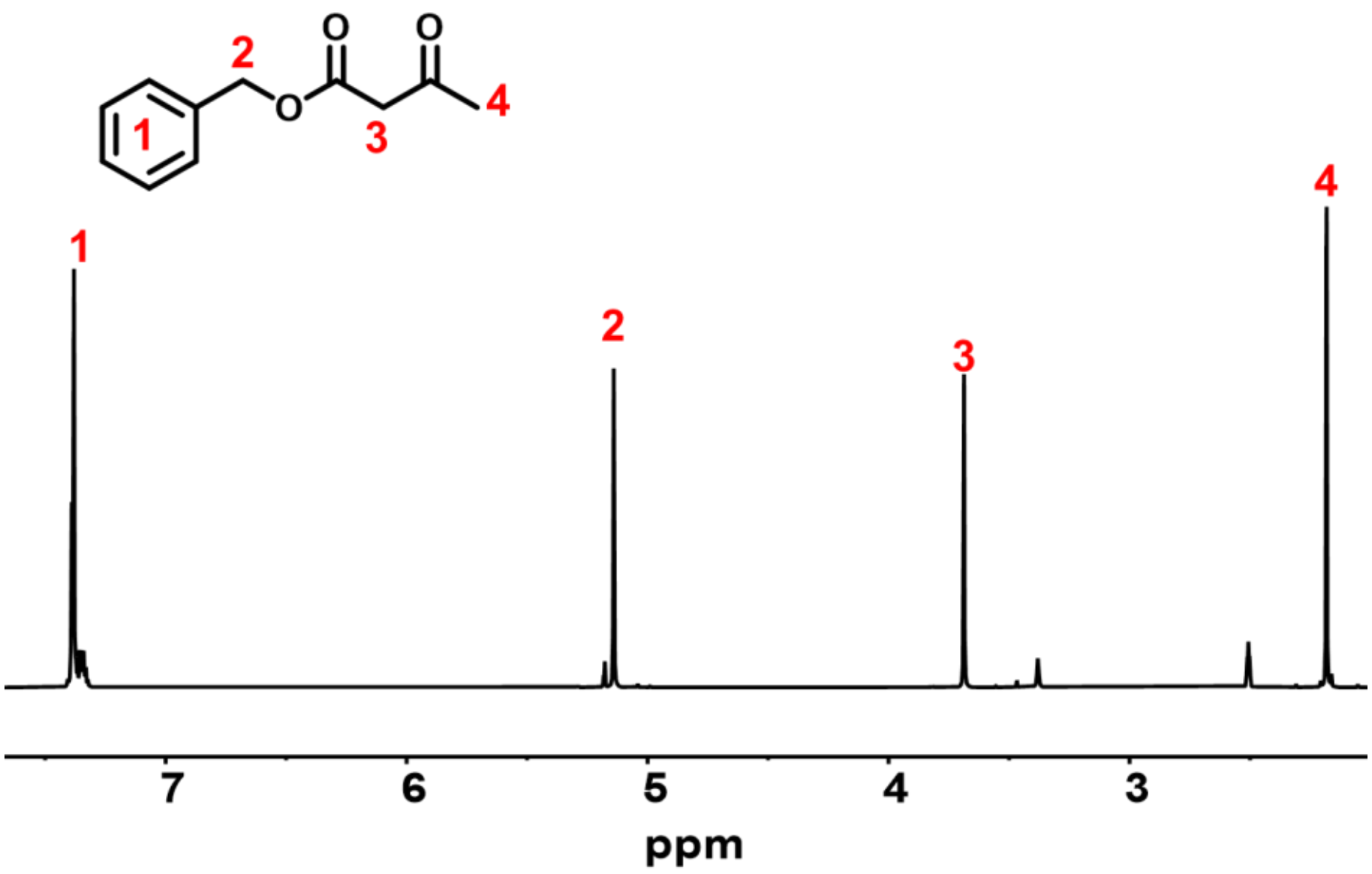

Figure S24. ^{1}H NMR (500 MHz, DMSO-d_6) spectrum of benzyl 3-oxobutanoate.

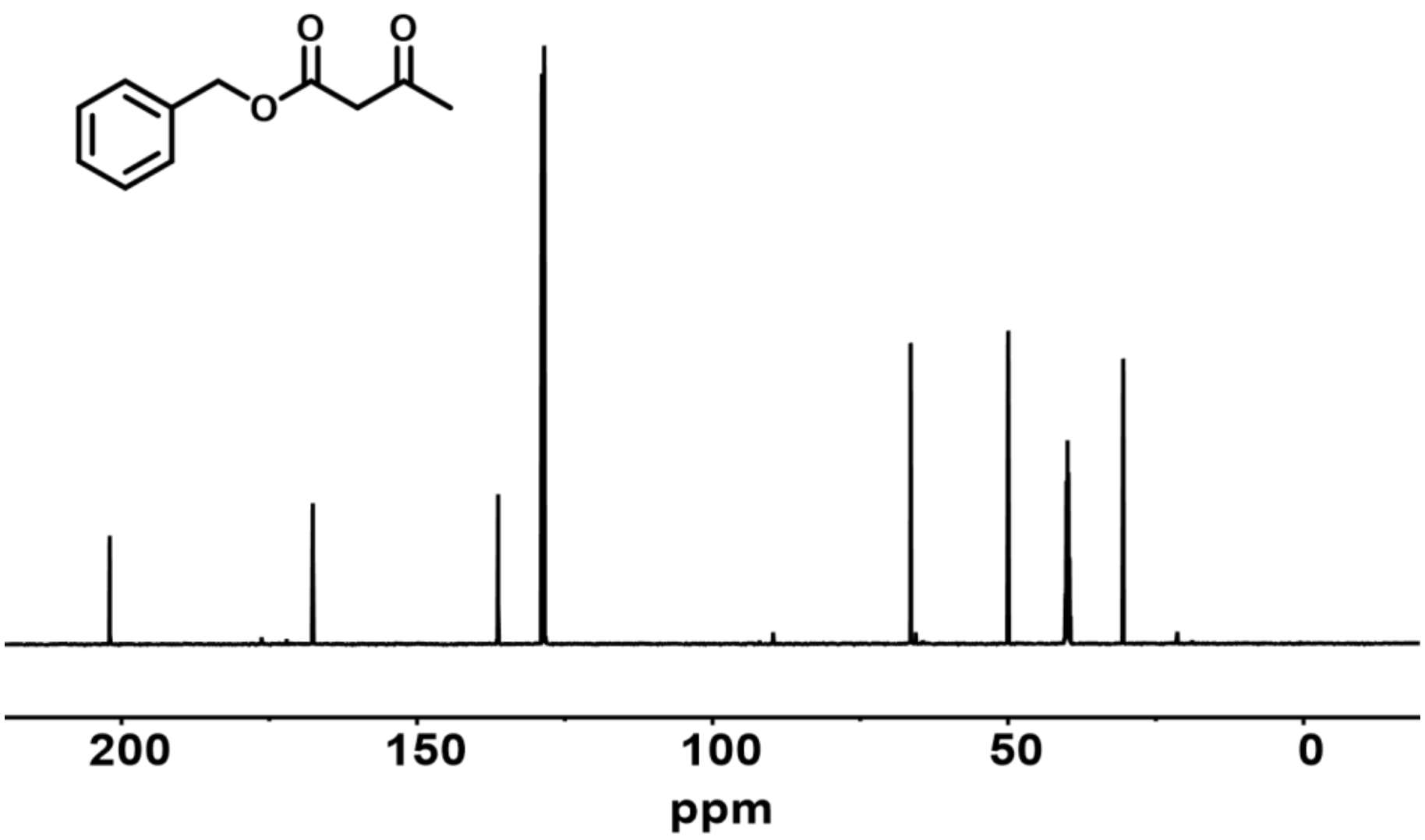

Figure S25. ^{13}C NMR (500 MHz, DMSO-d_6) spectrum of benzyl 3-oxobutanoate.

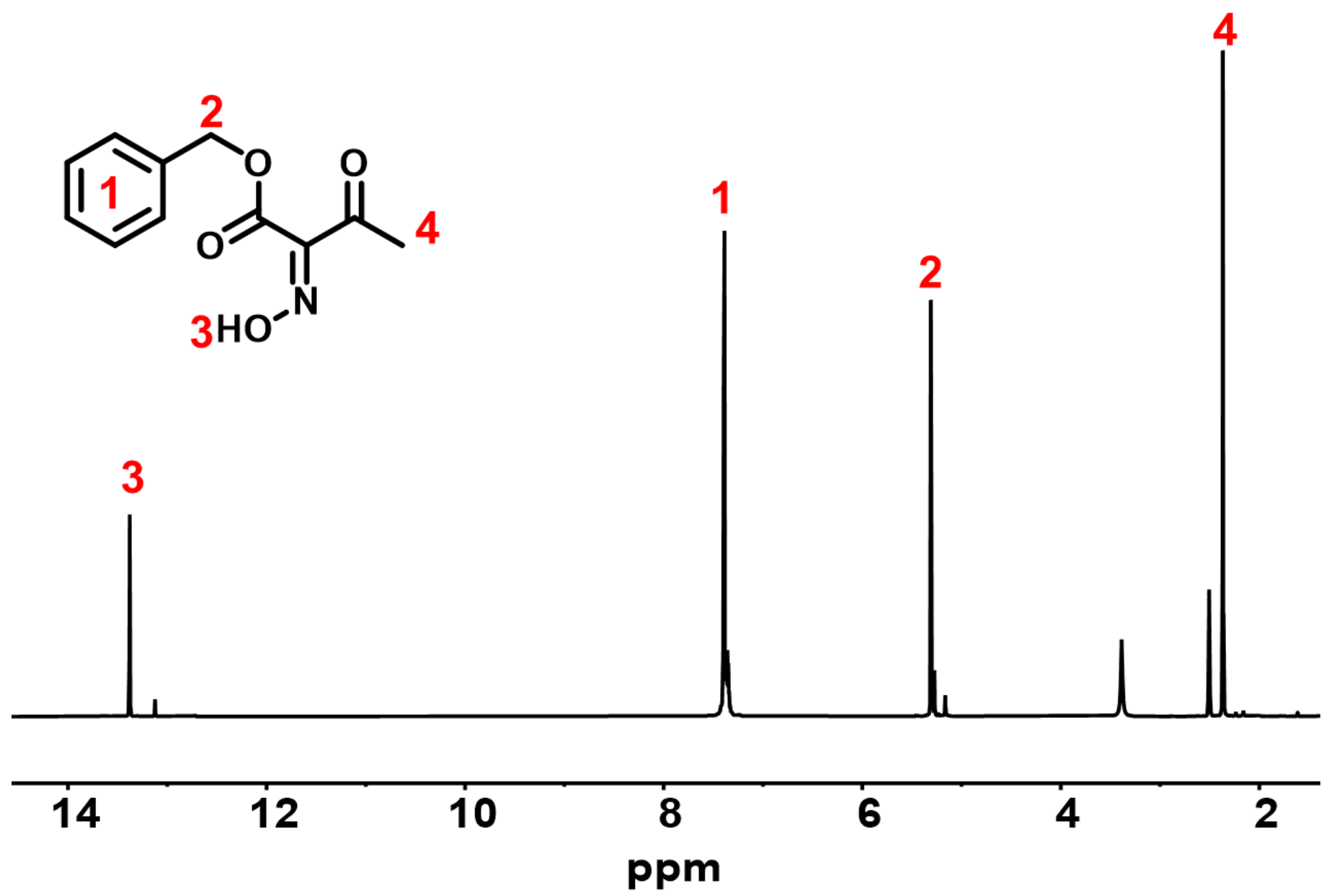

Figure S26. ^{1}H NMR (500 MHz, DMSO-d_6) spectrum of benzyl (Z)-2-(hydroxyimino)-3-oxobutanoate (AO2).

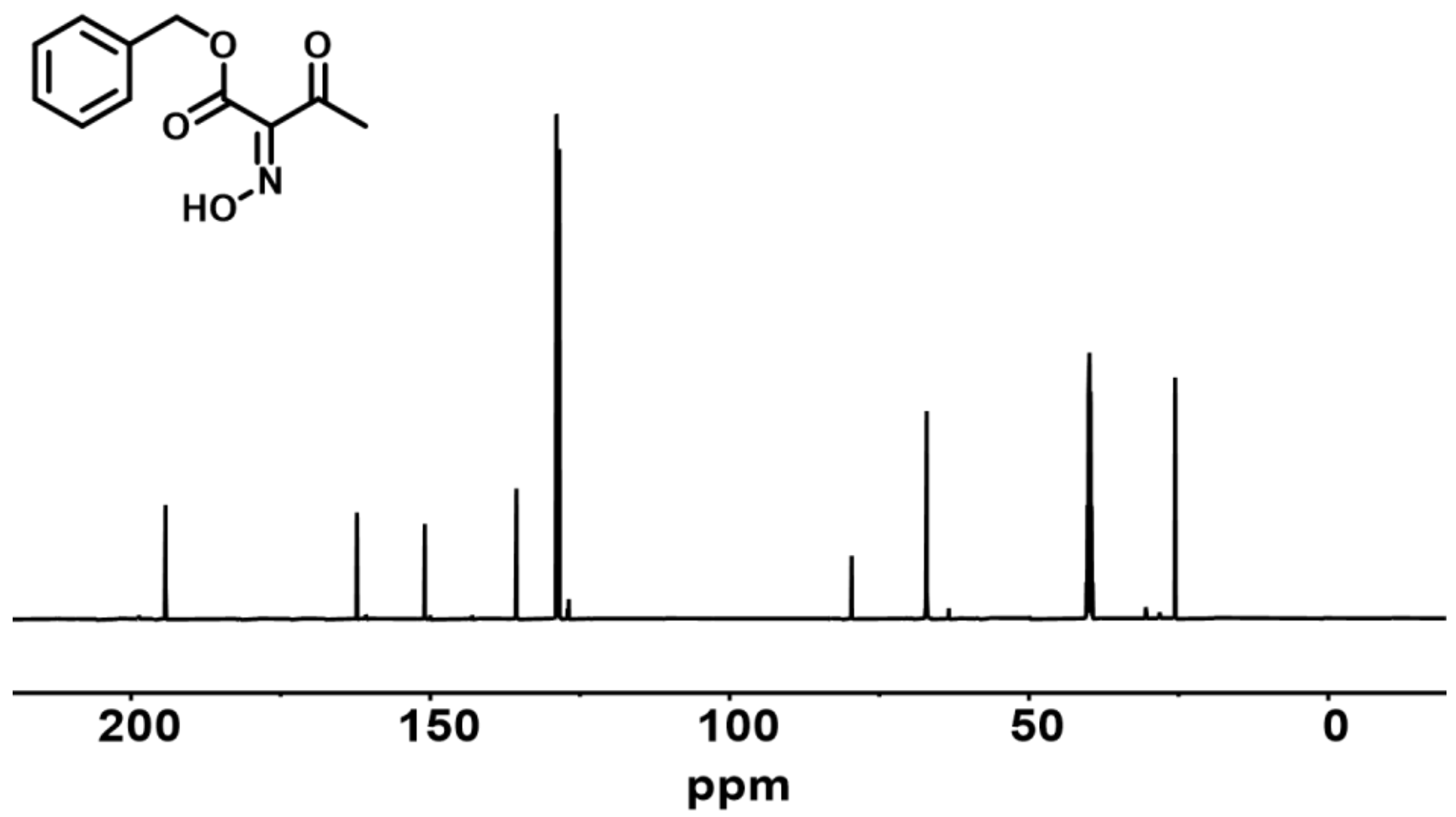

Figure S27. ^{13}C NMR (500 MHz, DMSO-d_6) spectrum of AO2.

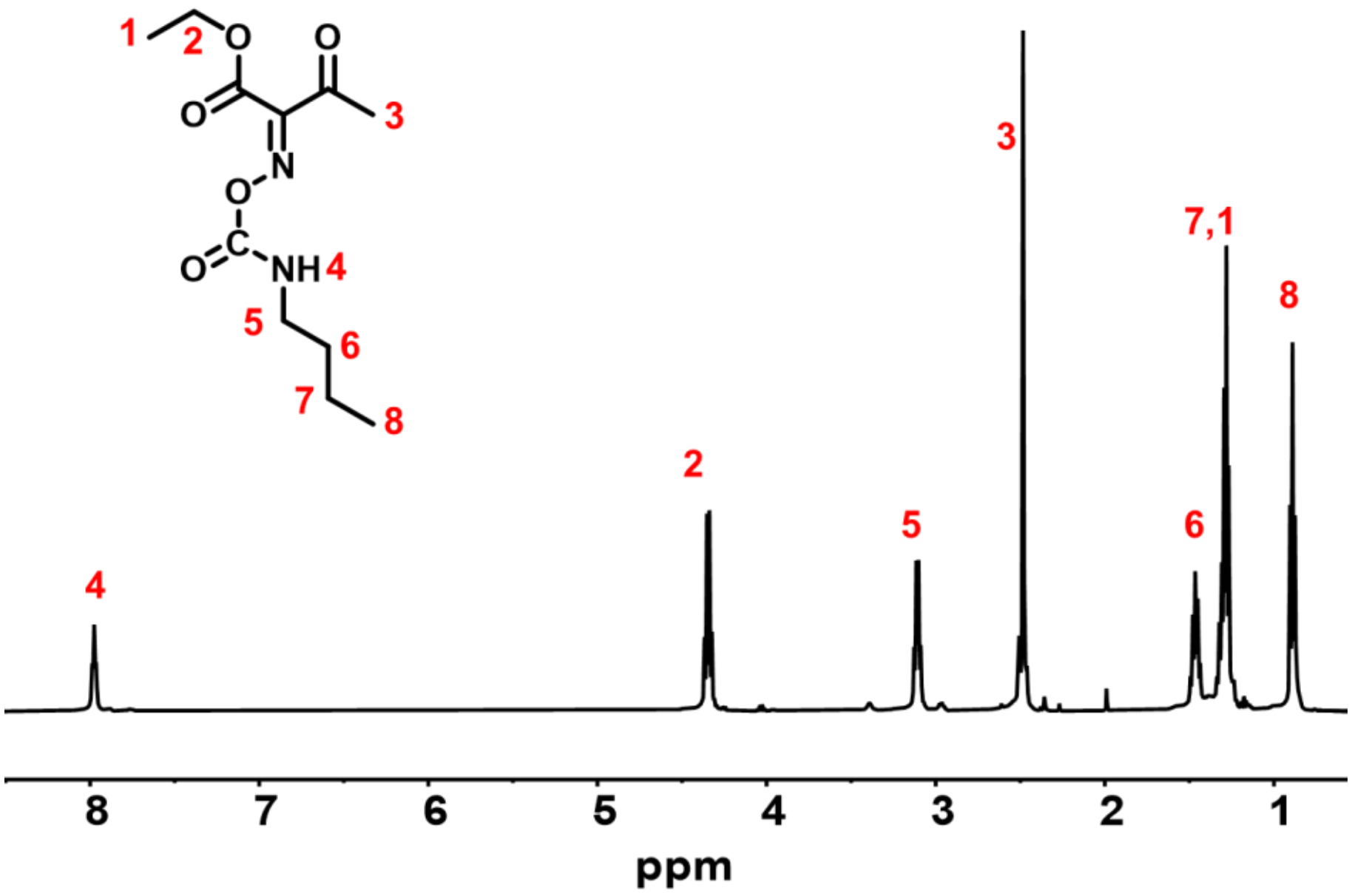

Figure S28. ^{1}H NMR (500 MHz, DMSO-d_6) spectrum of ethyl (Z)-2-(((butylcarbamoyl)oxy)imino)-3-oxobutanoate (AOU1).

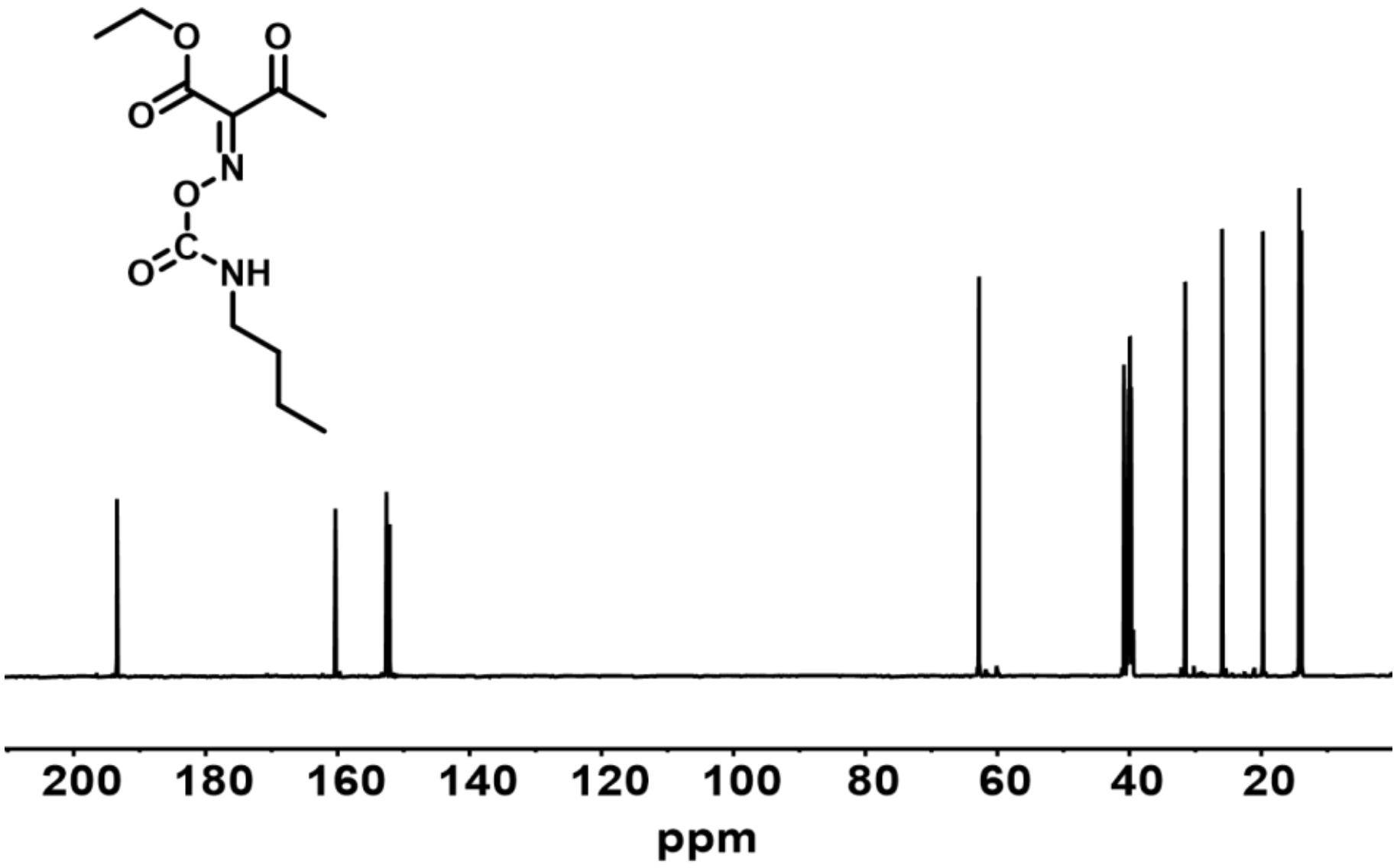

Figure S29. ^{13}C NMR (500 MHz, DMSO-d_6) spectrum of AOU1.

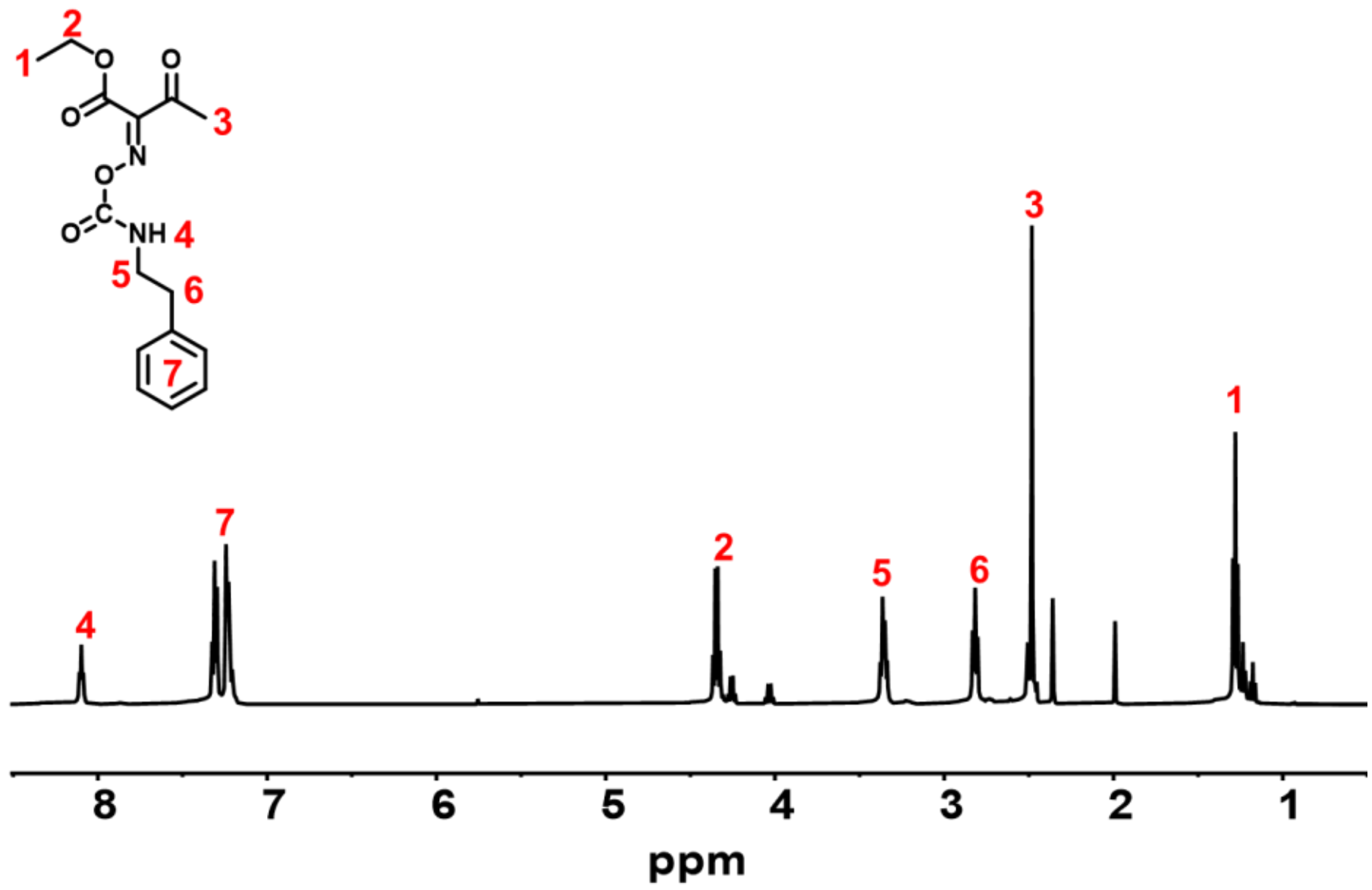

Figure S30. ^{1}H NMR (500 MHz, DMSO-d_6) spectrum of ethyl (Z)-3-oxo-2-(((phenethylcarbamoyl)oxy)imino)butanoate (AOU2).

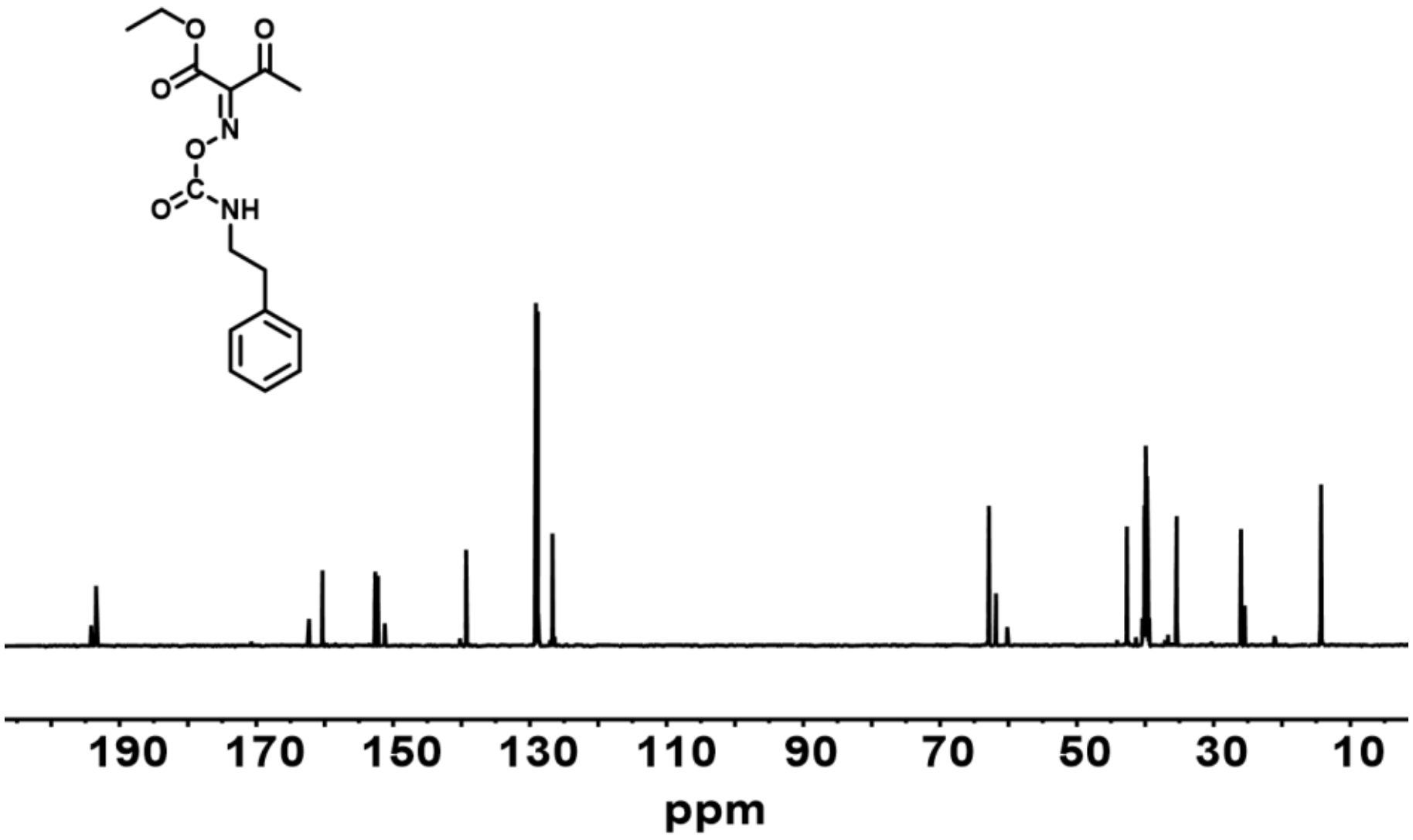

Figure S31. ^{13}C NMR (500 MHz, DMSO-d_6) spectrum of AOU2.

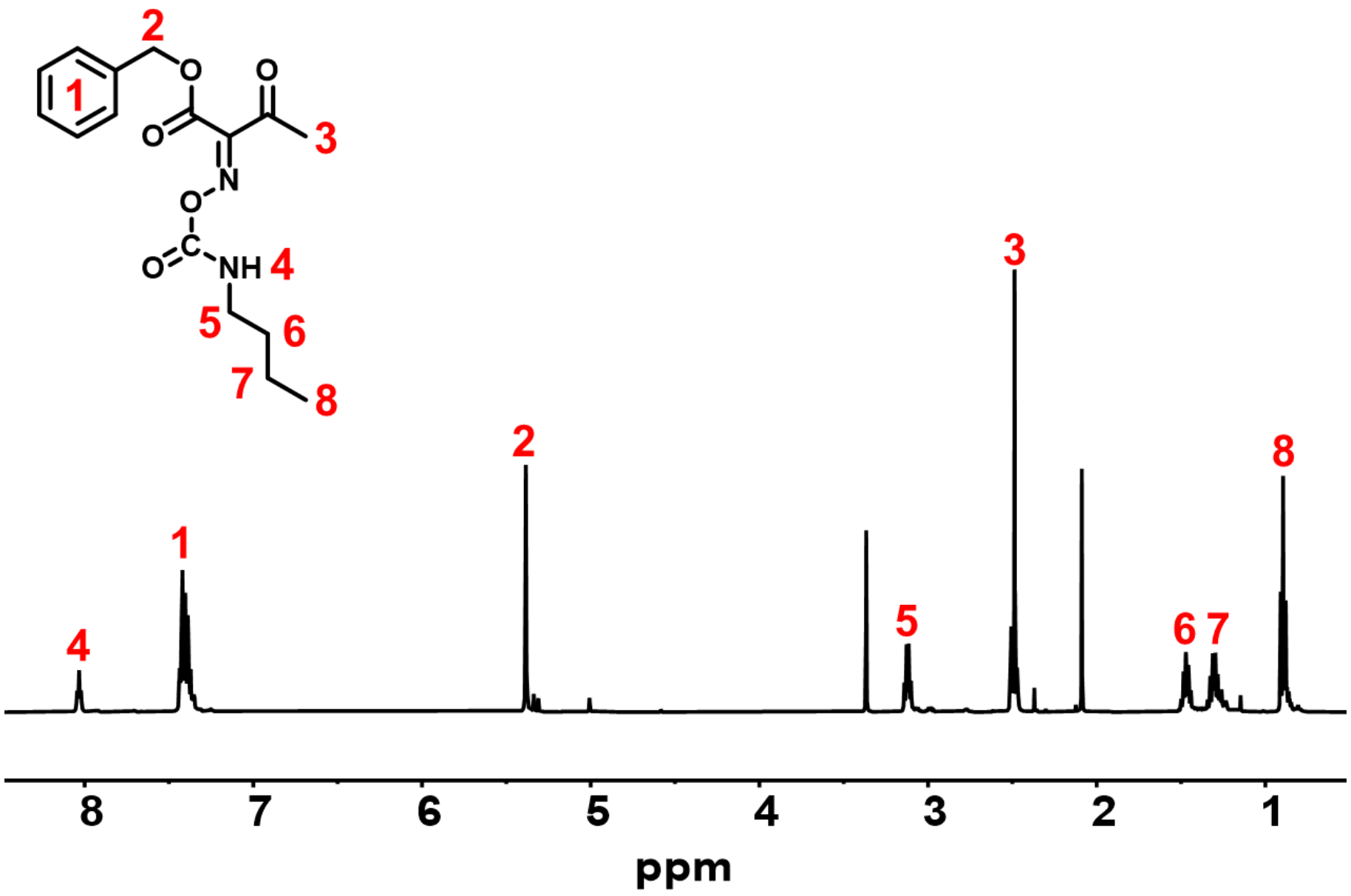

Figure S32. ^{1}H NMR (500 MHz, DMSO-d_6) spectrum of benzyl (Z)-2-(((butylcarbamoyl)oxy)imino)-3-oxobutanoate (AOU3).

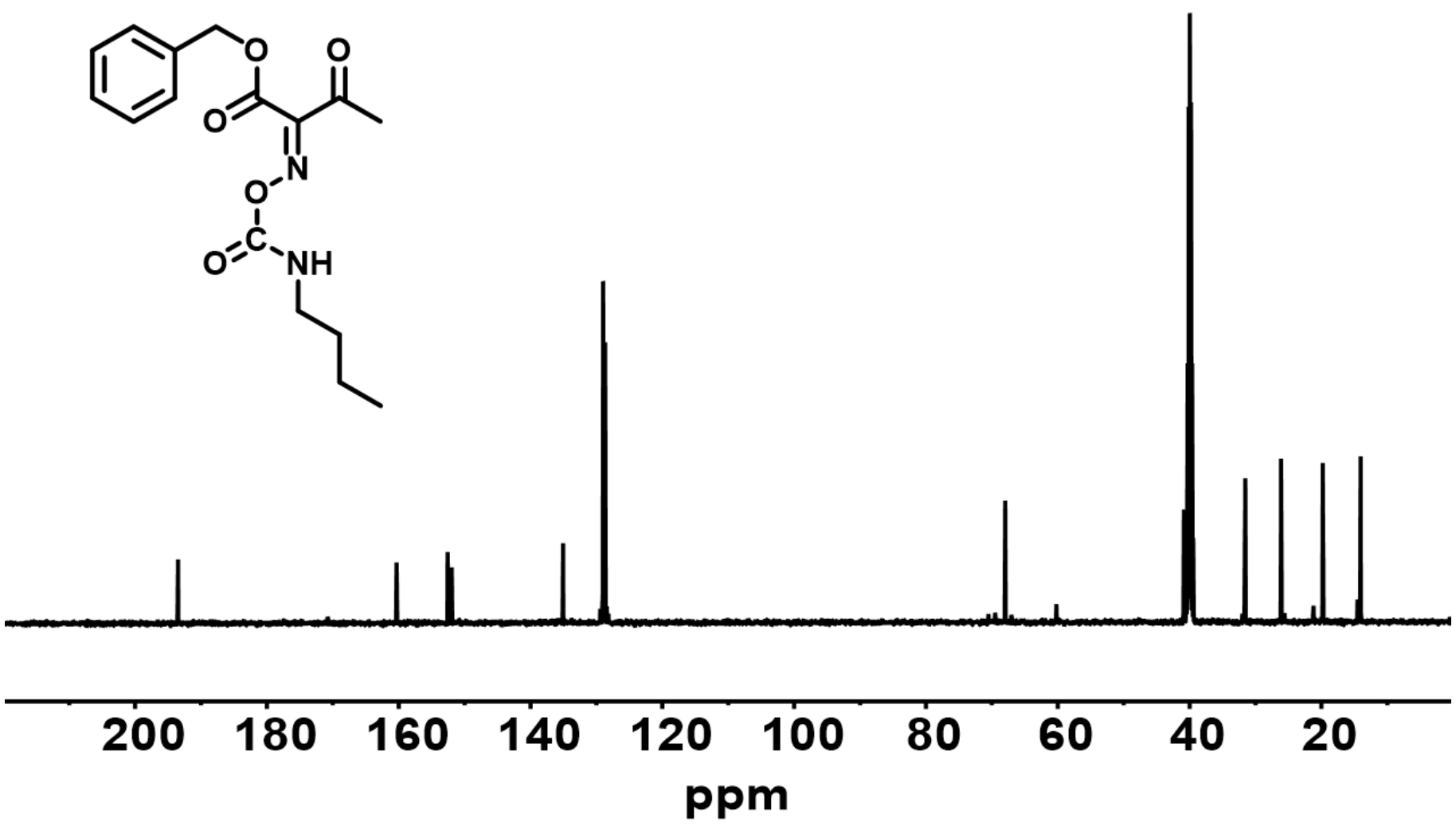

Figure S33. ^{13}C NMR (500 MHz, DMSO-d_6) spectrum of AOU3.

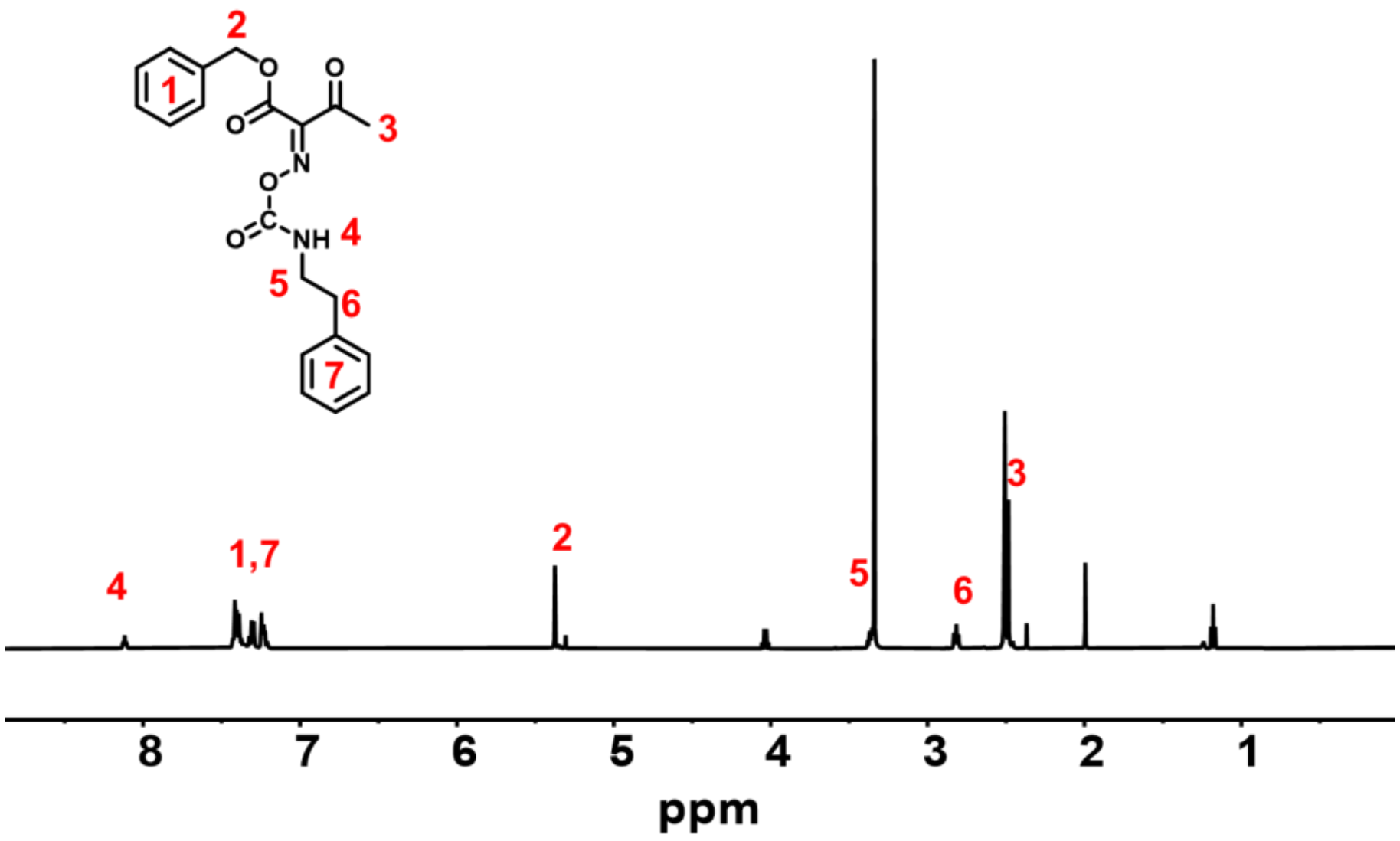

Figure S34. ^{1}H NMR (500 MHz, DMSO-d_6) spectrum of benzyl (Z)-3-oxo-2-(((phenethylcarbamoyl)oxy)imino)butanoate (AOU4).

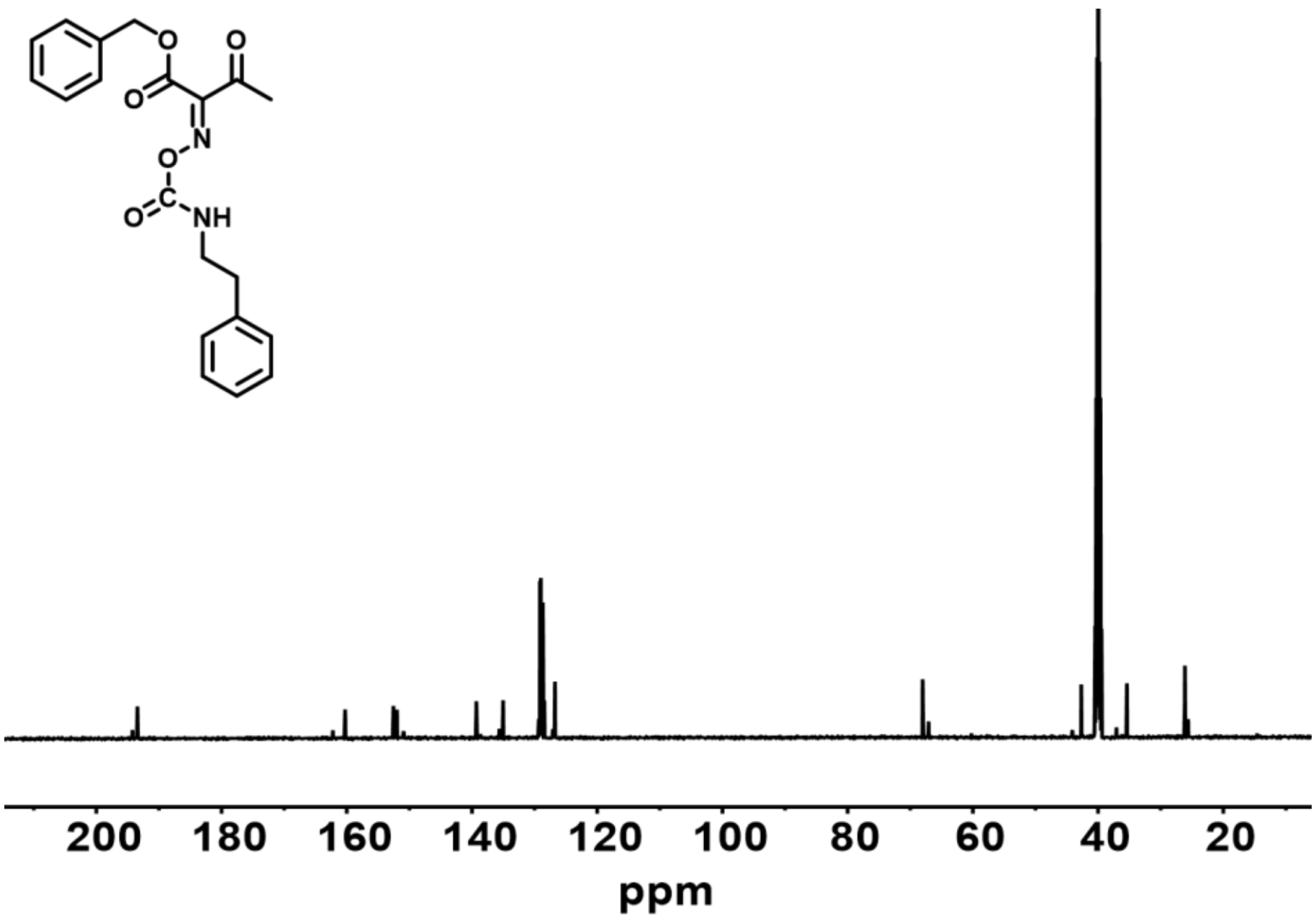

Figure S35. ^{13}C NMR (500 MHz, DMSO-d_6) spectrum of AOU4.

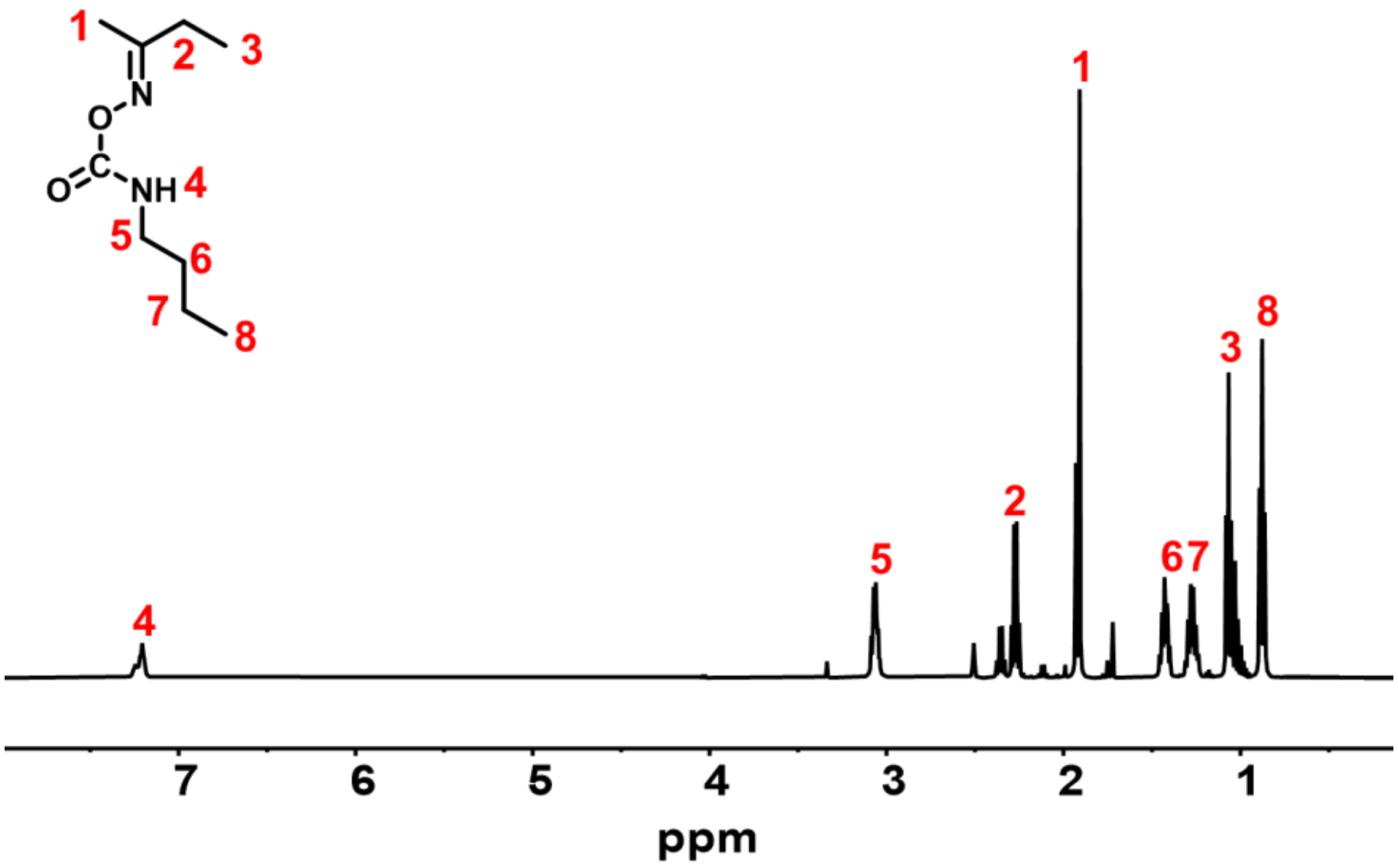

Figure S36. ^{1}H NMR (500 MHz, DMSO-d_6) spectrum of (E)-butan-2-one O-butylcarbamoyl oxime (OU1).

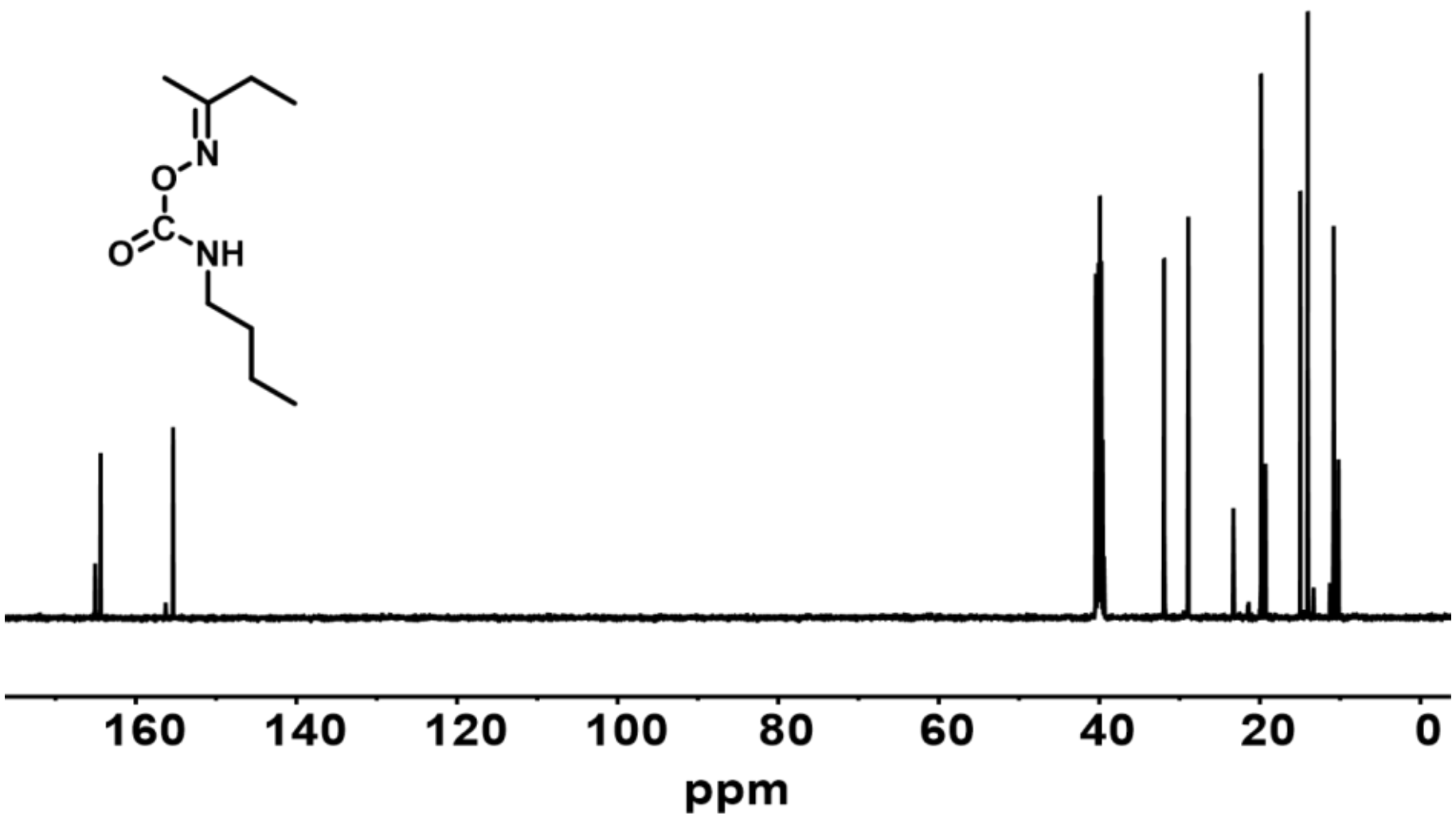

Figure S37. ^{13}C NMR (500 MHz, DMSO-d_6) spectrum of OU1.

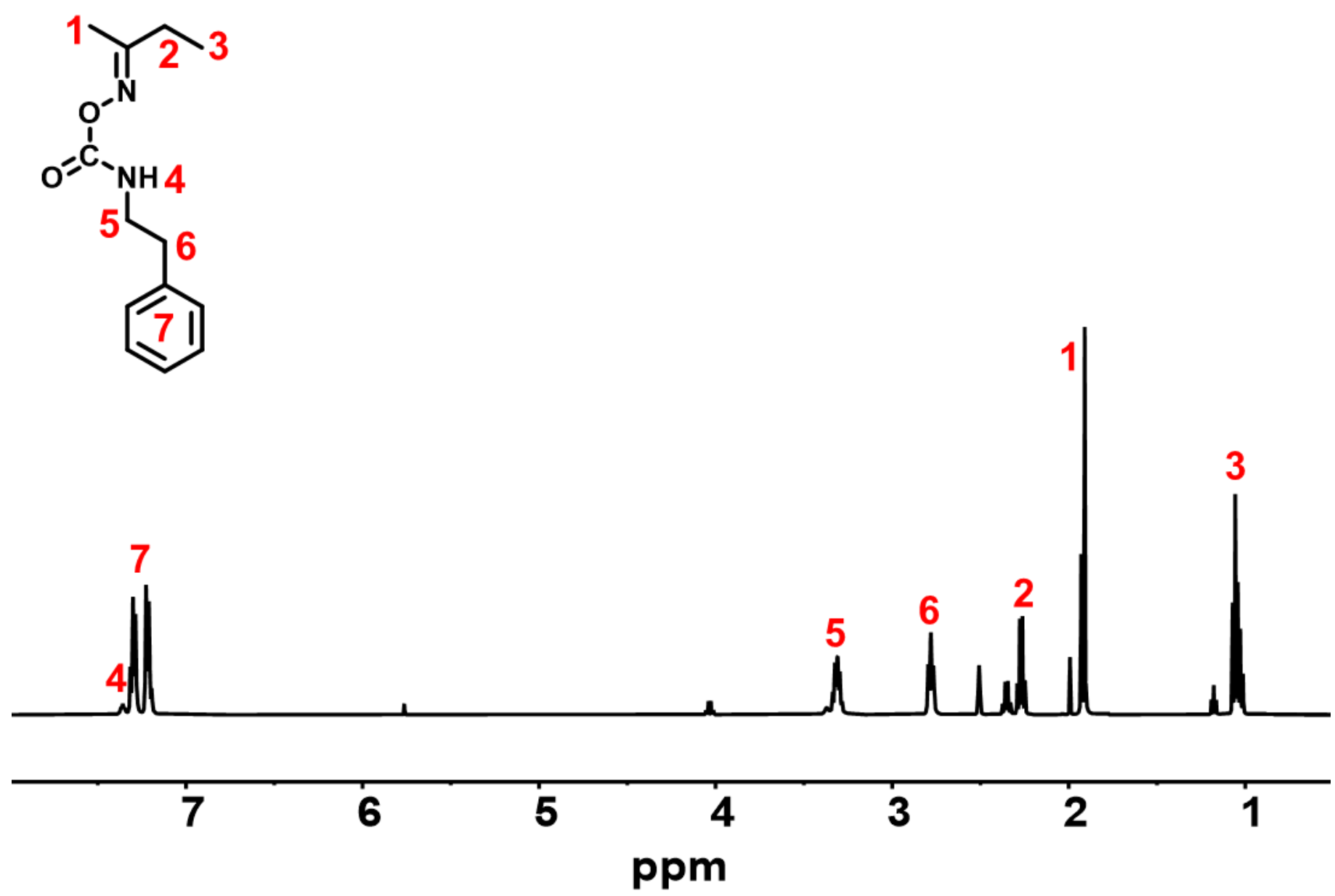

Figure S38. ^{1}H NMR (500 MHz, DMSO-d_6) spectrum of (E)-butan-2-one O-phenethylcarbamoyl oxime (OU2).

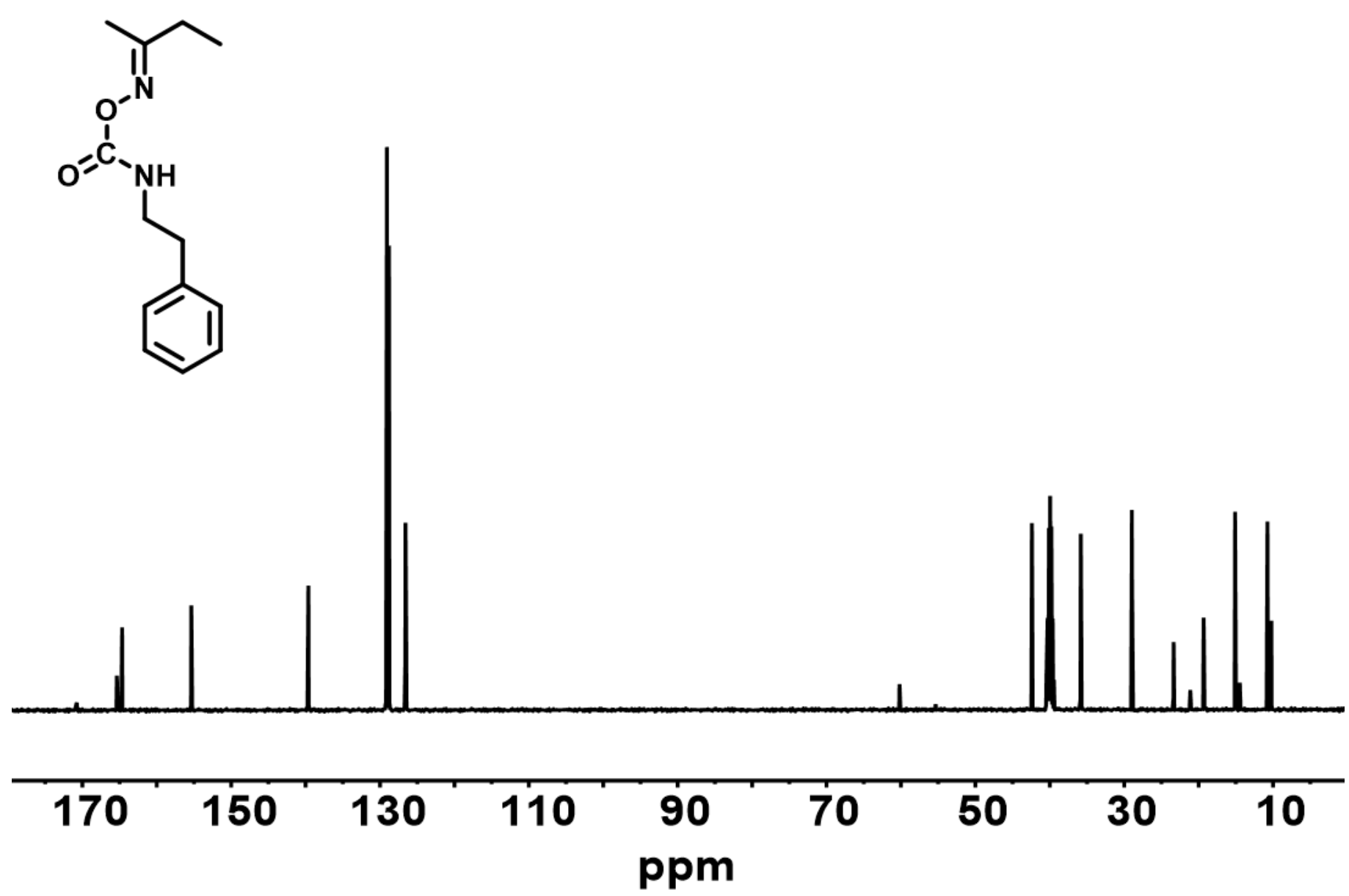

Figure S39. ^{13}C NMR (500 MHz, DMSO-d_6) spectrum of OU2.

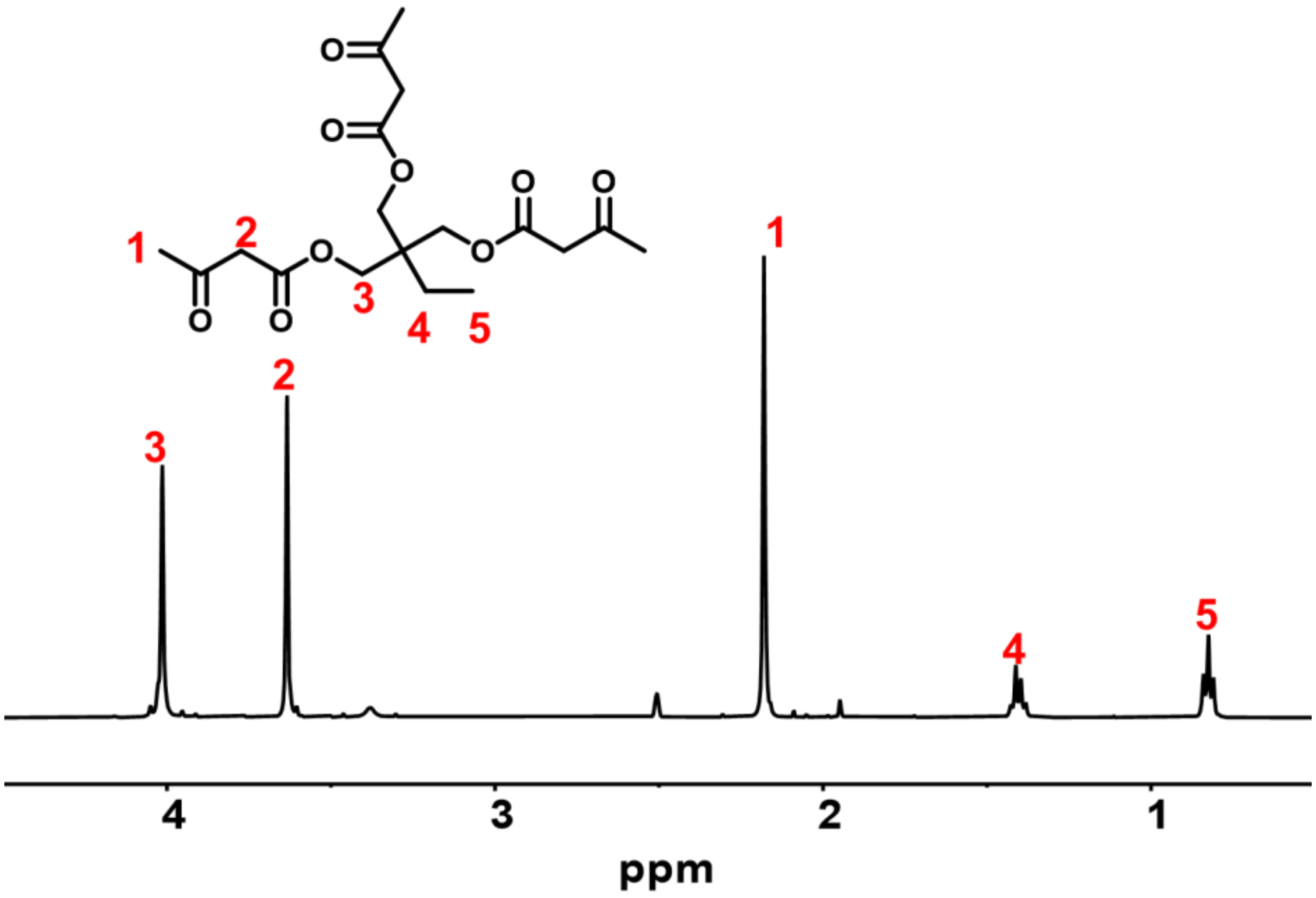

Figure S40. ¹H NMR (500 MHz, DMSO-d_6) spectrum of trimethylolpropane acetoacetates (TA).

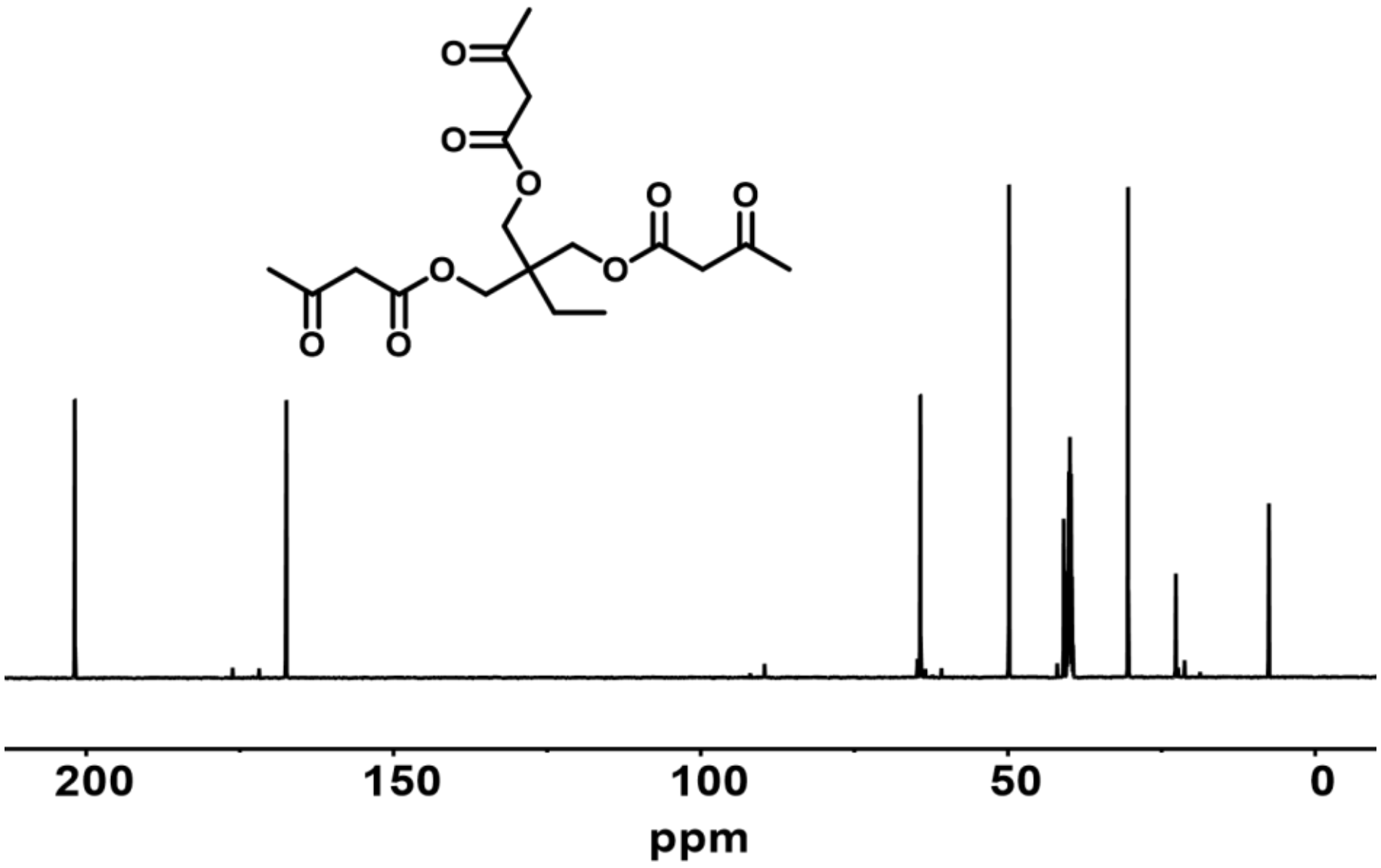

Figure S41. ¹³C NMR (500 MHz, DMSO-d_6) spectrum of TA.

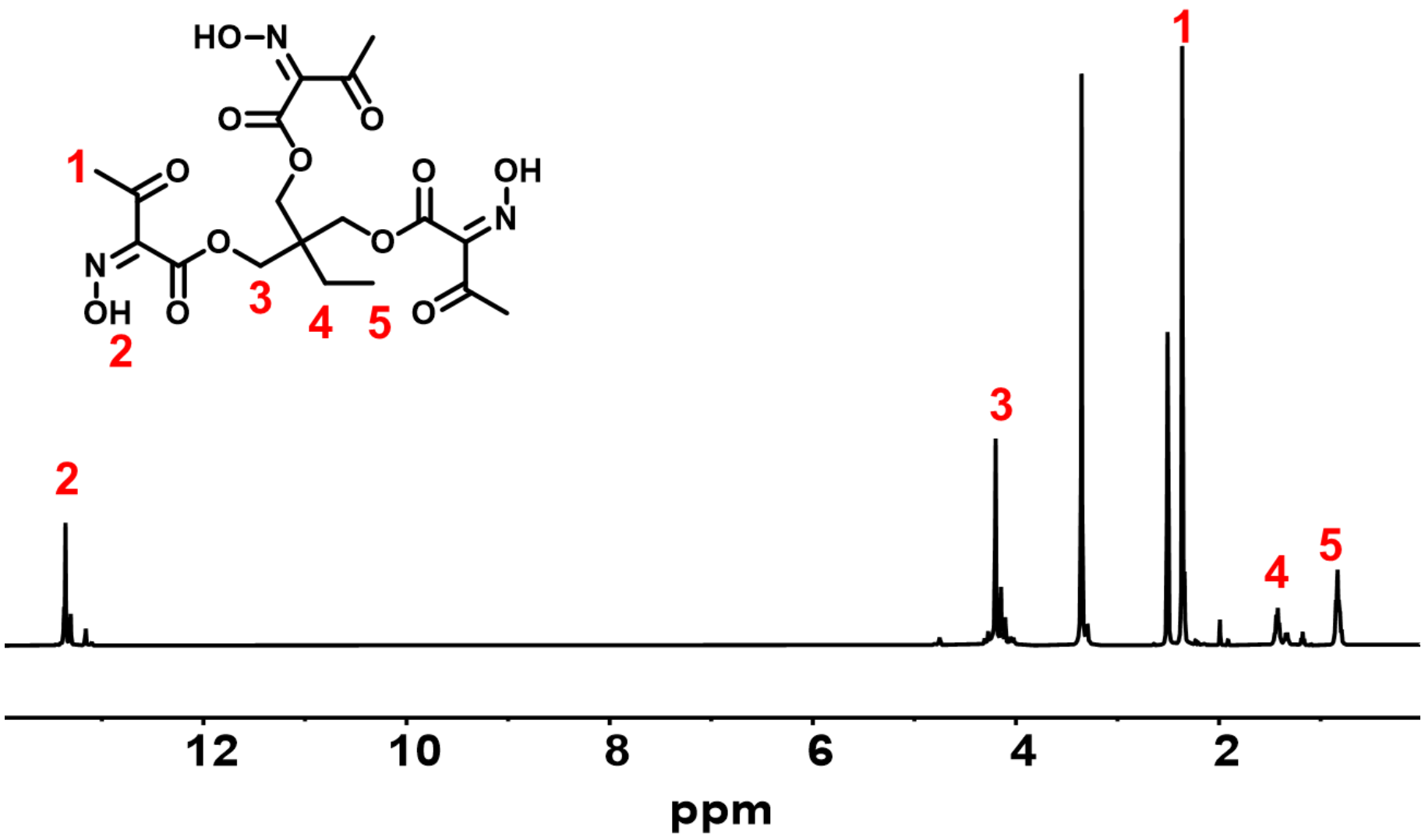

Figure S42. ^{1}H NMR (500 MHz, DMSO-d_6) spectrum of trimethylolpropane acetoacetates oxime (TAO).

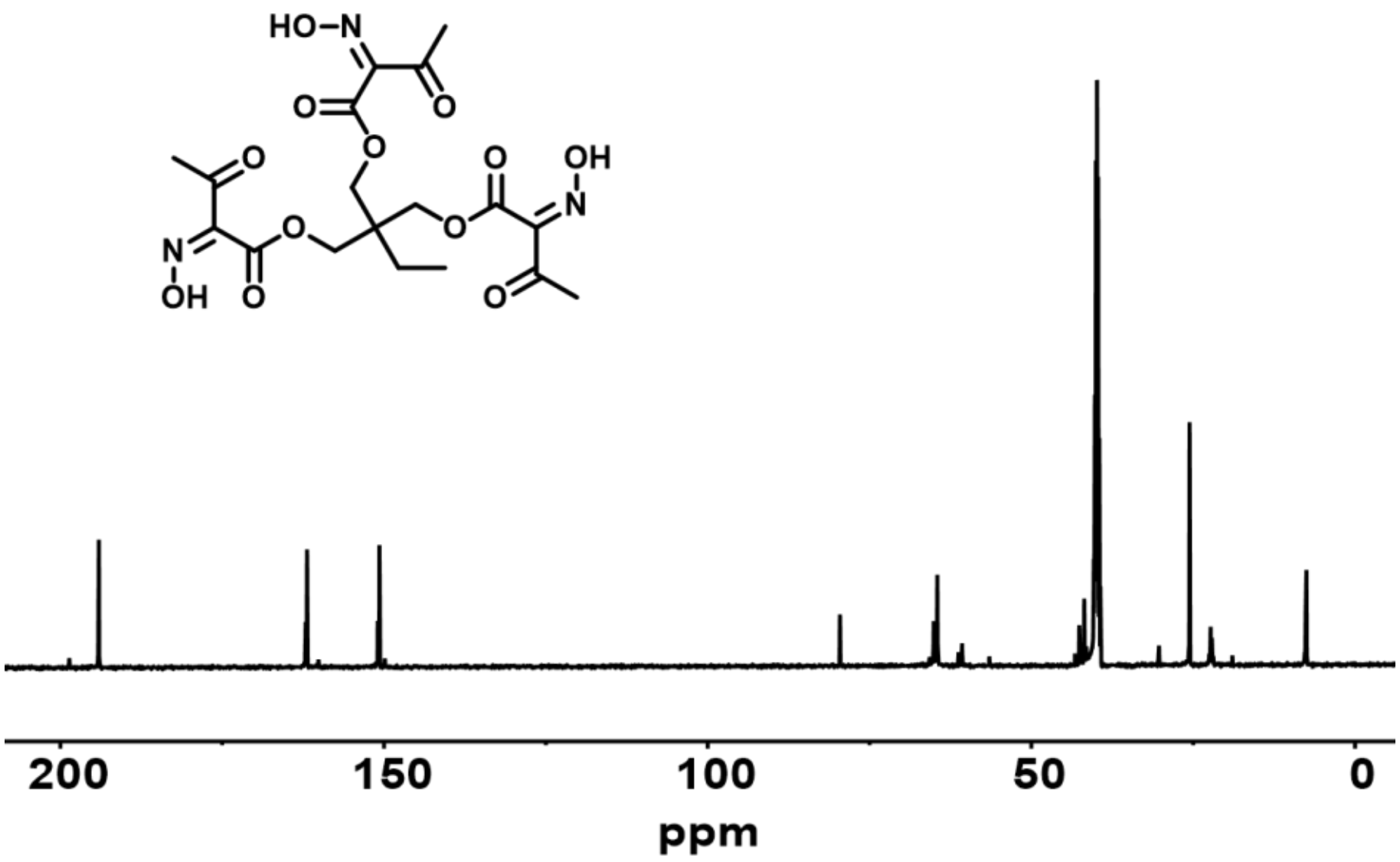

Figure S43. ^{1}H NMR (500 MHz, DMSO-d_6) spectrum of TAO.

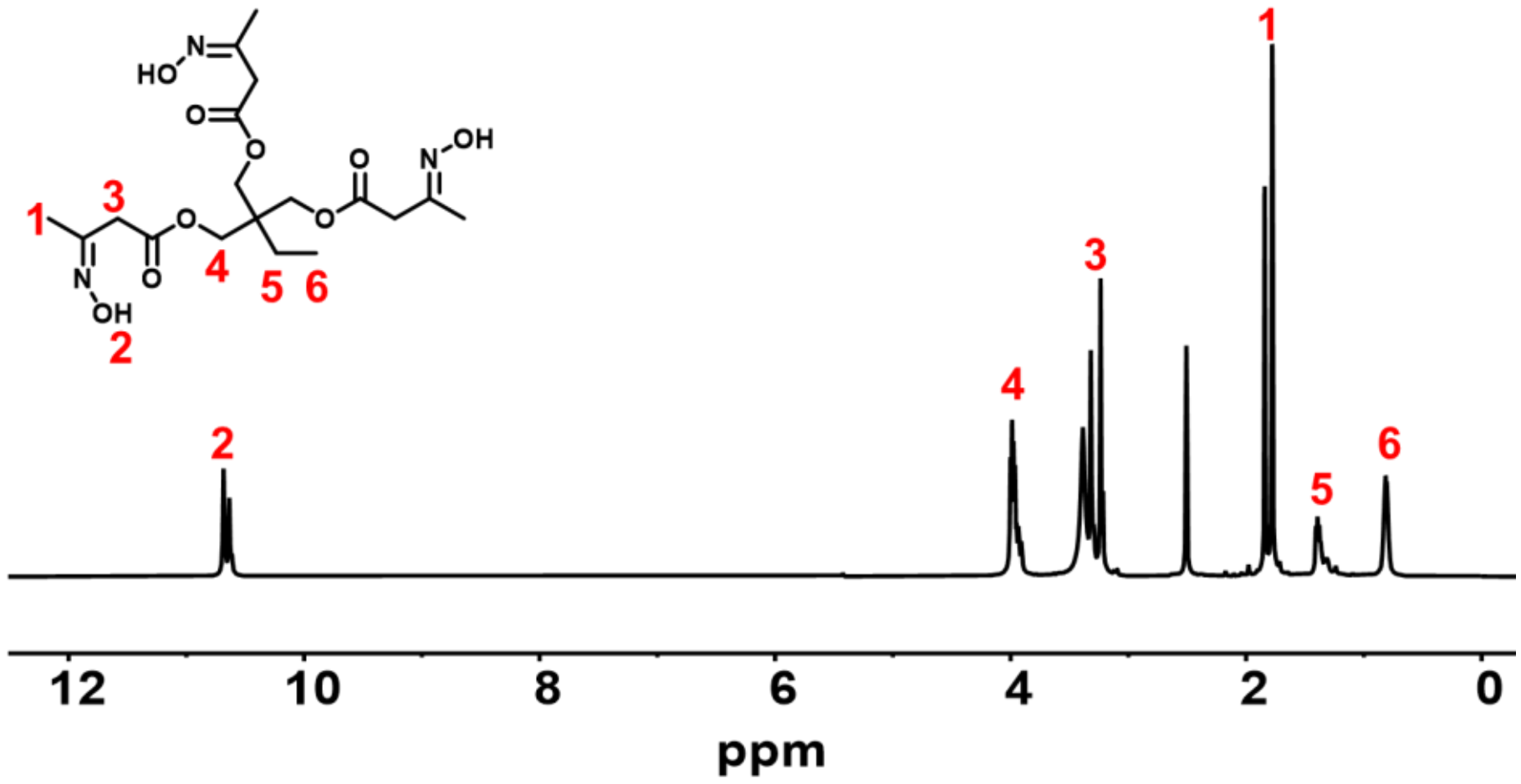

Figure S44. ^{1}H NMR (500 MHz, DMSO-d_6) spectrum of trimethylolpropane oxime (TO).

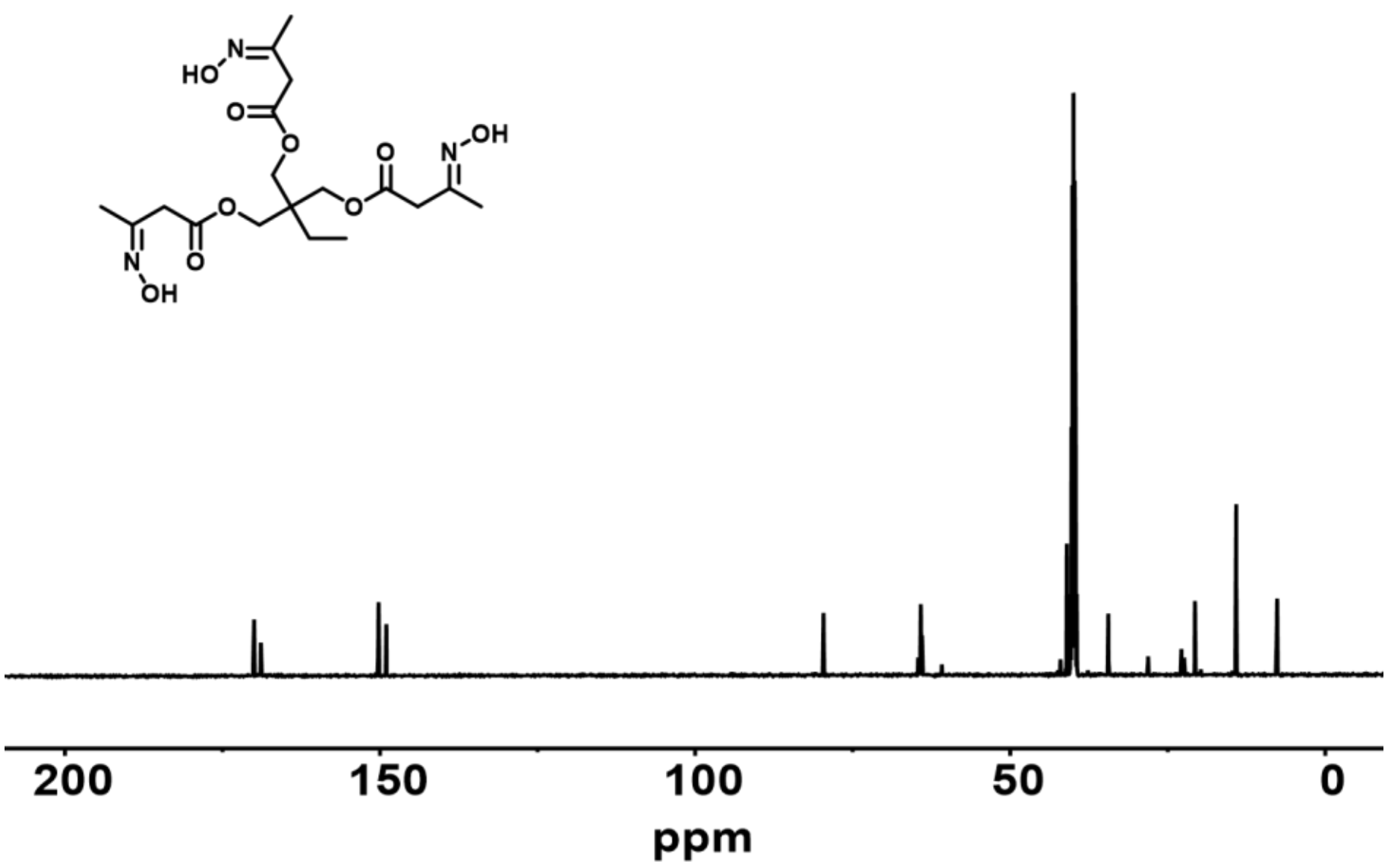

Figure S45. ^{1}H NMR (500 MHz, DMSO-d_6) spectrum of TO.

Appendix of the Cartesian coordinates (Å) in THF

OU-s

Atom	X	Y	Z
C	2.26638300	-1.21447300	0.66192400
C	1.80099300	0.01547100	-0.06955400
C	2.81231400	1.01582500	-0.55367500
N	0.57467600	0.28571700	-0.32948600
O	-0.26668200	-0.75932200	0.16681700
C	-1.60442800	-0.61249500	-0.15242000
C	-1.59050500	1.83015000	0.55511500
H	-0.98686200	2.43820500	-0.12396300
H	-0.97270800	1.54848700	1.41094100
O	-2.24779200	-1.61068900	-0.41510300
N	-2.12481500	0.64382500	-0.10595600
H	-3.10437400	0.65031700	-0.35502000
H	-2.43519800	2.42174200	0.92078500
H	2.32585900	1.84772600	-1.06876800
H	3.39543000	1.40584800	0.29072900
H	3.52059300	0.53480000	-1.24049200
H	1.93266500	-2.11726400	0.13862200
H	3.35603500	-1.22812800	0.74816000
H	1.82677600	-1.25531000	1.66504600

OU-d

Atom	X	Y	Z
C	-5.84522600	1.32616900	-0.62567500
C	-5.43246400	0.11162200	0.16119100
C	-6.48379500	-0.74285100	0.81103300
N	-4.21853700	-0.26302000	0.33447000

O	-3.33119000	0.65388700	-0.30890300
C	-1.99340700	0.34906500	-0.14550100
C	-2.36455000	-2.07177000	-0.74177400
H	-2.93184700	-2.57367000	0.04678800
H	-1.64325400	-2.77214000	-1.17201300
O	-1.22909800	1.29958300	0.02097400
N	-1.61018100	-0.93409700	-0.23062500
H	-0.60121600	-1.07206000	-0.10177000
H	-3.05950000	-1.76017000	-1.52574700
C	5.84506300	-1.32620600	0.62627700
C	5.43252700	-0.11171800	-0.16079600
C	6.48403400	0.74261400	-0.81053700
N	4.21865600	0.26297300	-0.33436000
O	3.33112800	-0.65383300	0.30890800
C	1.99339200	-0.34898600	0.14517700
C	2.36442600	2.07188900	0.74137100
H	2.93190400	2.57374800	-0.04708700
H	3.05919500	1.76032700	1.52551700
O	1.22909900	-1.29950100	-0.02139500
N	1.61017000	0.93419200	0.23010400
H	0.60123900	1.07216400	0.10098800
H	1.64303400	2.77228400	1.17141000
H	-6.93272400	1.43784100	-0.62952300
H	-5.39059600	2.22788900	-0.20050200
H	-5.48805900	1.25455100	-1.65933400
H	-7.17802600	-1.13230700	0.05497500
H	-6.03108900	-1.58015500	1.34765800
H	-7.07583200	-0.14433500	1.51547300
H	6.03148200	1.57988000	-1.34735200

H	7.17813800	1.13212100	-0.05438900
H	7.07617600	0.14398100	-1.51479000
H	5.39048700	-2.22794800	0.20109200
H	6.93255600	-1.43792700	0.63037200
H	5.48767400	-1.25447400	1.65985200

AOU-s

Atom	X	Y	Z
C	1.51074800	-0.79095100	-0.30272200
C	0.75170200	0.51459300	-0.24134900
C	1.55020900	1.78075700	-0.10371200
O	2.02547400	-1.20194500	-1.31534300
O	2.74283800	1.68744700	0.13185700
C	0.83373700	3.09905700	-0.25788700
H	0.41707700	3.18470200	-1.26804700
H	1.54146000	3.90994200	-0.07625100
H	-0.00836900	3.16764800	0.43877800
O	1.52725500	-1.39308600	0.89129000
C	2.23655600	-2.65188200	0.94366400
H	1.76963400	-3.36886000	0.26446900
H	2.15439900	-2.98233000	1.97832700
N	-0.52266400	0.61580500	-0.32539000
O	-1.12845400	-0.63522600	-0.44948200
C	-2.53450900	-0.55862200	-0.51135500
C	-2.50696800	0.65671000	1.71098900
H	-2.13939700	1.68102000	1.59439500
H	-1.67665200	0.01514900	2.01283100
O	-3.11018400	-1.16163300	-1.38700100
N	-3.11440800	0.15497100	0.48276300

H	-4.12083700	0.20160100	0.39503500
H	3.28111100	-2.50068700	0.66224800
H	-3.26320600	0.63396900	2.50027800

AOU-d

Atom	X	Y	Z
C	-5.97488700	0.92300600	0.34250500
C	-5.38193000	-0.46455100	0.25483800
C	-6.33057800	-1.63113000	0.25507500
O	-6.29612900	1.43308800	1.38955700
O	-7.52501700	-1.40167300	0.17096500
C	-5.75467600	-3.02173700	0.34987700
H	-5.04444400	-3.20231300	-0.46412300
H	-6.57003500	-3.74599400	0.30510500
H	-5.19895300	-3.13863700	1.28710400
O	-6.08884500	1.47227200	-0.87092300
C	-6.66063200	2.79976700	-0.90333300
H	-7.66766800	2.77961700	-0.48069700
H	-6.68435600	3.07387700	-1.95722800
N	-4.12653400	-0.71413200	0.20937900
O	-3.37195300	0.46071900	0.21890300
C	-1.99212300	0.22522900	0.12631500
C	-2.35988900	-1.11334400	-1.96914200
H	-2.81357100	-2.07968600	-1.72932700
H	-1.67840200	-1.23833700	-2.81426300
O	-1.27599000	0.82987500	0.91452300
N	-1.58250500	-0.58957100	-0.85143400
H	-0.56654400	-0.73496600	-0.87362400
H	-6.03273300	3.48800400	-0.33283100

H	-3.14735200	-0.41271900	-2.25580500
C	5.97523000	-0.92243000	-0.34275300
C	5.38152900	0.46483000	-0.25545300
C	6.32951200	1.63195300	-0.25669800
O	6.29618400	-1.43286800	-1.38972000
O	7.52413000	1.40321200	-0.17320200
C	5.75274700	3.02220400	-0.35152700
H	5.19570000	3.13834500	-1.28805600
H	6.56777800	3.74691700	-0.30815700
H	5.04351700	3.20281700	0.46334800
O	6.09012400	-1.47102600	0.87089300
C	6.66268200	-2.79818100	0.90366900
H	6.03487900	-3.48706000	0.33383700
H	6.68710800	-3.07175000	1.95769000
N	4.12601500	0.71372400	-0.20949400
O	3.37210100	-0.46156400	-0.21806200
C	1.99218200	-0.22679500	-0.12492600
C	2.36024100	1.11283900	1.96979500
H	2.81316300	2.07940800	1.72942700
H	3.14831500	0.41280600	2.25623500
O	1.27600900	-0.83216400	-0.91254300
N	1.58260000	0.58820100	0.85266900
H	0.56659800	0.73319200	0.87518900
H	7.66948700	-2.77766700	0.48050100
H	1.67912800	1.23764900	2.81525400

AO

Atom	X	Y	Z
C	3.62247800	-0.86730300	0.30011400

H	4.66505500	-0.53122000	0.29502900
H	3.49600400	-1.61468500	-0.48933700
C	0.38258700	0.71118300	-0.11521300
C	-1.00354400	0.15558300	-0.03807200
C	-1.29691800	-1.31961200	-0.15084300
O	0.58126500	1.91843300	-0.28539600
O	-0.52504300	-2.04992200	-0.75436300
C	-2.57222600	-1.82712900	0.47425700
H	-2.56902200	-1.62781700	1.55219900
H	-2.65596900	-2.90009800	0.29263000
H	-3.43988200	-1.30626200	0.05773100
O	1.34108200	-0.17073100	0.08235500
C	2.71856200	0.32412300	0.06937200
H	2.81041200	1.07905100	0.85453700
H	2.89207300	0.80012100	-0.89892400
H	3.41588000	-1.33653200	1.26729000
N	-2.05722600	0.90456900	0.10069100
O	-1.93949000	2.24731700	0.10577200
H	-0.98211100	2.48361800	-0.06062500

AOU-int1

Atom	X	Y	Z
C	1.76640400	3.91272900	-0.50550000
H	1.15097300	4.79159400	-0.72653800
H	2.58348800	3.87177700	-1.23276200
C	1.19391700	0.33847500	-0.29282100
C	2.10821800	-0.81007400	-0.01804000
C	3.49395200	-0.63930500	0.55117600
O	0.00384100	0.15510000	-0.56306800

O	3.77071900	0.35506700	1.20563100
C	4.48953600	-1.74690600	0.30687600
H	4.13122900	-2.68687300	0.73838800
H	5.44401200	-1.47024900	0.75828800
H	4.61575500	-1.91798100	-0.76803600
O	1.76367300	1.52751700	-0.28372500
C	0.90378800	2.67225400	-0.58668600
H	0.08955200	2.67989700	0.14247500
H	0.48061100	2.52003700	-1.58296700
H	2.19103100	4.03064700	0.49626700
N	1.76728500	-2.04860100	-0.22586500
O	0.52439700	-2.35973600	-0.63507600
H	-0.02584400	-1.52602400	-0.68823100
C	-2.77732500	-2.66873200	0.09274600
C	-3.04193200	-0.39550900	-0.91473200
H	-4.02666800	-0.43680200	-1.39485000
H	-2.34137300	0.04549600	-1.62662700
C	-3.09086000	0.44424300	0.36249100
H	-3.84370800	0.02476000	1.04404200
H	-2.11885600	0.37281000	0.86334700
C	-3.41097100	1.91277900	0.06630600
H	-4.38098900	1.98325200	-0.44545900
H	-2.66041800	2.30876200	-0.63183500
C	-3.43407400	2.77333500	1.33319700
H	-3.66053000	3.82001100	1.09906500
H	-4.19297900	2.41520600	2.03996300
H	-2.46319600	2.74494200	1.84388200
O	-2.86000400	-3.63736300	0.77456100
N	-2.59543000	-1.75935300	-0.67200000

AOU-TS1

Atom	X	Y	Z
C	1.01341700	3.76886300	0.55704600
H	0.45804400	4.70944300	0.46372200
H	2.03951600	4.00125800	0.85906400
C	1.24392900	0.73044200	-0.21601800
C	2.17671100	-0.43194200	-0.17542300
C	3.53067000	-0.29714400	0.38418100
O	0.06522200	0.67847500	0.13273600
O	3.88125400	0.73390700	0.96682900
C	4.48015100	-1.47162200	0.25160200
H	4.04144500	-2.38180400	0.67133400
H	5.41017500	-1.23107800	0.77146400
H	4.68921100	-1.67772300	-0.80397000
O	1.80366600	1.83034300	-0.72402800
C	0.99647500	3.04181100	-0.77718700
H	-0.01667600	2.77258300	-1.08325200
H	1.46998000	3.63160200	-1.56363900
H	0.54253900	3.16698500	1.33983200
N	1.81156400	-1.63337500	-0.59368900
O	0.60779000	-1.77914300	-1.05745000
H	-0.68946300	-1.04256100	-0.17433600
C	-1.40207000	-2.83708100	0.05804400
C	-2.67377300	-0.90421300	0.86819900
H	-2.23608100	-0.35290800	1.70357900
H	-3.34664900	-1.66927200	1.26112500
C	-3.37014900	0.02430700	-0.11894900
H	-2.62997700	0.71713700	-0.53653700

H	-3.77421900	-0.56620700	-0.95073200
C	-4.49572200	0.81211200	0.56366500
H	-4.08008100	1.38290700	1.40503500
H	-5.22726300	0.11208700	0.99000400
C	-5.20104700	1.76568800	-0.40552700
H	-6.00003100	2.31993500	0.09961200
H	-4.49581700	2.49527100	-0.82205000
H	-5.64914700	1.21639900	-1.24261800
O	-1.39637900	-3.98974800	-0.01748900
N	-1.52434500	-1.59549100	0.20365800

AOU1

Atom	X	Y	Z
C	-2.43552500	-0.99554400	-0.31235200
C	-1.59033200	0.24163500	-0.16753800
C	-2.25453700	1.57278400	0.17289900
O	-3.67268100	-0.73376400	0.11497800
O	-2.76253600	2.20834400	-0.73189900
C	-2.23065000	1.99093200	1.61240200
H	-2.72897800	1.22785700	2.22289000
H	-2.73124300	2.95379400	1.73408500
H	-1.19218600	2.05852400	1.95768700
O	-2.04266700	-2.06141300	-0.73944000
N	-0.33786800	0.09222600	-0.37534300
O	0.35017000	1.29380200	-0.19528300
H	1.54575900	-0.74328400	-0.89440700
C	-4.64614400	-1.81982500	0.04697400
H	-4.72080900	-2.13456000	-0.99726200
H	-4.25963800	-2.65378900	0.63872900

C	-5.95563700	-1.28499100	0.58545800
H	-6.31024100	-0.43967400	-0.01282800
H	-6.71067900	-2.07752300	0.54528100
H	-5.85029700	-0.96205300	1.62624800
C	1.73860000	1.20971800	-0.41061500
C	3.63217100	-0.22135600	-1.00999200
H	4.08324300	0.69951900	-1.38946200
H	3.72790600	-0.98509200	-1.78797600
C	4.33145300	-0.67006800	0.27622800
H	4.19142500	0.10210700	1.04414600
H	3.84827600	-1.58392500	0.64893500
C	5.82726500	-0.92417700	0.06232900
H	6.29942500	-0.00723300	-0.31736500
H	5.95882000	-1.68690800	-0.71831000
C	6.53789500	-1.37379100	1.34262700
H	7.60554100	-1.54813600	1.16520700
H	6.44768000	-0.61440400	2.12947600
H	6.10453600	-2.30583100	1.72691900
O	2.36729800	2.23432500	-0.21179600
N	2.20178600	0.02468200	-0.82537100

BO

Atom	X	Y	Z
C	0.34837900	1.51305900	-0.08039400
C	0.10385100	0.03608300	-0.23350000
C	-1.27689900	-0.45977500	-0.57895400
C	-2.29738800	-0.17773000	0.53714200
H	-2.01295900	-0.69235800	1.46192400
H	-3.29140600	-0.53052200	0.24111000

H	-2.37269700	0.89377100	0.75424600
N	1.00833900	-0.85988500	-0.07067300
O	2.25995200	-0.28010500	0.27683200
H	0.59008900	1.75936300	0.96054500
H	1.20436700	1.82482400	-0.68842600
H	-0.53150700	2.08788800	-0.37947500
H	-1.60334100	0.03669400	-1.50350300
H	-1.22855800	-1.53465500	-0.78135600
H	2.84037400	-1.05479500	0.34922700

OU-int1

Atom	X	Y	Z
C	2.59586500	-2.05450100	1.03277500
C	2.07453000	-1.26747600	-0.13920700
C	3.02016100	-0.42873500	-0.96057600
C	3.56182000	0.77538900	-0.17056800
H	2.74393000	1.44029500	0.12857000
H	4.26337900	1.35093400	-0.78452800
H	4.09040600	0.45913400	0.73592200
C	-3.53760000	-0.90031500	-0.02607700
C	-1.80629800	0.66189500	-0.89507800
H	-2.54498300	1.27217800	-1.42507500
H	-1.02935400	0.36252500	-1.60023300
C	-1.18016300	1.43540900	0.26584400
H	-0.53580400	0.75509400	0.83429100
H	-1.97170300	1.77983100	0.94492700
C	-0.35403600	2.62649000	-0.23045400
H	-0.99217800	3.30076000	-0.81872700
H	0.42553900	2.26024800	-0.91264800

N	0.83915500	-1.23234900	-0.48397100
O	0.02754900	-2.02261800	0.36689300
O	-4.55547200	-1.34580400	0.38070500
N	-2.44827800	-0.57532300	-0.43494000
C	0.29482200	3.40516800	0.91776900
H	0.95767100	2.75746500	1.50522500
H	0.89167200	4.24430900	0.54209500
H	-0.46424400	3.81007100	1.59867300
H	-0.87500600	-1.78876100	0.07183300
H	2.13777700	-1.70693200	1.96634100
H	2.33689000	-3.11416900	0.92811000
H	3.68151100	-1.96180900	1.11624500
H	3.85919700	-1.05820000	-1.28772500
H	2.49793400	-0.08183700	-1.85827600

OU-TS1

Atom	X	Y	Z
C	-3.12555200	1.12893100	1.04236700
C	-3.04420100	-0.01485300	0.06488800
C	-4.27709300	-0.41314800	-0.70488100
C	-5.39296600	-0.94458200	0.21160700
H	-5.06148800	-1.84238800	0.74552900
H	-6.27747100	-1.20548500	-0.37966200
H	-5.69480200	-0.19894400	0.95568600
C	0.31470500	0.79387100	-0.39030100
C	2.45761200	-0.26031700	-0.88397200
H	2.59932700	0.57960400	-1.57340400
H	2.55562600	-1.18576100	-1.46036300
C	3.48941800	-0.21792300	0.24379100

H	3.29096500	-1.04406500	0.93963300
H	3.36216600	0.71392400	0.81101700
C	4.92521800	-0.31518300	-0.28389900
H	5.11159400	0.50902100	-0.98661400
H	5.03876100	-1.24503400	-0.85865300
N	-1.98590300	-0.71529800	-0.14589200
O	-0.89222000	-0.29373200	0.64220400
O	-0.02120700	1.88810400	-0.68436900
N	1.08281300	-0.22948100	-0.36019300
C	5.96671300	-0.27646800	0.83876700
H	5.82185500	-1.10894700	1.53867500
H	6.98477400	-0.34723500	0.43863600
H	5.89446900	0.65709700	1.41069700
H	0.19455900	-0.84582700	0.26420000
H	-3.06125100	0.75408900	2.07134300
H	-2.29517400	1.82470600	0.89958400
H	-4.06943700	1.66866200	0.92993100
H	-4.64493600	0.46591900	-1.25230900
H	-4.00361200	-1.17283100	-1.44422200

OU1

Atom	X	Y	Z
C	3.58864200	1.16475500	0.83880200
C	2.89645100	-0.10562000	0.43078800
C	3.65984300	-1.40444800	0.42899600
C	4.78702200	-1.41276900	-0.61822800
H	4.37953300	-1.29480700	-1.62833700
H	5.33014000	-2.36273200	-0.57666500
H	5.50738100	-0.60598400	-0.44448600

C	-0.26301700	1.12888400	-0.31970800
C	-2.20440600	-0.18853200	-1.03838300
H	-2.28741800	-1.02359600	-1.74192100
H	-2.51680000	0.71928900	-1.56217800
C	-3.09506500	-0.42812700	0.18476100
H	-2.96569800	0.40798100	0.88446900
H	-2.75620000	-1.33532600	0.70452800
C	-4.57386600	-0.56751900	-0.19061200
H	-4.69183400	-1.39524900	-0.90464200
H	-4.90138100	0.34182400	-0.71399300
N	1.67217100	-0.17844600	0.05333000
O	1.06049000	1.11836600	0.07220500
O	-0.83704100	2.21215100	-0.30345900
N	-0.79150100	-0.04787700	-0.70025800
H	-0.20643200	-0.86805100	-0.60225900
C	-5.47254200	-0.80939900	1.02611600
H	-5.39675300	0.01932300	1.74129200
H	-6.52462000	-0.90435900	0.73265400
H	-5.18568400	-1.72968100	1.55063900
H	4.08848400	-1.55101000	1.42961900
H	3.67676900	1.84436000	-0.01672900
H	3.00723700	1.68811000	1.60432300
H	4.58830500	0.95519100	1.22534400
H	2.96431600	-2.22852100	0.24167600

AO-int1

Atom	X	Y	Z
C	-0.47688100	-0.66832600	-0.00836900
C	0.89088800	-0.24899500	-0.01385700

C	1.30548200	1.17505600	-0.02292200
O	-1.46455000	0.16470000	0.00768100
O	0.46983000	2.07171400	-0.14021500
C	2.77147800	1.50804700	0.11724900
H	3.19958200	1.03975100	1.00931900
H	2.87180400	2.59483400	0.16914600
H	3.33794100	1.11653500	-0.73422400
O	-0.76889300	-1.94987100	-0.00829400
N	1.68230100	-1.36596500	-0.01238300
O	2.92539400	-1.28429200	-0.02116700
C	-2.84735900	-0.34734300	0.02537700
H	-2.95817500	-0.96493100	0.91913500
H	-2.97961200	-0.96775000	-0.86339900
C	-3.75623400	0.86013700	0.03401900
H	-3.60348200	1.47334200	-0.85914200
H	-4.79647600	0.51740000	0.04602400
H	-3.58379000	1.47582700	0.92191900
H	0.11761100	-2.41271400	-0.01511200

AO-TS1

Atom	X	Y	Z
C	-0.50939600	-0.64181800	-0.00003800
C	0.87978700	-0.18969500	-0.00007300
C	1.34615400	1.20853000	-0.00008900
O	-1.50381000	0.18623500	0.00006800
O	0.53688000	2.13555700	-0.00039500
C	2.83347900	1.46143600	0.00027200
H	3.30397800	1.00169900	0.87623300
H	3.00446000	2.54005000	0.00045900

H	3.30432400	1.00197000	-0.87564800
O	-0.67703100	-1.91248100	-0.00007700
N	1.60740100	-1.32466600	-0.00011200
O	2.84875000	-1.41778900	-0.00015600
C	-2.87005900	-0.36827900	0.00012500
H	-2.96975000	-0.99226200	0.89081100
H	-2.96989000	-0.99207800	-0.89067600
C	-3.82019600	0.80700900	0.00032000
H	-3.67924000	1.42699300	-0.89024700
H	-4.84831300	0.42927500	0.00034900
H	-3.67911600	1.42679800	0.89100300
H	0.48481400	-2.16505800	-0.00011800

AO1

Atom	X	Y	Z
C	-0.52482000	-0.82693400	-0.07253100
C	0.86306600	-0.28276400	-0.06126900
C	1.23082600	1.16373600	-0.14515700
O	-1.45815900	0.10252500	0.06837200
O	0.49328100	1.93212900	-0.74824500
C	2.49986000	1.63311600	0.51942800
H	2.69500700	1.09976100	1.45453600
H	2.42026100	2.70856100	0.69540600
H	3.35116800	1.43770600	-0.14241900
O	-0.74168400	-2.03108000	-0.15852400
N	1.77158300	-1.24102800	0.01489800
O	3.02621300	-1.15124800	0.03541300
H	1.36859200	-2.19480700	0.03510200
C	-2.84425000	-0.35480100	0.08951500

H	-3.03460500	-0.89494800	-0.84163600
H	-2.95848200	-1.04884400	0.92654200
C	-3.71806000	0.87295500	0.23368000
H	-3.57345400	1.55840600	-0.60732300
H	-4.76929600	0.56534800	0.25303100
H	-3.49721500	1.40554900	1.16435000

BO-TS1

Atom	X	Y	Z
C	-0.12940400	0.38990600	-0.00804000
C	1.36131600	0.60388400	-0.01323500
C	2.21488400	-0.66438700	0.01320000
H	2.01825700	-1.29473900	-0.86038100
C	-1.05430900	1.56808300	0.00788100
H	-0.98160300	2.08542400	0.97288600
H	-0.76468400	2.28319300	-0.77005300
H	-2.08780800	1.25247000	-0.14689100
N	-0.59194400	-0.80344500	-0.01471700
O	-1.99053600	-1.12378800	0.00372600
H	-0.96771900	-1.89082000	0.01147600
H	3.27680600	-0.39792700	0.00958100
H	2.01523800	-1.25969700	0.91047200
H	1.60228800	1.20730200	-0.90020600
H	1.60219700	1.24429000	0.84749300

BO1

Atom	X	Y	Z
C	-0.36847700	1.56413300	0.06352500
C	-0.11444600	0.10277400	0.22480500

C	1.23625600	-0.44489400	0.57977800
C	2.28421200	-0.19865100	-0.52107900
H	1.99789400	-0.70162600	-1.45123900
H	3.25963400	-0.58470300	-0.20636700
H	2.39680800	0.87019700	-0.73259100
N	-1.10572500	-0.72003500	0.03062800
O	-2.32639700	-0.41148700	-0.30030000
H	-0.91958900	-1.72232100	0.14934200
H	1.57109900	0.03219300	1.51100300
H	1.16162300	-1.52022400	0.78505300
H	-1.22245800	1.87170100	0.67890900
H	0.51183100	2.14520100	0.34739100
H	-0.63086200	1.80155200	-0.97567000

2BO-int1

Atom	X	Y	Z
C	2.74126500	0.49027400	-0.00003300
C	3.66556200	-0.70125600	-0.00005000
C	2.99550400	-2.07494000	0.00014700
H	2.36624300	-2.21212600	-0.88574900
C	3.34103700	1.86929100	-0.00018400
H	4.43294000	1.81917000	-0.00062100
H	3.00938900	2.43245700	-0.88019600
H	3.01011900	2.43232500	0.88019300
N	1.47703800	0.27733500	0.00011600
O	0.71021200	1.45748800	0.00004000
H	-0.21040400	1.09181100	0.00006700
C	-2.74126500	-0.49027400	-0.00007300
C	-3.66556200	0.70125600	-0.00004100

C	-2.99550400	2.07494000	0.00018100
H	-2.36624600	2.21214400	-0.88571400
C	-3.34103700	-1.86929100	-0.00023000
H	-4.43294000	-1.81917000	-0.00063400
H	-3.00941600	-2.43244500	-0.88025900
H	-3.01009300	-2.43233700	0.88012900
N	-1.47703800	-0.27733500	0.00006200
O	-0.71021200	-1.45748900	0.00006800
H	0.21040400	-1.09181100	0.00011900
H	4.32726900	-0.60355800	-0.87351800
H	4.32746100	-0.60340200	0.87325500
H	3.75708200	-2.86210100	0.00014700
H	2.36641300	-2.21195500	0.88619000
H	-3.75708200	2.86210100	0.00020100
H	-2.36641000	2.21193700	0.88622400
H	-4.32727200	0.60357700	-0.87350900
H	-4.32745800	0.60338300	0.87326400

2BO-TS1

Atom	X	Y	Z
C	2.61966700	-0.52237600	0.00030900
C	3.55865800	0.65010200	0.00034700
C	2.92027900	2.03962000	0.00022700
H	2.29668400	2.19849100	0.88624600
C	3.15327300	-1.91953700	0.00045700
H	2.79260600	-2.46416200	0.88074600
H	2.79258300	-2.46437200	-0.87969000
H	4.24520900	-1.91634500	0.00045000
N	1.34949400	-0.32356000	0.00015600

O	0.46457700	-1.36157400	0.00012500
H	-0.53191500	-0.76022500	-0.00002200
C	-2.61607100	0.52041300	-0.00030800
C	-3.54829300	-0.66455800	-0.00034600
C	-2.89017400	-2.04513600	-0.00020600
H	-2.26269000	-2.19088900	0.88591000
C	-3.19479900	1.90562900	-0.00044300
H	-2.85466500	2.46740200	0.87827000
H	-2.85465200	2.46723400	-0.87926100
H	-4.28808500	1.87241100	-0.00045400
N	-1.34038900	0.33634800	-0.00017400
O	-0.49954200	1.40881600	-0.00015500
H	0.77546800	0.62866100	0.00003600
H	-3.65840900	-2.82590500	-0.00024300
H	-2.26251100	-2.19098400	-0.88618000
H	-4.21174100	-0.56944800	0.87278800
H	-4.21155600	-0.56953600	-0.87363000
H	4.21630300	0.53221900	0.87357000
H	4.21647100	0.53214400	-0.87273800
H	3.70477500	2.80248200	0.00027500
H	2.29686200	2.19842000	-0.88593000

2BO-int2

Atom	X	Y	Z
C	-2.86514700	-2.09590700	-0.00011000
C	-3.60189200	-0.75427500	0.00020500
H	-2.23189300	-2.21318000	0.88617200
C	-2.75077700	0.48514000	0.00011400
C	-3.37909100	1.84050700	0.00026000

H	-3.05933900	2.41350200	0.87935700
H	-3.06051500	2.41312800	-0.87951700
H	-4.46893800	1.76161900	0.00097100
O	-0.62164600	1.42397400	-0.00031400
N	-1.45810600	0.39765600	-0.00014800
H	-0.96364200	-0.51904300	-0.00022600
C	2.86514400	2.09590700	-0.00011100
C	3.60189000	0.75427700	0.00018300
H	2.23190800	2.21317500	0.88618400
C	2.75077800	-0.48514000	0.00010600
C	3.37909400	-1.84050500	0.00025200
H	3.05940300	-2.41347200	0.87938900
H	3.06045900	-2.41315600	-0.87948500
H	4.46894100	-1.76161500	0.00089000
O	0.62164800	-1.42397600	-0.00028600
N	1.45810700	-0.39765700	-0.00014000
H	0.96364000	0.51904100	-0.00022300
H	-3.59311100	-2.91319700	-0.00004100
H	-2.23228700	-2.21297500	-0.88670100
H	-4.26815600	-0.69416700	-0.87293100
H	-4.26774100	-0.69434600	0.87367000
H	3.59310600	2.91319900	-0.00005400
H	2.23226400	2.21297900	-0.88668900
H	4.26813800	0.69417300	-0.87296600
H	4.26775700	0.69434500	0.87363500

AOU-int2

Atom	X	Y	Z
C	-1.31976900	0.58679600	-0.26037800

C	-1.53221000	-0.82391200	0.16400900
C	-2.80348100	-1.36593900	0.73267900
O	-2.44676900	1.28306500	-0.34979700
O	-3.52909800	-0.62491700	1.38369900
C	-3.15777500	-2.81053400	0.48590800
H	-2.83619700	-3.14921900	-0.50336600
H	-4.23794800	-2.92691300	0.60395700
H	-2.64511500	-3.44117900	1.22090300
O	-0.20860000	1.02195600	-0.53892600
N	-0.45344400	-1.57205300	0.00747800
O	-0.29371700	-2.79172000	0.27741700
H	0.39377100	-1.08952500	-0.36402500
C	-2.32584000	2.67167300	-0.77773100
H	-1.65422200	3.18224300	-0.08234000
H	-1.87176300	2.68200400	-1.77250400
C	-3.71880300	3.26487100	-0.77361600
H	-4.15734000	3.22903300	0.22865200
H	-3.66496600	4.31206400	-1.09091600
H	-4.37662600	2.72827400	-1.46496700
C	2.81764800	-2.26898900	-0.66235000
C	2.88837800	0.18995000	-0.93764300
H	2.17234900	0.76392300	-1.52798400
H	3.83507700	0.14539400	-1.48619800
C	3.07680100	0.83986400	0.43268200
H	2.11801200	0.82200300	0.96470500
H	3.78847800	0.24513800	1.02118000
C	3.57557700	2.28382300	0.31231800
H	2.86030900	2.86347900	-0.28730300
H	4.52772700	2.29853900	-0.23651700

C	3.76036300	2.95325800	1.67768400
H	4.11461100	3.98496000	1.56964800
H	2.81463800	2.97848000	2.23330700
H	4.49193800	2.40878300	2.28781800
O	3.17380200	-3.38666600	-0.52339900
N	2.33873000	-1.16743300	-0.81710500

AOU-TS2

Atom	X	Y	Z
C	1.48370400	0.77831700	-0.36068900
C	1.51851300	-0.69191800	-0.08356200
C	2.78675600	-1.46692400	0.12698600
O	2.60106500	1.39156000	0.02791800
O	3.80266700	-1.12145700	-0.46176200
C	2.75965400	-2.66352000	1.04283400
H	2.11453700	-2.49588000	1.91067100
H	3.78137400	-2.88984700	1.35710200
H	2.34874300	-3.52417200	0.50263700
O	0.51405200	1.34562500	-0.84166800
N	0.33232000	-1.23048000	-0.08899500
O	0.03866300	-2.46111300	0.11650200
H	-0.85178900	-0.76198600	-0.32745600
C	2.66990200	2.82979400	-0.18794200
H	2.52777900	3.01919500	-1.25549700
H	1.84717800	3.29698900	0.36061600
C	4.02528600	3.29415500	0.30272500
H	4.83199600	2.80268700	-0.25044400
H	4.11219300	4.37601400	0.15355500
H	4.15068200	3.08211800	1.36961700

C	-2.04001000	-2.40007100	-0.12910800
C	-3.22925200	-0.33851700	-0.66362500
H	-4.13098300	-0.94688500	-0.54157700
H	-3.16624900	-0.02694900	-1.71149100
C	-3.25268300	0.87786300	0.25885200
H	-3.29992800	0.53677000	1.30151200
H	-2.31091300	1.43011200	0.14251300
C	-4.43760900	1.80189900	-0.04343100
H	-5.37586000	1.23978600	0.06333800
H	-4.38641300	2.12672800	-1.09187300
C	-4.46825300	3.02946900	0.87229700
H	-5.32164200	3.67660000	0.63930200
H	-4.54874300	2.73308900	1.92564900
H	-3.55376700	3.62536000	0.76135700
O	-2.41679600	-3.50210800	0.05762000
N	-2.04395400	-1.17281500	-0.38392400

OU-int2

Atom	X	Y	Z
C	-3.57198400	0.57019000	-0.70587400
C	-2.38048000	-0.03339200	-0.04045900
C	-2.07889800	0.17649600	1.41495200
C	-1.72533700	1.63909600	1.74227800
H	-0.83085400	1.95875600	1.19635100
H	-1.53272100	1.75166500	2.81464000
H	-2.54317000	2.31626200	1.47334400
C	1.58518400	-2.62213800	0.28469300
C	1.77314000	-0.22213600	0.88245800
H	2.75828500	-0.39319100	1.32797900

H	1.13178700	0.22037400	1.65043700
C	1.87365700	0.71251300	-0.32381000
H	0.88168500	0.82465900	-0.77948800
H	2.52015100	0.25218200	-1.08205200
C	2.42212800	2.08839200	0.06918200
H	3.41305000	1.96945400	0.52900400
H	1.77422900	2.53223100	0.83815700
N	-1.56191200	-0.74811600	-0.75807400
O	-1.66097300	-1.00184300	-2.03251700
O	1.85811200	-3.74649300	0.04430700
N	1.17350700	-1.50888700	0.51595900
C	2.52148800	3.03975000	-1.12707200
H	1.53742800	3.19905900	-1.58506100
H	2.91461200	4.01710600	-0.82458100
H	3.18685800	2.63327200	-1.89875900
H	-0.73721500	-1.15147800	-0.28983900
H	-3.26547600	1.29665900	-1.46976300
H	-4.15149400	-0.20245600	-1.22564700
H	-4.21203100	1.07248500	0.02320300
H	-2.95828400	-0.12110400	2.00195100
H	-1.25852000	-0.48286200	1.72252200

OU-TS2

Atom	X	Y	Z
C	-4.06559600	-0.45634300	-0.28632500
C	-2.84851900	0.26769700	0.18684700
C	-2.90179200	1.68415200	0.67892600
C	-3.30463100	2.67169100	-0.43144300
H	-2.56966300	2.66640000	-1.24343100

H	-3.36095100	3.68628200	-0.02420400
H	-4.28378900	2.42244900	-0.85388300
C	0.39710500	-1.92997100	-0.06641100
C	2.24150200	-0.68503700	0.71304400
H	2.79671900	-1.61015300	0.50548400
H	2.34507700	-0.47293600	1.78458300
C	2.82971400	0.47117600	-0.09680400
H	2.24420200	1.37915100	0.10431700
H	2.71506800	0.25191300	-1.16732600
C	4.30623800	0.72321300	0.22683900
H	4.88320700	-0.19157400	0.03070000
H	4.41226400	0.93126800	1.30112100
N	-1.70522700	-0.33238200	0.15306600
O	-1.49106800	-1.56569600	-0.27304800
O	0.50563000	-3.04917000	-0.47957900
N	0.80885700	-0.85593200	0.42698600
C	4.89839000	1.88338300	-0.57960500
H	4.35943300	2.81797900	-0.37841500
H	5.95399900	2.04464400	-0.33109200
H	4.83348000	1.68679800	-1.65725800
H	-0.80876100	0.09517200	0.46956200
H	-4.01116800	-0.63058800	-1.36840200
H	-4.13144500	-1.44030900	0.19096000
H	-4.96829000	0.11729300	-0.06693300
H	-3.63468200	1.73378900	1.49490500
H	-1.93106900	1.96978000	1.09954400

***n*-butyl isocyanate 1**

Atom	X	Y	Z

C	2.36653200	-0.18014900	-0.00582700
C	0.22273400	1.11603700	0.04733400
H	-0.01092900	1.87709600	-0.70322200
H	0.12474600	1.58935500	1.03129400
C	-0.73303300	-0.07238500	-0.07691900
H	-0.47175700	-0.82261100	0.68188400
H	-0.59098600	-0.54424200	-1.05815700
C	-2.19677900	0.34887200	0.09203900
H	-2.44681600	1.10509300	-0.66497000
H	-2.32605500	0.83252800	1.07025700
O	3.20890000	-1.01535100	0.07078000
N	1.61554200	0.74717700	-0.13336800
C	-3.16350500	-0.83361100	-0.02636700
H	-2.95465900	-1.59120700	0.73923200
H	-4.20317400	-0.50915700	0.09690200
H	-3.07605500	-1.31687300	-1.00744100
